# Rare Polyene-polyol Macrolides from Mangrove-derived *Streptomyces* sp. ZQ4BG

**DOI:** 10.1038/s41598-017-01912-z

**Published:** 2017-05-10

**Authors:** Wenling Wang, Tengfei Song, Weiyun Chai, Lu Chen, Lei Chen, Xiao-Yuan Lian, Zhizhen Zhang

**Affiliations:** 1 Ocean College, Zhoushan Campus, Zhejiang University, Zhoushan, 316021 China; 2 College of Pharmaceutical Sciences, Zhejiang University, Hangzhou, 310058 China

## Abstract

Bioactive natural products from mangrove-derived actinomycetes are important sources for discovery of drug lead compounds. In this study, an extract prepared from culture of an actinomycete *Streptomyces* sp. ZQ4BG isolated from mangrove soils was found to have activity in inhibiting proliferation of glioma cells. Large culture of this mangrove actinomycete in Gause’s liquid medium resulted in isolation of seven novel polyene-polyol macrolides, named as flavofungins III–IX (**3**–**9**), together with known flavofungins I (**1**) and II (**2**) and spectinabilin (**10**). Structures of these isolated compounds were elucidated by extensive NMR analyses and HRESIMS data. The stereochemical assignments were achieved by a combination of NOE information, universal NMR database, and chemical reactions including preparation of acetonide derivatives and Mosher esters. Flavofungins IV–VIII (**4**–**8**) are rare 32-membered polyene-polyol macrolides with a tetrahydrofuran ring, while flavofungin IX (**9**) represents the first example of this type of macrolide with a unique oxepane ring. Flavofungins I (**1**) and II (**2**) and spectinabilin (**10**) showed anti-glioma and antifungal activities.

## Introduction

Gliomas remain the most common and high death malignant brain tumors despite advances in therapies including chemotherapy, radiotherapy, and surgical resection^1–3^. Chemotherapy has played a more important role in treatment and prevention of gliomas, because gliomas usually locate at many important brain function areas, which makes the surgical resection extremely difficult. However, so far very few drugs have been approved for treating gliomas including temozolomide (TMZ), carmustine, and lomustine, and only TMZ has been independently used for treatment of gliomas^1^. Furthermore, the efficacy of TMZ and other current anti-glioma drugs remain unsatisfactory^1, 4^. Therefore, there is an urgent need to discover lead compounds for development of novel anti-glioma drugs. Bioactive natural products from mangrove-derived actinomycetes are important sources for discovery of drug lead compounds^5–7^. For example, the well-known salinosporamide A actually produced by a mangrove-derived *Salinospora* strain^8^ was the first and most advanced marine actinomycete secondary metabolite to be processed for clinical trials for cancer treatment.

During the course of our ongoing project to discover novel anti-glioma agents from marine resources^9–15^, ten bacterial strains were isolated from a sample of mangrove soils. An extract prepared from the culture of strain ZQ4BG was found to be the most active against the proliferation of glioma cells with an inhibition of 56.9% for U87MG and 48.9% for U251. This mangrove-derived strain was assigned as *Streptomyces* sp. ZQ4BG based on its 16S rDNA sequence (Supplementary Information, Fig. S1), which completely (99% identity for a 1404 bp stretch of sequence) matched those of two species of *Streptomyces* including *S*. *fulvissimus* and *S*. *flavofungini* (Table S1) in GenBank database. The actinomycete *S*. *flavofungini* was previously reported to produce polyene-polyol flavofungins^16^ and polyol macrolides of desertomycins^17^.

Polyene-polyol macrolides are usually microbial metabolites whose main structural elements are a continuously conjugated chain of four to seven unsubstituted double bonds connected to a polyol fragment with alternating hydroxy groups. Nystatin, produced by *Streptomyces noursei*, was the first polyene-polyol macrolide showing antifungal and antibacterial activities^18, 19^. Amphotericin B, a heptaene-polyol macrolide produced by *S*. *nodosus*, is a standard antifungal drug for treating serious deep-seated systemic fungal infections^20^. Most of the polyene-polyol macrolides discovered since are produced by a variety of terrestrial *Streptomyces* spp., such as mycoticins from *S*. *ruber* ^21^, faeriefungins from *S*. *griseus var*. *autotrophicus* ^19^, PK-397 from *Streptomyces* sp. 87–397^22–24^, nystatins from *S*. *noursei* ^25^, and reedsmycins from *Streptomyces* sp. CHQ-64^26^. Recently, polyene-polyol macrolides were also isolated from marine actinomycetes, such as marinisporolides from marine *Marinispora* sp. CNQ140^27^ and bahamaolides from marine *Streptomyces* sp^28^. Some of these polyene-polyol macrolides mentioned above were found to have significant antifungal, antibacterial, antiviral, and antitumor activities^18–28^.

A HPLC analysis of the active extract from strain ZQ4BG revealed that this mangrove-derived actinomycete produced rich metabolites with similar UV absorption (λ_max_) at 365 nm or 330 nm. Further LC/MS analysis of this extract showed a series of [M + Na]^+^ ions at *m*/*z* 673.4, 675.4, 687.4, 689.4, 703.4, and 707.4. The compounds with MS data at 673.4 and 687.4 with UV absorption at 365 nm were match to those of known flavofungins I (**1**) and II (**2**), respectively, in AntiBase database^29^ and references^30, 31^. The others with *m*/*z* 675.4, 689.4, 703.4, and 707.4 with λ_max_ 330 nm did not match any previously reported compounds, prompting us to characterize them further. Therefore, large culture of this isolated mangrove-derived strain ZQ4BG in Gause’s liquid medium resulted in isolation and identification of a series of polyene-polyol macrolides, including new flavofungins III–IX (**3**–**9**) and known flavofungins I (**1**) and II (**2**) and spectinabilin (**10**). Flavofungins IV–VIII (**4**–**8**) are rare 32-membered polyene-polyol macrolides with a tetrahydrofuran ring, while flavofungin IX (**9**) is the first example of this type of macrolide with a unique oxepane group. Herein, we report the isolation and culture of strain ZQ4BG, the structural elucidation of these novel flavofungins, and the anti-glioma and antimicrobial active evaluation of these isolated metabolites.

## Results and Discussion

**Figure 1:**
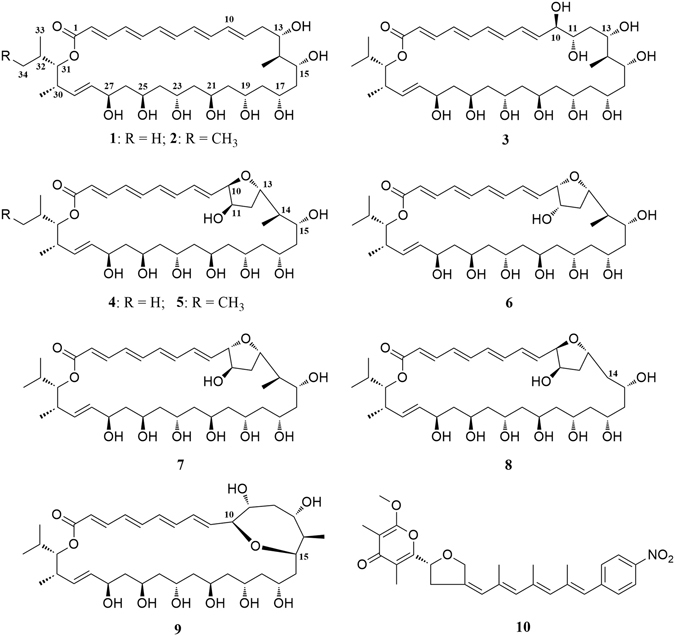
Structures of compounds (**1**–**10**) isolated from *Streptomyces* sp. ZQ4BG.

An actinomycete strain ZQ4BG which produced rich polyene-polyol macrolides was isolated from mangrove soils and assigned as *Streptomyces* sp. ZQ4BG based on the result of its 16S rDNA sequence. Culture (total 60 L) of this isolated actinomycete in Gause’s liquid medium resulted in isolation of compounds **1**–**10** (Fig. 1).

Compounds **1**, **2**, and **10** were proved to be known compounds of flavofungins I (**1**) and II (**2**)^30, 31^ and spectinabilin (**10**)^32–34^ based on their HRESIMS and NMR data (Tables 1 and 2) as well as a comparison to the data of references. Flavofungins I (**1**) and II (**2**) were 32-membered polyene-polyol macrolides with a conjugated chain of five unsubstituted double bonds connected to a polyol fragment with eight hydroxy groups alternated. Both flavofungins I (**1**) and II (**2**) were reported to have antifungal and antiviral activities^19^, while spectinabilin (**10**) was a nitro-substituted polyketide with activity to block the binding of androgen to the ligand-binding domain of androgen receptor^34^.

**Table 1:** ^13^C-NMR data of compounds **1**–**9** (125 MHz, in DMSO-*d*_6_)

No.	1	2	3	4	5	6	7	8	9
1	166.2	166.3	166.0	166.1	166.1	166.1	166.1	166.1	166.1
2	120.0	120.0	120.4	120.9	120.8	120.8	120.9	120.7	120.8
3	144.9	145.1	144.3	144.3	144.3	144.1	144.0	144.4	144.3
4	129.3	129.3	130.0	130.4	130.3	130.2	130.4	130.0	130.4
5	141.2	141.3	140.7	140.3	140.3	140.3	140.1	140.5	140.3
6	131.1	131.2	131.4	131.8	131.8	130.5	131.3	131.2	131.8
7	137.6	137.8	136.7	136.3	136.2	136.9	136.3	136.5	136.5
8	130.2	130.3	130.8	130.6	130.6	128.6	127.0	128.9	131.4
9	135.6	135.7	137.3	135.7	135.7	134.8	136.1	136.9	136.6
10	130.6	130.6	75.8	85.9	85.9	81.8	82.6	85.8	88.6
11	135.0	135.2	73.4	74.7	74.7	72.0	75.8	75.3	71.8
12	37.2	37.8	34.9	40.1	40.1	41.4	40.3	39.6	40.3
13	68.0	67.9	70.4	79.1	79.1	79.0	78.7	74.7	66.9
14	45.9	46.1	43.9	42.8	42.8	45.4	45.3	41.1	44.1
15	68.7	68.6	70.1	69.3	69.2	68.0	67.7	65.3	82.7
16	43.8	43.9	41.6	41.8	41.8	39.3	39.2	46.2^*a*^	44.1^*a*^
17	69.1	69.2	68.8	68.4	68.3	69.6	69.4	66.5	65.1
18	42.7	42.6	44.7^*a*^	45.9	45.9	45.1	45.1	46.5^*a*^	44.2^*a*^
19	67.3	67.5	66.0	66.3	66.2	66.7	66.4	65.2	64.7
20	46.6^*a*^	46.7^*a*^	45.3^*a*^	46.1^*a*^	46.1	47.5^*a*^	47.2^*a*^	45.1	46.9^*a*^
21	62.1	62.0	63.6	62.8	62.7	62.6	62.8	63.1	62.3
22	47.1^*a*^	47.2^*a*^	46.0^*a*^	46.9	46.9	47.3^*a*^	47.1^*a*^	46.7	47.2^*a*^
23	62.5	62.5	63.8	62.9	62.8	62.8	63.0	63.2	62.5
24	46.3^*a*^	46.3^*a*^	45.5^*a*^	46.2^*a*^	46.1	46.8	46.6	46.0	44.9^*a*^
25	66.2	66.1	65.4	65.8	65.8	64.0	64.2	65.4	65.6
26	45.5	45.5	45.7^*a*^	46.4^*a*^	46.3	47.9	47.6	46.3^*a*^	46.6^*a*^
27	69.5	69.5	69.1	69.1	69.0	67.4	67.6	68.8	68.9
28	133.3	133.3	130.7	133.3	133.3	133.2	133.2	133.3	133.2
29	129.9	130.0	133.7	129.6	129.6	129.3	129.3	129.7	129.3
30	35.3	35.3	36.1	35.5	35.4	35.4	35.5	35.6	35.1
31	79.7	77.9	80.2	79.5	77.8	78.9	79.0	79.4	79.3
32	28.9	35.0	29.0	29.0	35.1	29.1	29.0	28.9	29.0
33	18.4	14.5	18.7	18.7	14.6	18.8	18.8	18.7	18.5
34	19.6	25.2	19.4	19.7	25.1	19.6	19.6	19.6	19.7
35	—	10.4	—	—	10.5	—	—	—	—
Me-14	9.9	9.9	10.5	12.9	12.9	9.2	9.1	—	12.2
Me-30	10.8	10.6	12.2	11.1	10.8	11.2	11.3	11.3	10.7

^a^Data with the same labels in each column may be interchanged.

**Table 2:** ^1^H-NMR data of compounds **1**–**4** (500 MHz, in DMSO-*d*_6_, *J* = Hz).

No.	1	2	3	4
2	5.89, d (15.0)	5.89, d (15.0)	5.93, d (15.1)	5.92, d (15.1)
3	7.17, dd (15.0, 11.2)	7.17, dd (15.0, 11.7)	7.22, dd (15.1, 11.5)	7.17, dd (15.1, 11.4)
4	6.39, dd (14.6, 11.2)	6.39, dd (14.5, 11.7)	6.48, dd (14.8, 11.5)	6.49, dd (14.8, 11.4)
5	6.67, dd (14.6, 11.2)	6.66, dd (14.5, 11.5)	6.73, dd (14.8, 11.0)	6.67, dd (14.8, 11.1)
6	6.32, dd (14.6, 11.2)	6.33, dd (14.5, 11.5)	6.34, dd (14.9, 11.0)	6.32, dd (14.8, 11.1)
7	6.45, dd (14.6, 11.2)	6.45, dd (14.5, 11.0)	6.44, dd (14.9, 10.9)	6.44, dd (14.8, 11.3)
8	6.23, dd (14.6, 11.2)	6.22, dd (14.6, 11.0)	6.28, dd (15.1, 10.9)	6.25, dd (15.1, 11.3)
9	6.30, dd (14.6, 10.8)	6.29, dd (14.6, 11.0)	5.80, dd (15.1, 7.3)	5.82, dd (15.1, 6.6)
10	6.09, dd (15.0, 10.8)	6.09, dd (14.6, 11.0)	3.79, t (7.3)	3.97, m
11	5.84, m	5.84, m	3.51, m	3.87, m
12	2.27, m; 2.32, m	2.27, m; 2.32, m	1.22, m; 1.50, m	1.71, m; 1.79, m
13	4.15, m	4.15, m	3.85, m	3.85, m
14	1.57, m	1.56, m	1.57, m	1.61, m
15	3.32, m	3.31, m	3.41, m	3.70, m
16	1.31, m; 1.36, m	1.30, m; 1.35, m	1.30, m; 1.58, m	1.37, m; 1.63, m
17	3.85, m	3.84, m	3.78, m	3.70, m
18	1.00, m; 1.36, m	0.99, m; 1.35, m	1.20, m; 1.40, m	1.35, m; 1.49, m
19	3.90, m	3.88, m	3.88, m	3.88, m
20	0.95–1.08^*a*^	0.95–1.07^*a*^	1.22–1.35^*a*^	1.08–1.35^*a*^
21	3.90, m	3.88, m	3.93, m	3.95, m
22	0.95–1.08^*a*^	0.95–1.07^*a*^	1.22–1.35^*a*^	1.08–1.35^*a*^
23	3.90, m	3.88, m	3.90, m	3.89, m
24	0.95–1.08^*a*^	0.95–1.07^*a*^	1.22–1.35^*a*^	1.08–1.35^*a*^
25	3.80, m	3.80, m	3.74, m	3.75, m
26	1.12, m; 1.31, m	1.11, m; 1.30, m	1.22, m; 1.46, m	1.08, m; 1.35, m
27	4.02, m	4.02, m	3.98, m	4.09, m
28	5.37, dd (15.8, 4.2)	5.37, dd (15.8, 4.0)	5.41, dd (15.8, 5.1)	5.41, dd (15.6, 4.1)
29	5.43, dd (15.8, 4.7)	5.44, dd (15.8, 4.6)	5.36, dd (15.8, 5.5)	5.49, dd (15.6, 4.6)
30	2.54, m	2.53, m	2.53, m	2.54, m
31	4.66, dd (10.2, 2.2)	4.75, dd (10.2, 2.2)	4.63, dd (9.6, 2.3)	4.65, dd (10.0, 2.2)
32	1.80, m	1.62, m	1.84, m	1.83, m
33	0.91, d (6.7)	0.87, d (6.7)	0.90, d (6.7)	0.91, d (6.7)
34	0.80, d (6.7)	1.00, m; 1.26, m	0.79, d (6.7)	0.80, d (6.7)
35	—	0.80, t (7.6)	—	—
Me-14	0.73, d (6.8)	0.73, d (7.0)	0.75, d (6.9)	0.78, d (6.9)
Me-30	0.94, d (6.8)	0.94, d (6.8)	0.97, d (6.7)	0.97, d (6.8)
OH-10	—	—	4.73, br.s	—
OH-11	—	—	4.91, br.s	5.12, d (3.1)
OH-13	4.39, d (3.3)	4.38, d (2.8)	4.45, br.s	—
OH-15	4.27, d (6.6)	4.26, d (6.5)	4.52, br.s	4.30, d (4.5)
OH-17	4.84, br.s	4.84, br.s	4.73, br.s	4.48, d (3.9)
OH-19	4.61, d (2.8)	4.61, br.s	4.47, br.s	4.37, d (4.1)
OH-21	3.99, d (5.8)	3.99, d (6.5)	4.21, d (5.0)	4.06, d (5.9)
OH-23	3.98, d (5.8)	3.97, d (6.5)	4.20, d (5.2)	4.14, d (5.3)
OH-25	4.50, d (3.8)	4.49, d (3.6)	4.32, br.s	4.42, d (4.5)
OH-27	4.78, br.s	4.77, br.s	4.63, br.s	4.69, d (2.6)

^a^Data with the same labels in each column could not be individually assigned.

Compounds **3**–**9** are new members of flavofungins I (**1**) and II (**2**), with only four unsubstituted conjugated double bonds and more oxygenated moieties, and named as flavofungins III–IX (**3**–**9**). Flavofungin III (**3**) is an analogue of flavofungin I (**1**) with two hydroxy groups substituted at C-10 and C-11. Flavofungins IV–IX (**4**–**9**) are rare 32-membered polyene-polyol macrolides with a tetrahydrofuran (THF) ring between C-10 and C-13 for flavofungins IV–VIII (**4**–**8**) or an oxepane ring between C-10 and C-15 for flavofungin IX (**9**). It has been found that new flavofungins III–IX (**3**–**9**) might derive from known flavofungins I (**1**) and II (**2**), suggesting all flavofungins produced by strain ZQ4BG might have same biosynthetic pathway. A hypothetical mechanism for the formation of **3**–**9** from **1** and **2** is proposed in Fig. S2 (Supplementary Information). The 10,11-epoxide compounds (Fig. S2B) might be formed by enzymatic epoxidation of the double bond at C_10_ and C_11_ (Fig. S2A), followed by hydrolysis of these epoxides to give corresponding 10,11-dihydroxylated compounds (Fig. S2C)^35, 36^. New flavofungins IV-IX (**4**–**9**, Fig. S2D and E) might derive from 10,11-dihydroxylated compounds by cyclization at C-10 and C-13 or C-10 and C-15. The exact structures of these new flavofungins were elucidated based on their HRESIMS data and extensive NMR spectroscopic analyses. The stereochemical assignments were made by a combination of NOE information, ^1^H and ^13^C NMR chemical shifts, universal NMR database^27, 37–39^, and chemical reactions including preparations of acetonide derivatives^27, 28, 40^ and Mosher esters.

Flavofungins IV (**4**), VI (**6**), and VII (**7**) had a same molecular formula of C_36_H_58_O_11_ deduced from their HRESIMS ions at *m*/*z* [M + Na]^+^ 689.3870, 689.3872, 689.3867 and their ^13^C data. Extensive NMR spectroscopic analyses including ^1^H, ^13^C, ^1^H-^1^H COSY, HSQC, and HMBC concluded that these three compounds shared a same planar structure, which is a 32-membered macrolide with four unsubstituted conjugated double bonds at C-2 to C-9, one double bond at C-28 and C-29, 11 oxymethines at C-10, C-11, C-13, C-15, C-17, C-19, C-21, C-23, C-25, C-27 and C-31, two methyls at C-14 and C-30, one isopropyl at C-31, and a tetrahydrofuran (THF) ring between C-10 and C-13. All double bonds in **4**, **6**, and **7** were assigned as *E* geometries based on their *trans*-coupling constants (≥14.8 Hz, Tables 2 and 3). As mentioned above, flavofungins IV (**4**), VI (**6**), and VII (**7**) might derive from flavofungin I (**1**) by enzymatic epoxidation and hydrolysis with different configurations at C-10 and C-11. The big differences of the coupling constants of ^3^ *J*_H9−10_ and ^3^ *J*_H10−11_ and the ^13^C NMR chemical shifts of C-10 and C-11 in **4**, **6**, and **7** were observed, supporting that the structural differences among **4**, **6**, and **7** were from their different configurations at C-10 and C-11.

**Table 3:** ^1^H-NMR data of compounds **5**–**9** (500 MHz, in DMSO-*d*_6_, *J* = Hz)

No.	5	6	7	8	9
2	5.91, d (15.2)	5.90, d (15.1)	5.91, d (15.1)	5.92, d (15.0)	5.85, d (15.1)
3	7.17, dd (15.2, 11.2)	7.16, dd (15.1, 11.2)	7.17, dd (15.1, 11.4)	7.17, dd (15.0, 11.1)	7.16, dd (15.1, 11.4)
4	6.47, dd (14.7, 11.2)	6.46, dd (15.0, 11.2)	6.47, dd (15.1, 11.4)	6.47, dd (15.0, 11.1)	6.44, dd (14.9, 11.4)
5	6.68, dd (14.7, 11.1)	6.69, dd (15.0, 11.2)	6.69, dd (15.1, 11.5)	6.68, dd (15.0, 11.1)	6.68, dd (14.9, 11.0)
6	6.31, dd (14.6, 11.1)	6.23, dd (15.1, 11.2)	6.26, dd (15.1, 11.5)	6.28, dd (15.0, 11.1)	6.27, dd (14.9, 11.0)
7	6.44, dd (14.6, 11.0)	6.49, dd (15.1, 10.9)	6.47, dd (15.1, 11.5)	6.44, dd (15.0, 11.2)	6.41, dd (14.9, 10.8)
8	6.26, dd (14.9, 11.0)	6.28, dd (14.9, 10.9)	6.26, dd (15.1, 11.5)	6.25, dd (14.9, 11.2)	6.18, dd (15.0, 10.8)
9	5.82, dd (14.9, 6.6)	5.86, dd (14.9, 3.5)	6.01, dd (15.1, 3.5)	5.89, dd (14.9, 6.6)	5.72, dd (15.0, 7.3)
10	3.96, m	4.43, m	4.10, m	4.16, m	3.39, t (7.3)
11	3.86, m	4.23, m	3.88, m	3.94, m	3.32, m
12	1.72, m; 1.79, m	1.65, m; 1.95, m	1.51, m; 2.24, m	1.65, m; 1.77, m	1.70, m; 1.89, m
13	3.85, m	3.76, m	3.62, m	4.34, m	3.89, m
14	1.61, m	1.58, m	1.75, m	1.66, m	1.71, m
15	3.69, m	3.87, m	3.88, m	3.74, m	3.12, m
16	1.36, m; 1.63, m	1.34, m; 1.43, m	1.30, m; 1.42, m	1.47, m; 1.52, m	1.45, m; 1.61, m
17	3.69, m	3.83, m	3.83, m	3.57, m	3.67, m
18	1.36, m; 1.49, m	1.39, m; 1.67, m	1.39, m; 1.64, m	1.09–1.36^*a*^	1.37, m; 1.49, m
19	3.87, m	3.94, m	3.93, m	3.89, m	3.89, m
20	1.08–1.35^*a*^	1.14, m; 1.35, m	1.15, m; 1.35, m	1.15, 1.41	0.97–1.31^*a*^
21	3.95, m	4.02, m	4.01, m	3.98, m	3.89, m
22	1.08–1.35^*a*^	1.20, m; 1.35, m	1.20, m; 1.35, m	1.09–1.36^*a*^	0.97–1.31^*a*^
23	3.89, m	3.89, m	3.88, m	3.91, m	3.89, m
24	1.08–1.35^*a*^	1.02 m; 1.35, m	1.04 m; 1.33, m	1.09–1.36^*a*^	0.97–1.31^*a*^
25	3.75, m	3.66, m	3.66, m	3.74, m	3.71, m
26	1.08, m; 1.34, m	0.93, m; 1.49, m	0.97, m; 1.48, m	1.06, m; 1.37, m	1.05, m; 1.29, m
27	4.08, m	4.12, m	4.12, m	4.09, m	4.11, m
28	5.47, dd (15.7, 4.0)	5.51, dd (15.9, 3.3)	5.49, dd (15.8, 3.5)	5.42, dd (15.9, 4.3)	5.43, dd (15.7, 3.6)
29	5.50, dd (15.7, 4.7)	5.54, dd (15.9, 4.6)	5.54, dd (15.8, 4.5)	5.48, dd (15.9, 5.2)	5.52, dd (15.7, 4.5)
30	2.54, m	2.56, m	2.57, m	2.55, m	2.57, m
31	4.74, dd (10.3, 2.2)	4.66, dd (9.8, 2.3)	4.66, dd (9.7, 2.3)	4.66, dd (9.6, 2.2)	4.64, dd (10.2, 2.2)
32	1.63, m	1.85, m	1.84, m	1.84, m	1.82, m
33	0.88, d (6.7)	0.91, d (6.7)	0.91, d (6.7)	0.91, d (6.7)	0.92, d (6.8)
34	1.00, m; 1.35, m	0.79, d (6.7)	0.79, d (6.7)	0.80, d (6.7)	0.80, d (6.8)
35	0.80, t (7.5)	—	—	—	—
Me-14	0.77, d (6.9)	0.68, d (6.8)	0.68, d (6.9)	—	0.78, d (6.9)
Me-30	0.97, d (6.9)	0.99, d (6.9)	0.99, d (6.9)	0.97, d (6.7)	0.97, d (6.9)
OH-11	5.12, d (4.4)	4.68, br.s	5.27, br.s	5.07, br.s	4.66, br.s
OH-13	—	—	—	—	4.42, d (3.9)
OH-15	4.30, d (4.8)	4.41, d (4.8)	4.44, br.s	4.34, d (4.2)	—
OH-17	4.47, d (4.4)	4.81, d (2.5)	4.81, br.s	4.38, d (4.5)	4.25, d (4.4)
OH-19	4.33, d (4.5)	4.67, br.s	4.59, br.s	4.28, d (5.0)	4.42, d (3.9)
OH-21	4.02, d (5.8)	4.11, d (4.9)	4.09, d (5.0)	4.07, d (5.6)	3.97, d (4.7)
OH-23	4.12, d (5.5)	4.19, d (4.9)	4.19, d (5.2)	4.16, d (5.0)	4.17, d (4.9)
OH-25	4.39, d (5.2)	4.18, d (5.0)	4.17, d (5.0)	4.32, d (4.2)	4.41, d (4.7)
OH-27	4.69, d (3.3)	4.46, d (3.7)	4.47, br.s	4.62, br.s	4.68, br.s

^a^The data with the same labels in each column could not be individually assigned.

**Figure 2:**
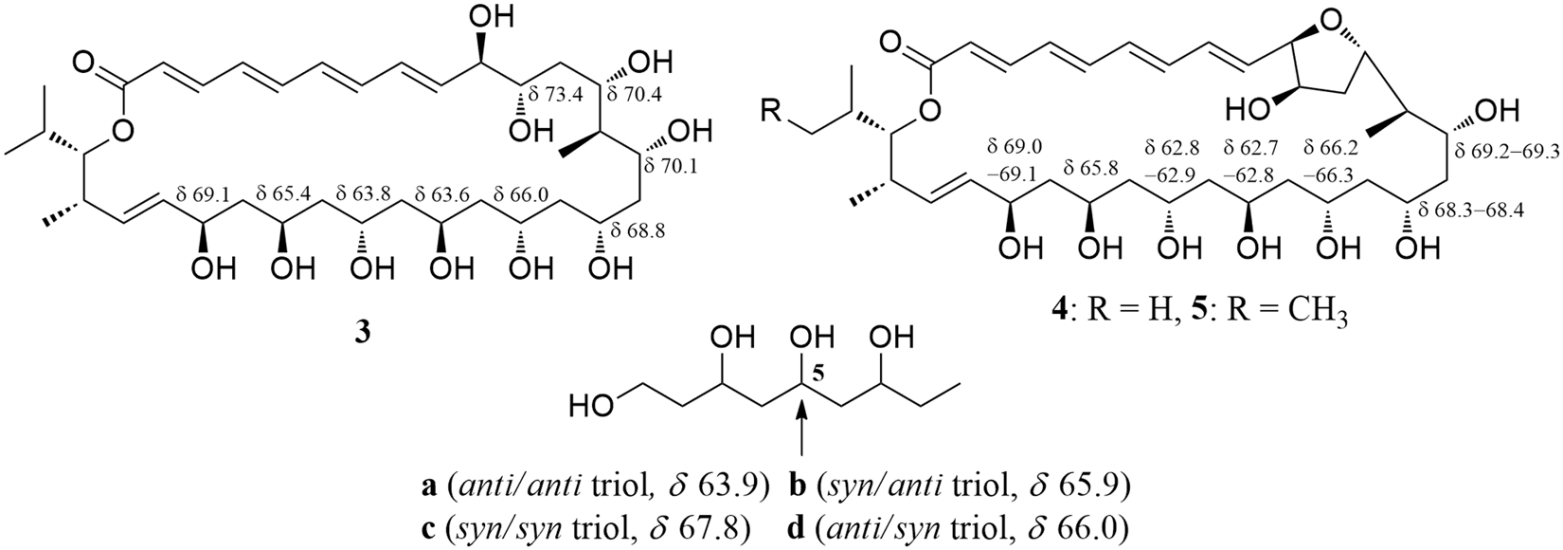
^13^C NMR chemical shifts of the central carbon in 1,3,5-triols (**a**–**d**) and the relevant portion of flavofungins III–V (**3**–**5**).

**Figure 3:**
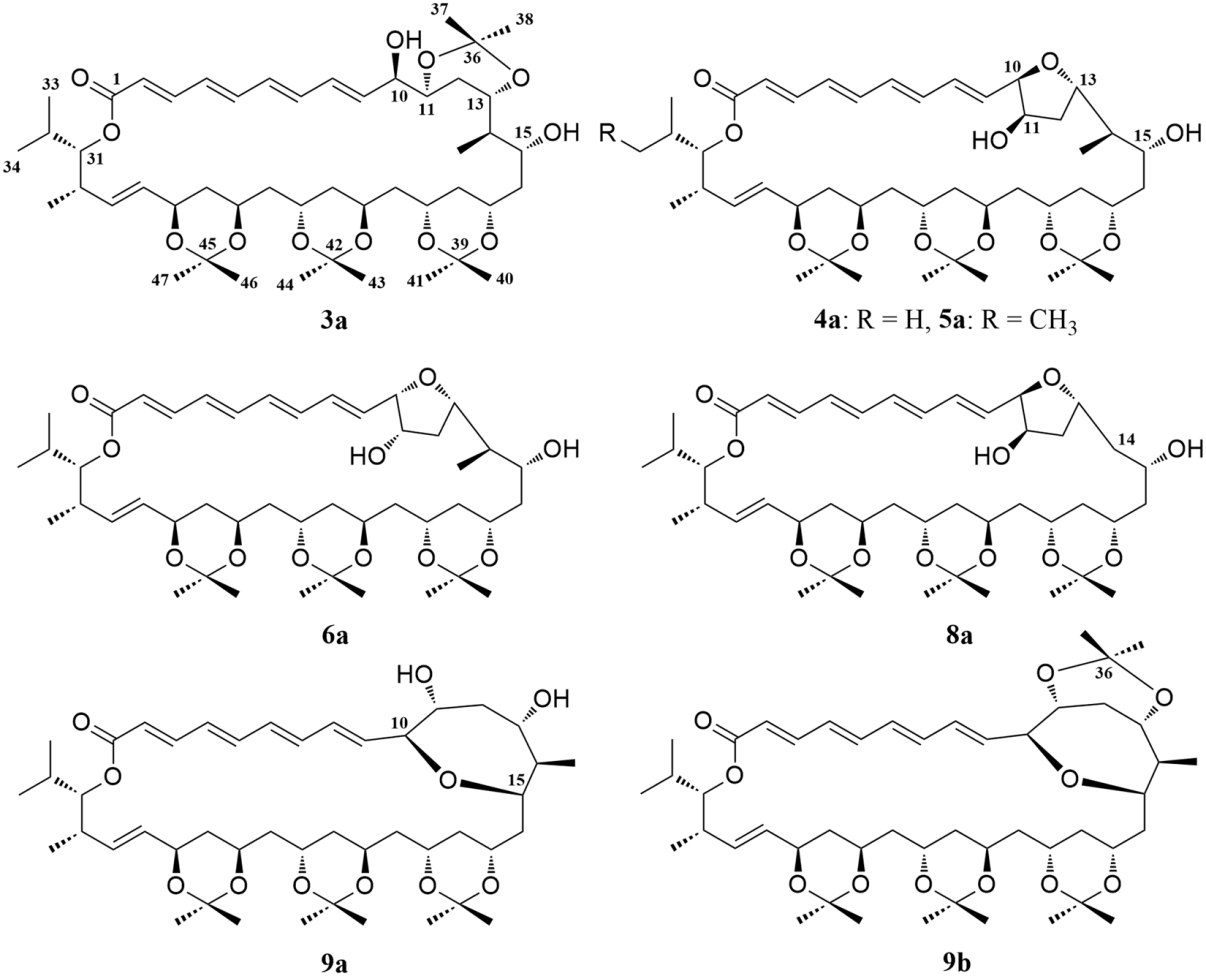
Acetonide derivatives 3a–6a, 8a, 9a, and 9b of flavofungins III–VI (**3**–**6**), VIII (**8**), and IX (**9**).

**Figure 4:**
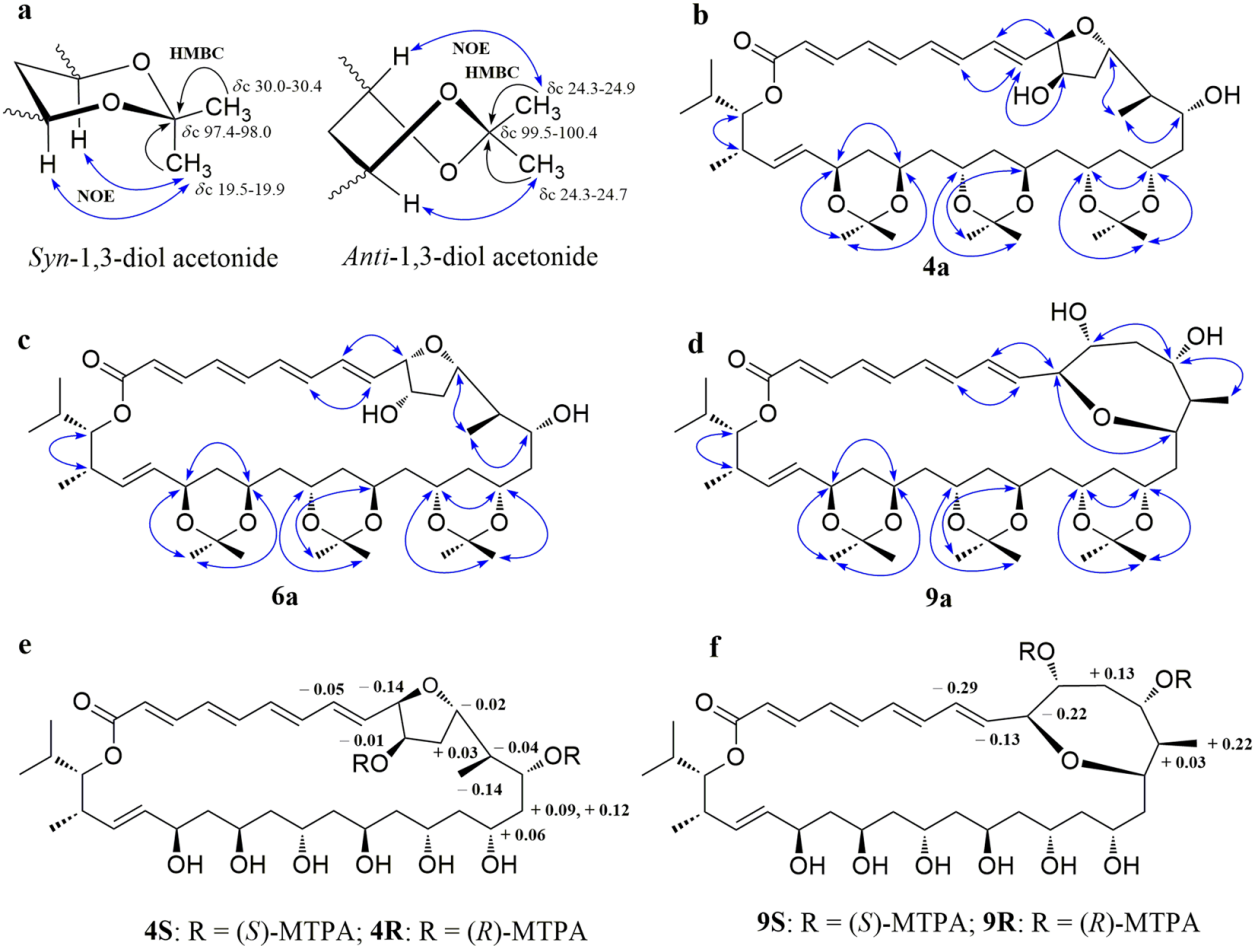
(**a**) Overview of configurational assignments of polyol chains using ^13^C NMR chemical shifts, HMBC, and NOE correlations of 1,3-diol acetonides. (**b**–**d**) Key NOE correlations of triacetonide derivatives 4a, 6a, and 9a. (**e**,**f**) Δδ_*S*-*R*_ values for the Mosher diesters 4S, 4R, 9S, and 9R.

The relative configurations of C-15, C-17, C-19, C-21, C-23, C-25, and C-27 in **4** were initially suggested by comparison of the ^13^C NMR data from **4** with the Kishi’s ^13^C Universal NMR database of model compounds (**a**–**d**, Fig. 2)^27, 37–39^. As shown in Table 1 and Fig. 2, the ^13^C NMR chemical shifts of C-17 (*δ* 68.4), C-19 (*δ* 66.3), C-21 (*δ* 62.8), C-23 (*δ* 62.9), and C-25 (*δ* 65.8) in **4** were close to the C-5 values at *δ* 67.8 (*syn*/*syn* triol), *δ* 66.0 (*anti*/*syn* triol), *δ* 63.9 (*anti*/*anti* triol), *δ* 63.9 (*anti*/*anti* triol), and *δ* 65.9 (*syn*/*anti* triol), respectively, in the Kishi Database. Thus, the relative configurations of the 1,3-diols were assigned as 15,17-*syn*, 17,19-*syn*, 19,21-*anti*, 21,23-*anti*, 23,25-*anti*, and 25,27-*syn*. The relative configurations of these 1,3-diols were also supported by the ^13^C NMR chemical shifts of the dimethyl and acetal carbons in the triacetonide derivative **4a** (Fig. 3) prepared from flavofungin IV (**4**). Usually, two distinctive NMR chemical shifts around *δ*_C_ 20 and 30 for dimethyl and *δ*_C_ 98 for acetal suggest a 1,3-*syn* diol, while a 1,3-*anti* diol is indicated by two identical signals near *δ*_C_ 25 for dimethyl and *δ*_C_ 100 for acetal^27, 28, 40^. The ^13^C NMR chemical shifts (Table S3) of the acetonide methyl and acetal carbons in **4a** were observed at *δ* 19.9 (C-40), 30.2 (C-41), and 98.0 (C-39) for 17,19-*syn*, *δ* 24.5 (C-43), 24.6 (C-44), and 100.1 (C-42) for 21,23-*anti*, and *δ* 19.9 (C-47), 30.3 (C-46), and 98.1 (C-45) for 25,27-*syn* (Fig. 3). NOE correlations (Fig. 4B) of H-40 (*δ* 1.35) with H-17 (*δ* 3.99) and H-19 (*δ* 3.86), H-43 (*δ* 1.25) with H-23 (*δ* 3.74), H-44 (*δ* 1.20) with H-21 (*δ* 3.80), and H-47 (*δ* 1.31) with H-25 (*δ* 3.82) and H-27 (*δ* 4.24) further confirmed the configurations of C-17, C-19, C-21, C-23, C-25, and C-27. The absolute stereochemistry of C-11 and C-15 was next determined by application of the Modified Mosher ester NMR method^27, 28^. Treatment of **4a** with (*S*)-(+)-α-methoxy-α-(trifluoromethyl) phenylacetyl chloride [(*S*)-(+)-MTPA-Cl] or (*R*)-(−)-MTPA-Cl gave *di*-*S*-Mosher ester **4S** or *di*-*R*-Mosher ester **4R**. The ^1^H NMR chemical shift differences (Δ*δ*_*S*-*R*_, Fig. 4E, Table S6) between **4S** and **4R** in negative values for H-8 to H-10 and positive value for H-12 were observed, suggesting a 11*R* configuration, while negativeΔ*δ*_*S*-*R*_ values for Me-14 and H-14 and positiveΔ*δ*_*S*-*R*_ values for H-16 and H-17 indicated a 15*R* configuration. Thus, the absolute configurations of 17*R*, 19*R*, 21*S*, 23*S*, 25*R*, and 27*R* were assigned based on a 15*R* configuration and the relative configurations of these 1,3-diols. NOE correlations of Me-14 with H-13 and H-15 observed in NOESY spectra of both **4** and **4a** determined that these protons had a same *β*-orientation, suggesting 13*S* and 14*R* conformations. The ^13^C NMR chemical shifts for C-30 to C-34 and the ^1^H NMR chemical shifts for H-30 to H-34 in compound **4** and flavofungin I (**1**) were superimposable, suggesting the two compounds had same 30*S* and 31*S* configurations. A small coupling constant (^3^ *J*_H30−31_, 2.2 Hz) and a large coupling constant (^3^ *J*_H31−32_, 10.0 Hz), as well as NOE correlation of H-31 (*δ* 4.65, dd, *J* = 10.0, 2.2 Hz) with H-30 (*δ* 2.54, m) and no NOE between H-31 and H-32 (*δ* 1.83, m), also supported the 30*S* and 31*S* configurations in **4**.

As mentioned above, compounds **4**, **6**, and **7** are stereoisomers and their structural differences were from their different configurations at C-10 and C-11. Because the 11*R* configuration in **4** had already been assigned, the conformations of C-10 and C-11 in compounds **4**, **6**, and **7** could be established based on their different coupling constants of ^3^ *J*_H9−10_ and ^3^ *J*_H10−11_ and different ^13^C NMR chemical shifts for C-10 and C-11 as well as NOEs. First, a large coupling constant (^3^ *J*_H9−10_, 6.6 or 7.5 Hz) in **4** and **4a** and a small coupling constant (^3^ *J*_H10−11_, 2.9 Hz) in **4a** (Tables 2 and S4) indicated an *α*-orientation for both H-10 and H-11, suggesting a 10*R* configuration at C-10 in **4**, while a *β*-orientation for both H-10 and H-11 in **6** was indicated by small coupling constants of ^3^ *J*_H9−10_ (3.5 or 5.7 Hz) in **6** and **6a** and ^3^ *J*_H10−11_ (3.9 Hz) in **6a** (Tables 3 and S5). Second, the ^13^C NMR chemical shifts (Table 1) at *δ* 81.8 (C-10) and *δ* 72.0 (C-11) in **6** were quite different from their counterparts at *δ* 85.9 (C-10) and *δ* 74.7 (C-11) in **4**. Furthermore, H-9 (*δ* 5.79) and H-11 (*δ* 3.89) exhibited NOE correlation in NOESY spectrum of **4a** (Fig. 4B), no NOE between H-9 (*δ* 5.93) and H-11 (*δ* 4.20) was observed in NOESY spectrum of **6a**. Altogether, the data demonstrated that compounds **4** and **6** had different configurations at C-10 and C-11, which were 10*R*, 11*R* for **4** and 10*S*, 11*S* for **6**. By comparison to **4** and **6**, a small ^3^ *J*_H9−10_ (3.5 Hz) coupling constant, NOE correlation of H-9 (*δ* 6.01) with H-11 (*δ* 3.88) and no NOE between H-10 (*δ* 4.10) and H-11, as well as ^13^C NMR chemical shifts at *δ* 82.6 (C-10) and *δ* 75.8 (C-11) suggested that **7** had 10*S* and 11*R* configurations. Based on the foregoing evidences, the structures of compounds **4**, **6**, and **7** were elucidated as novel polyene-polyol macrolides, named as flavofungin IV (**4**), VI (**6**), and VII (**7**). Their ^13^C and ^1^H NMR data were fully assigned by interpretation of ^1^H, ^13^C, ^1^H-^1^H COSY, HSQC, HMBC, and NOESY spectra and are listed in Tables 1–3.

Compound **5** gave a [M + Na]^+^ ion at *m*/*z* 703.4022, 14 mass units higher than that of **4**, implying the presence of an additional -CH_2_ moiety in **5**. Detailed analyses of extensive NMR spectra of **5** and its triacetonide derivative **5a** concluded that **5** was an analogue of **4**, with a –CH_2_CH_3_ moiety at C-34, instead of the –CH_3_ group at C-34 in **4**. The ^13^C and ^1^H NMR data (Tables 1, 3, S3 and S4) of **5** and **5a** were assigned based on ^1^H, ^13^C, ^1^H-^1^H COSY, HSQC, HMBC, and NOESY spectroscopic analyses. The structure of **5** was assigned as a new polyene-polyol macrolide, named as flavofungin V.

Compound **8** had a molecular formula of C_35_H_56_O_11_ deduced from its [M + Na]^+^ ion at *m*/*z* 675.3714, 14 mass units lower than that of **4**. Compared to **4**, the NMR spectra of **8** showed only three methyls. Further ^13^C and ^1^H NMR assignments (Tables 1, 3, S3 and S5) of **8** and its triacetonide derivative **8a** confirmed that **8** was very close to **4**, with the absence of a methyl at C-14. The structure of **8** was thus elucidated as a new polyene-polyol macrolide, named as flavofungin VIII.

The NMR spectra of **9** showed 36 carbons for a carbonyl, ten olefinic methines, 11 oxymethines, three methines, seven methenes, and four methyls, which were same to those of **4**, **6**, and **7**. Extensive NMR analyses confirmed that their structural differences were the tetrahydrofuran (THF) ring in **4**, **6**, and **7** replaced by an oxepane ring in **9**. Treatment of **9** with 2,2-dimethoxypropane afforded triacetonide **9a** and tetraacetonide **9b**. Interpretation of the ^13^C NMR chemical shifts (Table S3) of the acetonide methyl and acetal carbons in **9a** and **9b** resulted in the configurational assignments of 11,13-*syn*, 17,19-*syn*, 21,23-*anti*, and 25,27-*syn* 1,3-diols. Triacetonide **9a** was further treated with either (*S*)-(+)-MTPA-Cl or (*R*)-(−)-MTPA- Cl to produce (*S*)-(+)- and (*R*)-(−)-MTPA esters **9S** and 9**R**, respectively. The analysis of Δ*δ*_*S*-*R*_ values allowed for the assignment of 11*R* (Fig. 4F, Table S6). Unfortunately, the results from Δ*δ*_*S*-*R*_ values failed to assignment the configuration at C-13, probably because the two groups of Mosher agent were too close. In the NOESY spectrum of **9a** (Fig. 4D), strong NOE correlations of H-10 (*δ* 3.57) with H-15 (*δ* 3.38), H-13 (*δ* 3.90) with H-11 (*δ* 3.31) and Me-14 (*δ* 0.78), and no NOE between H-10 and H-11, as well as a large coupling constant (^3^ *J*_H10−11_, 8.8 Hz) (Table S5) suggested an *α*-orientation for H-10 and H-15 and an *β*-orientation for H-11, H-13, and Me, -14. Therefore, the configurations of C-10, C-13, C-14, and C-15 were assigned as 10*S*, 13*S*, 14*R*, and 15*R*. Based on the foregoing evidences, the structure of **9** was determined as novel polyene-polyol macrolide, named as flavofungin IX.

Compound **3** gave a [M + Na]^+^ ion at *m*/*z* 707.3972, correspondence to a molecular formula C_36_H_60_O_12_. The general features of the NMR spectra of **3** were very similar to those of **1**, indicating they were structurally related compounds. The main differences were found at the positions of C-10 and C-11, where two olefinic methines of **1** were replaced by two oxymethines in **3**. The UV absorption maximum at 330 nm for **3** (365 nm for **1**) also supported a smaller conjugated system in **3**. The configurational assignments of 11*S*, 13*S*, 14*R*, 15*R*, 17*R*, 19*R*, 21*S*, 23*S*, 25*R*, 27*R*, 30*S*, and 31*S* in **3** were achieved using the same protocol as for compounds **4**–**9**, including ^13^C Universal NMR database (Fig. 2), ^13^C NMR chemical shifts (Table S3) of the acetonide methyl and acetal carbons in tetraacetonide derivative **3a**, NOE information (Fig. 4A), and the same biosynthetic pathway these macrolides might have. The orientations of αH-10 and βH-11 in **3** were indicated by NOE correlations of H-11 (*δ* 3.70) with H-9 (*δ* 5.71) and H-13 (*δ* 4.20), and no NOE between H-11 and H-10 (*δ* 3.83) in **3a**, as well as the large coupling constant (*δ* 3.79, t, 7.3 Hz, H-10) for ^3^ *J*_H9−10_ and ^3^ *J*_H10−11_ (Table 2) in **3**, suggesting 10*R* conformation for C-10. Therefore, the structure of **3** was assigned as a new polyene-polyol macrolide, named as flavofungin III.

All isolated compounds (**1**–**10**) were evaluated by sulforhodamine B (SRB) assay for their activity in inhibiting proliferation of glioma cells U251, U87MG, SHG44, and C6. Doxorubicin (DOX) was used as a positive control. The results (Supplementary Information, Table S2) indicated that flavofungins I (**1**) and II (**2**) and spectinabilin (**10**) showed different sensitivities to glioma cells. Flavofungin II (**2**) and spectinabilin (**10**) showed moderate activity against the proliferation of four tested glioma cell lines with IC_50_ values of 15.67–56.67 μM for **2** and 10.86–42.75 μM for **10**. The anti-glioma property of these two compounds has not been reported previously. Flavofungin I (**1**) showed weak activity with an IC_50_ value of 45.91–87.45 μM. Unfortunately, new flavofungins III–IX (**3**–**9**) were inactive. Therefore, flavofungin II (**2**) and spectinabilin (**10**) are the main components responsible for the anti-glioma activity in the crude extract. The cytotoxicity (CC_50_) of the two active compounds **2** and **10** towards normal human astrocyte (HA) and human foreskin fibroblast (HFF-1) cells were also assayed. The results (Table S2) indicated both compounds showed a higher selectivity index (CC_50_/IC_50_) for HA with 4.6–16.5 for **2** and 3.4–13.3 for **10**, when compared to the selectivity index for HFF-1 with 1.8–6.4 for **2** and 1.3–5.1 for **10**.

Compounds (**1**–**10**) were also tested for their antimicrobial activity against methicillin-resistant *Staphylococcus aureus* ATCC 43300, *Escherichia coli* ATCC 25922, and *Candida albicans* by micro broth dilution method. Flavofungins I (**1**) and II (**2**) and spectinabilin (**10**) showed activity in suppressing the growth of *C*. *albicans* with MIC value of 12.5 μg/mL. The control drug amphotericin B had antifungal activity with MIC value of 3.125 μg/mL.

## Methods

### General Experimental Procedures

This section was supplied as online Supplementary Information.

### Strain Isolation

Strains were isolated from a sample of mangrove soils, which were collected from the Qi’ao Mangrove Forest in Zhuhai City, Guangdong, China, in October, 2013. Briefly, the dried soils (1.0 g) were diluted with sterile water to make 0.001 g/mL suspension and 200 μL suspension were covered on the surface of Gause’s solid medium and then incubated at 28 °C for 10 days. The single colony was picked with sterile needles and transferred to a Gause’s-agar plate. After another 10 days of growth at 28 °C, the single colony that grew well was transferred onto Gause’s agar slants and stored at 4 °C until use. A total of ten strains were obtained from this mangrove sample in this study.

### Preparation of Crude Extract for Bioactive Assay

Each isolated strain was cultured in Gause’s solid medium with a small Petri dish (90 mm, 25 mL medium) at room temperature for 10 days. The solid culture was cut into small pieces (about 0.6 × 0.6 cm) and then percolated with MeOH three times (50 mL, each). The combined MeOH solution was dried under reduced pressure to give crude extract. This crude extract was redissolved in DMSO to prepare crude sample (1.0 mg/mL). Sulforhodamine B (SRB) assay was used to evaluate the activity of the crude sample against the proliferation of glioma U87MG and U251 cells. The crude extract prepared from the culture of strain ZQ4BG was found to be the most active and the strain ZQ4BG was thus selected for this study.

### Taxonomic identity of *Streptomyces* sp. ZQ4BG

The 16S rDNA sequence analysis of strain ZQ4BG was conducted by Majorbio (Shanghai, China) and its DNA sequence using BLAST (nucleotide sequence comparison) was compared to the GenBank database. The 16S rDNA sequence of strain ZQ4BG has been deposited in GenBank (accession number: KX601187). The voucher strain of *Streptomyces sp*. ZQ4BG was preserved at the Laboratory of Institute of Marine Biology, Ocean College, Zhejiang University, China.

### Large culture of strain *Streptomyces* sp. ZQ4BG

Colonies of the strain ZQ4BG growing on Gause’s-agar slants were inoculated into a 500 mL Erlenmeyer flask containing 200 mL of Gause’s liquid medium and then incubated at 28 °C for 5 days on a rotary shaker (180 rpm) to produce seed broth. The seed broth (5 mL) was inoculated into a 500 mL Erlenmeyer flask, which contains 200 mL of Gause’s liquid medium. All flasks were incubated at 28 °C for 12 days on a rotary shaker (180 rpm). A total of 60 L cultures was prepared for this study.

### Extraction and Isolation of compounds 1–10

The 60 L cultures were filtered to give filtrate and mycelia. The filtrate was applied to a column of Diaion HP-20 eluting with water and then 100% MeOH. The MeOH elution was concentrated *in vacuo* to give Part A. The mycelia were extracted with MeOH five times and the combined MeOH solution was concentrated *in vacuo* to afford Part B. The combination of Part A and Part B was suspended in water and then partitioned by EtOAc three times to afford a crude extract (30.0 g) after removal of the solvent EtOAc. This crude extract was fractionated on a brown column of ODS eluting successively with 70% MeOH, 80% MeOH, 90% MeOH, and 100% MeOH to give fractions A–J based on the results of TLC and LC/MS analyses. Fraction H was separated by HPLC using an Agilent Zorbax SB-C_18_ column (mobile phase: MeOH/H_2_O, 85/15; flow rate: 1.0 mL/min) to give compounds **1** (33 mg, t_R_ 24.3 min) and **2** (31 mg, t_R_ 28.8 min). By using the same column and flow rate for **1** and **2** but different mobile phase, **3** (10 mg, t_R_ 70.1 min, CH_3_CN/H_2_O, 30/70) and **8** (18 mg, t_R_ 84.0 min, CH_3_CN/H_2_O, 30/70) from fraction C, **4** (80 mg, t_R_ 70.1 min, MeOH/H_2_O, 65/35) and **7** (6 mg, *t*_*R*_ 67.1 min, MeOH/H_2_O, 65/35) from fraction D, and **10** (200 mg, t_R_ 60.5 min, MeOH/H_2_O, 85/15) from fraction I, were obtained. While fraction B was separated by a preparative HPLC using a Sepax Amethyst C_18_-H column (mobile phase: CH_3_CN/H_2_O, 30/70; flow rate: 10.0 mL/min) to give **9** (57 mg, t_R_ 37.5 min) and an impure compound, which was further purified by HPLC using an Agilent Zorbax SB-C_18_ column (mobile phase: CH_3_CN/H_2_O, 30/70) to furnish **6** (27 mg, t_R_ 53.4 min). Similarly, fraction E was separated by a preparative HPLC using the same column and flow rate for **9** to afford **5** (30 mg, t_R_ 57.5 min MeOH/H_2_O, 65/35).

*Flavofungin I* (***1***): Yellow amorphous powder; molecular formula C_36_H_58_O_10_; UV (MeOH) λ_max_ (log ε) 365 (4.66) nm; ^13^C NMR data, see Table 1, ^1^H NMR data, see Table 2; HRESIMS *m*/*z* [M + Na]^+^ 673.3919 (calcd for C_36_H_58_NaO_10_, 673.3928).

*Flavofungin II* (***2***): Yellow amorphous powder; molecular formula C_37_H_60_O_10_; UV (MeOH) λ_max_ (log ε) 364 (4.37) nm; ^13^C NMR data, see Table 1, ^1^H NMR data, see Table 2; HRESIMS *m*/*z* [M + Na]^+^ 687.4079 (calcd for C_37_H_60_NaO_10_, 687.4084).

*Flavofungin III* (***3***): Yellow amorphous powder; molecular formula C_36_H_60_O_12_; UV (MeOH) λ_max_ (log ε) 330 (4.76) nm; IR (KBr)*ν*_max_ 3384, 2925, 1690, 1620, 1596, 1429, 1355, 1309, 1123, 1011 cm^−1^; ^13^C NMR data, see Table 1, ^1^H NMR data, see Table 2; HRESIMS *m*/*z* [M + Na]^+^ 707.3972 (calcd for C_36_H_60_NaO_12_, 707.3982).

*Flavofungin IV* (***4***): Yellow amorphous powder; molecular formula C_36_H_58_O_11_; UV (MeOH) λ_max_ (log ε) 330 (4.59) nm; IR (KBr)*ν*_max_ 3385, 2937, 1704, 1621, 1596, 1417, 1370, 1296, 1125, 1009 cm^−1^; ^13^C NMR data, see Table 1, ^1^H NMR data, see Table 2; HRESIMS *m*/*z* [M + Na]^+^ 689.3870 (calcd for C_36_H_58_NaO_11_, 689.3877).

*Flavofungin V* (***5***): Yellow amorphous powder; molecular formula C_37_H_60_O_11_; UV (MeOH) λ_max_ (log ε) 331 (4.28) nm; IR (KBr)*ν*_max_ 3383, 2934, 1703, 1620, 1596, 1425, 1369, 1290, 1125, 1008 cm^−1^; ^13^C NMR data, see Table 1, ^1^H NMR data, see Table 3; HRESIMS *m*/*z* [M + Na]^+^ 703.4022 (calcd for C_37_H_60_NaO_11_, 703.4033).

*Flavofungin VI* (***6***): Yellow amorphous powder; molecular formula C_36_H_58_O_11_; UV (MeOH) λ_max_ (log ε) 335 (4.64) nm; IR (KBr)*ν*_max_ 3384, 2935, 1684, 1621, 1595, 1435, 1371, 1300, 1125, 1011 cm^−1^; ^13^C NMR data, see Table 1, ^1^H NMR data, see Table 3; HRESIMS *m*/*z* [M + Na]^+^ 689.3872 (calcd for C_36_H_58_NaO_11_, 689.3877).

*Flavofungin VII* (***7***): Yellow amorphous powder; molecular formula C_36_H_58_O_11_; UV (MeOH) λ_max_ (log ε) 335 (3.94) nm; IR (KBr)*ν*_max_ 3375, 2926, 1695, 1622, 1598, 1422, 1371, 1300, 1128, 1009 cm^−1^; ^13^C NMR data, see Table 1, ^1^H NMR data, see Table 3; HRESIMS *m*/*z* [M + Na]^+^ 689.3867 (calcd for C_36_H_58_NaO_11_, 689.3877).

*Flavofungin VIII* (***8***): Yellow amorphous powder; molecular formula C_35_H_56_O_11_; UV (MeOH) λ_max_ (log ε) 330 (5.02) nm; IR (KBr)*ν*_max_ 3374, 2936, 1704, 1620, 1596, 1423, 1371, 1296, 1124, 1009 cm^−1^; ^13^C NMR data, see Table 1, ^1^H NMR data, see Table 3; HRESIMS *m*/*z* [M + Na]^+^ 675.3714 (calcd for C_35_H_56_NaO_11_, 675.3720).

*Flavofungin IX* (***9***): Yellow amorphous powder; molecular formula C_36_H_58_O_11_; UV (MeOH) λ_max_ (log ε) 328 (4.45) nm; IR (KBr)*ν*_max_ 3385, 2937, 1699, 1622, 1597, 1410, 1319, 1297, 1125, 1011 cm^−1^; ^13^C NMR data, see Table 1, ^1^H NMR data, see Table 3; HRESIMS *m*/*z* [M + Na]^+^ 689.3864 (calcd for C_36_H_58_NaO_11_, 689.3877).

### Preparation of acetonide derivatives of flavofungins III–VI (3–6), VIII (8), and IX (9)

Pyridinium-*p*-toluenesulfonate (10 mg) and 2,2-dimethoxypropane (8.0 mL) were added to a solution of each flavofungin (**3**–**6**, **8**, **9**, 9–55 mg) in anhydrous CH_2_Cl_2_ (4.0 mL) and MeOH (400 μL) at room temperature. The mixture was stirred in dark under nitrogen for 12 h and then quenched with 5% aqueous NaHCO_3_. The reaction product was extracted with CH_2_Cl_2_ to give crude extract, which was fractionated by an ODS column eluting with 70% and 90% MeOH to give fractions 70 M and 90 M. Fraction 90 M was further purified by HPLC using an Agilent column (Zorbax SB-C_18_ 250 × 9.4 mm, 5μm) to afford pure acetonide derivatives **3a** (3.0 mg, t_R_ 72.5 min, CH_3_CN/H_2_O, 85/15), **4a** (15.0 mg, t_R_ 59.9 min, CH_3_CN/H_2_O, 80/20), **5a** (7.0 mg, t_R_ 36.6 min, MeOH/H_2_O, 92/8), **6a** (6.0 mg, t_R_ 35.4 min, CH_3_CN/H_2_O, 85/15), **8a** (3.0 mg, t_R_ 57.4 min, CH_3_CN/H_2_O, 75/25), **9a** (9.0 mg, t_R_ 38.5 min, MeOH/H_2_O, 92/8), and **9b** (20.0 mg, t_R_ 32.7 min, MeOH/H_2_O, 100/0).

*Compound* ***3a***: Yellow amorphous powder; molecular formula C_48_H_76_O_12_; ^13^C NMR data, see Table S3, ^1^H NMR data, see Table S4; HRESIMS *m*/*z* [M + Na]^+^ 867.5227 (calcd for C_48_H_76_NaO_12_, 867.5234).

*Compound* ***4a***: Yellow amorphous powder; molecular formula C_45_H_70_O_11_; ^13^C NMR data, see Table S3, ^1^H NMR data, see Table S4; HRESIMS *m*/*z* [M + Na]^+^ 809.4796 (calcd for C_45_H_70_NaO_11_, 809.4816).

*Compound* ***5a***: Yellow amorphous powder; molecular formula C_46_H_72_O_11_; ^13^C NMR data, see Table S3, ^1^H NMR data, see Table S4; HRESIMS *m*/*z* [M + Na]^+^ 823.4972 (calcd for C_46_H_72_NaO_12_, 823.4972).

*Compound* ***6a***: Yellow amorphous powder; molecular formula C_45_H_70_O_11_; ^13^C NMR data, see Table S3, ^1^H NMR data, see Table S5; HRESIMS *m*/*z* [M + Na]^+^ 809.4819 (calcd for C_45_H_70_NaO_11_, 809.4816).

*Compound* ***8a***: Yellow amorphous powder; molecular formula C_44_H_68_O_11_; ^13^C NMR data, see Table S3, ^1^H NMR data, see Table S5; HRESIMS *m*/*z* [M + Na]^+^ 795.4659 (calcd for C_44_H_68_NaO_11_, 795.4659).

*Compound* ***9a***: Yellow amorphous powder; molecular formula C_45_H_70_O_11_; ^13^C NMR data, see Table S3, ^1^H NMR data, see Table S5; HRESIMS *m*/*z* [M + Na]^+^ 809.4810 (calcd for C_45_H_70_NaO_11_, 809.4816).

*Compound* ***9b***: Yellow amorphous powder; molecular formula C_48_H_74_O_11_; ^13^C NMR data, see Table S3, ^1^H NMR data, see Table S5; HRESIMS *m*/*z* [M + Na]^+^ 849.5126 (calcd for C_48_H_74_NaO_11_, 849.5129).

### MTPA esterification of acetonide derivatives 4a and 9a

Dried acetonide derivative **4a** (4.0 mg) was divided into two equal portions. Anhydrous pyridine (1 mL) and 4-dimethylaminopyridine (2 mg) were added to each portion. The mixtures were stirred at room temperature for 10 min and then added either (*S*)-(+)-MTPA-Cl or (*R*)-(−)-MTPA-Cl (45 μL). The reaction mixtures were terminated with MeOH (1 mL) after 1 h and then dried under reduced pressure to give a residue. (*S*)-(+)-MTPA ester (**4S**, 1.2 mg, t_R_ 35.3 min) or (*R*)- (−)-MTPA ester (**4R**, 1.1 mg, t_R_ 35.9 min) was obtained from the residue by HPLC purification using an Agilent Zorbax SB-C_18_ column (250 × 9.4 mm, 5μm; mobile phase: CH_3_CN/H_2_O, 85/15; flow rate: 1.0 mL/min). In the same way, MTPA esterification of acetonide derivative **9a** (8.0 mg) gave (*S*)-(+)-MTPA ester (**9S**, 3.0 mg, t_R_ 26.2 min, MeOH/H_2_O, 90/10) and (*R*)-(−)-MTPA ester (**9R**, 3.0 mg, t_R_ 26.7 min).

(*S*)-(+)-*MTPA ester* ***4S***: Yellow amorphous powder; molecular formula C_56_H_72_F_6_O_15_; ^1^H NMR data, see Table S6; HRESIMS *m*/*z* [M + Na]^+^ 1121.4673 (calcd for C_56_H_72_F_6_NaO_15_, 1121.4673).

(*R*)-(−)-*MTPA ester* ***4R***: Yellow amorphous powder; molecular formula C_56_H_72_F_6_O_15_; ^1^H NMR data, see Table S6; HRESIMS *m*/*z* [M + Na]^+^ 1121.4653 (calcd for C_56_H_72_F_6_NaO_15_, 1121.4673).

(*S*)-(+)-*MTPA ester* ***9S***: Yellow amorphous powder; molecular formula C_56_H_72_F_6_O_15_; ^1^H NMR data, see Table S6; HRESIMS *m*/*z* [M + Na]^+^ 1121.4683 (calcd for C_56_H_72_F_6_NaO_15_, 1121.4673).

(*R*)-(−)-*MTPA ester* ***9R***: Yellow amorphous powder; molecular formula C_56_H_72_F_6_O_15_; ^1^H NMR data, see Table S6; HRESIMS *m*/*z* [M + Na]^+^ 1121.4677 (calcd for C_56_H_72_F_6_NaO_15_, 1121.4673).

### Anti-glioma bioactive assay

Sulforhodamine B (SRB) assay as described in previous publications^9, 10^ was used to evaluate the activity of all isolated compounds against the proliferation of glioma U87MG, U251, SHG44, and C6 cells as well as normal human astrocytes (HA) and human foreskin fibroblast (HFF-1). Doxorubicin (DOX, a chemotherapeutic drug)^41^ was used as a positive control (CON).

### Antimicrobial assay

The antimicrobial activity of the isolated compounds against growth of methicillin-resistant *Staphylococcus aureus* ATCC 43300, *Escherichia coli* ATCC 25922, and *Candida albicans* was assayed by the micro broth dilution method as described in previous study^13^. Gentamicin (an antibiotic against both Gram-positive and Gram-negative bacteria) and amphotericin B (an antifungal drug) were used as positive controls.

## Electronic supplementary material

Supplementary Information

## References

[1] Patil SA (2013). Novel approaches to glioma drug design and drug screening. Expert Opin. Drug Discov..

[2] Ostrom QT (2013). CBTRUS statistical report: primary brain and central nervous system tumors diagnosed in the United States in 2006–2010. Neuro. Oncol..

[3] Kamran N (2016). Recent advances and future of immunotherapy for glioblastoma. Expert Opin. Biol. Ther..

[4] Chamberlain MC (2010). Temozolomide: therapeutic limitations in the treatment of adult high-grade gliomas. Expert Rev. Neurother..

[5] Wu SL, Jiang LM (2012). Recent advances in mangrove actinomycetes. Curr. Biotechnol..

[6] Hong K (2013). Actinomycetes from mangrove and their secondary metabolites. Acta Microbiol. Sin..

[7] Xu DB, Ye WW, Han Y, Deng ZX, Hong K (2014). Natural products from mangrove actinomycetes. Mar. Drugs.

[8] Feling RH (2003). Salinosporamide A: a highly cytotoxic proteasome inhibitor from a novel microbial source, a marine bacterium of the new genus. Salinospora. Angew. Chem. Int. Ed. Engl..

[9] Xin W, Ye X, Yu S, Lian XY, Zhang Z (2012). New capoamycin-type antibiotics and polyene acids from marine *Streptomyces fradiae* PTZ0025. Mar. Drugs.

[10] Yu S, Ye X, Chen L, Lian XY, Zhang Z (2014). Polyoxygenated 24,28-epoxyergosterols inhibiting the proliferation of glioma cells from sea anemone *Anthopleura midori*. Steroids.

[11] Yu S (2015). Bioactive sulfated saponins from sea cucumber *Holothuria moebii*. Planta Med..

[12] Chen L (2015). Synthesis and bioactivity of tripolinolate A from *Tripolium vulgare* and its analogs. Bioorg. Med. Chem. Lett..

[13] Ye X (2016). A new curvularin glycoside and its cytotoxic and antibacterial analogues from marine actinomycete *Pseudonocardia* sp. HS7. Nat. Prod. Res..

[14] Liang Y (2016). Bioactive polycyclic quinones from marine *Streptomyces* sp. 182SMLY. Mar. Drugs.

[15] Zhang X, Ye X, Chai W, Lian XY, Zhang Z (2016). New metabolites and bioactive actinomycins from marine-derived *Streptomyces* sp. ZZ338. Mar. Drugs.

[16] Bognar R (1972). Flavofungin: a mixture of 13,15,17,19,21,23,25,27-octahydroxy-31-isopropyl-14,30-dimethyl-13,15,17,19,21,23,25,27-octahydroxy-14,30-dimethyl- and 31-sec-butyl-2,4,6,8,10,28-hentriacontahexaen-31-olide. J. Chem. Soc. Perkin. Trans. 1.

[17] Bognar R (1988). Structural studies on the major component of the desertomycin complex. Acta Chimica Hungarica.

[18] Hazen EL, Brown R (1950). Two antifungal agents produced by a soil actinomycete. Science.

[19] Nair MG (1989). Faeriefungin: a new broad-spectrum antibiotic from *Streptomyces griseus var*. *autotrophicus*. J. Nat. Prod..

[20] Nakagawa Y (2016). The structure of the bimolecular complex between amphotericin B and ergosterol in membranes is stabilized by face-to-face van der Waals interaction with their rigid cyclic cores. Biochemistry.

[21] Wasserman HH, Van Verth JE, McCaustland DJ, Borowitz IJ, Kamber B (1967). Mycoticins, polyene macrolides from *Streptomyces ruber*. J. Am. Chem. Soc..

[22] Kobinata K, Koshino H, Kudo T, Isono K, Osada H (1993). RK-397, a new oxo pentaene antibiotic. J. Antibiot..

[23] Koshino H, Kobinata K, Isono K, Osada H (1993). Structure of RK-397, a new oxo pentaene antibiotic. J. Antibiot..

[24] Mitton-Fry MJ, Cullen AJ, Sammakia T (2007). The total synthesis of the oxopolyene macrolide RK-397. Angew. Chem. Int. Ed. Engl..

[25] Brautaset T (2011). New nystatin-related antifungal polyene macrolides with altered polyol region generated via biosynthetic engineering of *Streptomyces noursei*. Appl. Environ. Microb..

[26] Che Q (2015). Genome scanning inspired isolation of reedsmycins A-F, polyene-polyol macrolides from *Streptomyces* sp. CHQ-64. RSC Adv..

[27] Kwon HC, Kauffman CA, Jensen PR, Fenical W (2009). Marinisporolides, polyene-polyol macrolides from a marine actinomycete of the new genus *Marinispora*. J. Org. Chem..

[28] Kim D-G (2012). Bahamaolides A and B, antifungal polyene polyol macrolides from the marine actinomycete *Streptomyces* sp. J. Nat. Prod..

[29] Wiley-VCH Verlag GmbH & Co. KGaA. AntiBase 2011.

[30] Schreiber SL, Goulet MT, Sammakia T (1987). Stereochemical studies of the skipped-polyol polyene macrolide class: NMR studies of a tetraformylal derivative of mycoticin A and B. Tetrahedron Lett..

[31] Szilagyi L, Sandor P (1990). Complete assignments of the proton and carbon-13 NMR spectra of the macrolide antibiotic flavofungin; intramolecular hydrogen bonding and conformation. Magn. Reson. Chem..

[32] Kakinuma K, Hanson CA, Rinehart KL (1976). Spectinabilin, a new nitro-containing metabolite isolated from *Streptomyces spectabilis*. Tetrahedron.

[33] Jacobsen MF, Moses JE, Adlington RM, Baldwin JE (2005). The total synthesis of spectinabilin and its biomimetic conversion to SNF4435C and SNF4435D. Org. Lett..

[34] Kawamura T (2010). Isolation and structure elucidation of a novel androgen antagonist, arabilin, produced by *Streptomyces* sp. MK756-CF1. J. Antibiot..

[35] Fraga BM, Hernândez MG, Gonzâlez P, Chamy MC, Garbarino JA (2000). The biotransformation of 18-hydroxy-9-epi-ent-pimara-7,15-diene by *Gibberella fujikuroi*. Phytochemistry.

[36] Fraga BM, Alvarez L, Suârez S (2003). Biotransformation of the diterpenes epicandicandiol and candicandiol by *Mucor plumbeus*. J. Nat. Prod..

[37] Kobayashi Y, Tan CH, Kishi Y (2000). Stereochemical assignment of the C_21_-C_38_ portion of the desertomycin/oasomycin class of natural products by using universal NMR databases: prediction. Angew. Chem. Int. Ed..

[38] Kobayashi Y, Tan CH, Kishi Y (2000). Toward creation of a universal NMR database for stereochemical assignment: the case of 1,3,5-trisubstituted acyclic systems. Helv. Chin. Acta.

[39] Kobayashi Y, Tan CH, Kishi Y (2001). Toward creation of a universal NMR database for stereochemical assignment: complete structure of the desertomycin/oasomycin class of natural products. J. Am. Chem. Soc..

[40] Rychnovsky SD, Richardson TI, Rogers BN (1997). Two-dimensional NMR analysis of acetonide derivatives in stereochemical assignment of polyol chains: the absolute configurations of dermostatins A and B. J. Org. Chem..

[41] Tacara O, Sriamornsak P, Dass CR (2013). Doxorubicin: an update on anticancer molecular action, toxicity and novel drug delivery systems. J. Pharm. Pharmacol..

