# Supplementary material for: Rare Polyene-polyol Macrolides from Mangrove-derived Streptomyces sp. ZQ4BG
Source: Sci Rep. 2017 May 10;7:1703. doi: 10.1038/s41598-017-01912-z (PMC5431850; doi:10.1038/s41598-017-01912-z)
Supplement: Supplementary file 1 — Supplementary Information [file 41598_2017_1912_MOESM1_ESM.doc]

**Supplementary Information for**

**Rare Polyene-polyol Macrolides from Marine Actinomycete *Streptomyces* sp. ZQ4BG**

Wenling Wang^1^, Tengfei Song^1^, Weiyun Chai^1^, Lu Chen^1^, Lei Chen^1^, Xiao-Yuan Lian^2^,*, Zhizhen Zhang^1^,*

^1^ Ocean College, Zhoushan Campus, Zhejiang University, Zhoushan 316021, China,

^2^ College of Pharmaceutical Sciences, Zhejiang University, Hangzhou 310058, China

*****Correspondence and requests for materials should be addressed to X.-Y. L (email: [xylian@zju.edu.cn](mailto:xylian@zju.edu.cn)) or Z. Z. (email: zzhang88@zju.edu.cn)

**Content**

Figure S_1_. 16S rDNA sequence of *Streptomyces* sp. ZQ4BG…………………..….….page 5

Table S_1_. Sequences producing significant alignments………………………………..page 5

Figure S_2_. Plausible mechanism for the formation of flavofungins III−IX (**3**−**9**) from flavofungins I (**1**) and II (**2**)………………………………………………………........page 6

Table S_2_. Antiproliferative and antifungal activities of **1**, **2**, and **10**…..……………….page 6

Table S_3_. ^13^C-NMR data of acetonide derivatives.……….……....……. .……..…...….page 7

Table S_4_. ^1^H-NMR data of acetonide derivatives………....……..….……....……. ..….page 8

Table S_5_. ^1^H-NMR data of acetonide derivatives…………………..…….. ...……...….page 9

Table S_6_. ^1^H-NMR data of compounds **4S**, **4R**, **9S**, and **9R**.…….……..…...……...….page 10

General Experimental Procedures…………………………………..…………………page 11

Figure S_3_. ^1^H-NMR spectrum of flavofungin I (**1**)……..…….…………………….….page 12

Figure S_4_. ^13^C-NMR spectrum of flavofungin I (**1**)….…..….……….……………..….page 12

Figure S_5_. ^1^H-^1^H COSY spectrum of flavofungin I (**1**)…...………….…………….….page 13

Figure S_6_. HSQC spectrum of flavofungin I (**1**)….……...….…….….…………….….page 13

Figure S_7_. HMBC spectrum of flavofungin I (**1**)………..…...………….….………….page 14

Figure S_8_. HRESIMS of flavofungin I (**1**)…………….…….……….……..………….page 14

Figure S_9_. ^1^H-NMR spectrum of flavofungin II (**2**)…….………..…………………….page 15

Figure S_10_. ^13^C-NMR spectrum of flavofungin II (**2**)………….…..……….………….page 15

Figure S_11_. ^1^H-^1^H COSY spectrum of flavofungin II (**2**)………….….………….…….page 16

Figure S_12_. HSQC spectrum of flavofungin II (**2**)…………….……………………….page 16

Figure S_13_. HRESIMS of flavofungin II (**2**)…………….………..…..……….……….page 17

Figure S_14_. ^1^H-NMR spectrum of flavofungin III (**3**)…………….…………...……….page 17

Figure S_15_. ^13^C-NMR spectrum of flavofungin III (**3**)…….………..………….…...….page 18

Figure S_16_. ^1^H-^1^H COSY spectrum of flavofungin III (**3**)………….………………….page 18

Figure S_17_. HSQC spectrum of flavofungin III (**3**)…………….…………….……..….page 19

Figure S_18_. HMBC spectrum of flavofungin III (**3**)………….………….………….….page 19

Figure S_19_. HRESIMS of flavofungin III (**3**)…………….……………...………… ….page 20

Figure S_20_. ^1^H-NMR spectrum of compound **3a**…………………...………………….page 20

Figure S_21_. HSQC spectrum of compound **3a**………………...……………………….page 21

Figure S_22_. ^1^H-^1^H COSY spectrum of compound **3a**…………….…………………....page 21

Figures S_23-25_. NOESY spectra of compound **3a**……………..….…….…………...….page 22

Figure S_26_. HRESIMS of compound **3a**……………….……………...……………….page 23

Figure S_27_. ^1^H-NMR spectrum of flavofungin IV (**4**)…...…….……..…....……..…….page 24

Figure S_28_. ^13^C-NMR spectrum of flavofungin IV (**4**)……...….………………..…….page 24

Figure S_29_. ^1^H-^1^H COSY spectrum of flavofungin IV (**4**)………….………………….page 25

Figure S_30_. HSQC spectrum of flavofungin IV (**4**)………….…….…………….…….page 25

Figure S_31_. HMBC spectrum of flavofungin IV (**4**)………….………...…………..….page 26

Figure S_32_. HRESIMS of flavofungin IV (**4**)……….……………..…………..……….page 26

Figure S_33_. ^1^H-NMR spectrum of compound **4a**…………....………………...……….page 27

Figure S_34_. ^13^C-NMR spectrum of compound **4a**…………….……..………...........….page 27

Figure S_35_. HSQC spectrum of compound **4a**……………….………...………...…….page 28

Figure S_36_. HMBC spectrum of compound **4a**……………….………….…………….page 28

Figures S_37-39_. NOESY spectra of compound **4a**………………………………..……..page 29

Figure S_40_. HRESIMS of compound **4a**………………………….……………...…….page 30

Figure S_41_. ^1^H-NMR spectrum of compound **4R**…………..………………………….page 31

Figure S_42_. HSQC spectrum of compound **4R**…………………………….…..………page 31

Figure S_43_. ^1^H-^1^H COSY spectrum of compound **4R**………………………....………page 32

Figure S_44_. HRESIMS of compound **4R**……………………………………...….……page 32

Figure S_45_. ^1^H-NMR spectrum of compound **4S**………..…….……….............………page 33

Figure S_46_. HSQC spectrum of compound **4S**…………………………………………page 33

Figure S_47_. ^1^H-^1^H COSY spectrum of compound **4S**……….……………..……..……page 34

Figure S_48_. HRESIMS of compound **4S**……………………………..………...………page 34

Figure S_49_. ^1^H-NMR spectrum of flavofungin V (**5**)………….…………...….……….page 35

Figure S_50_. ^13^C-NMR spectrum of flavofungin V (**5**)……..……………..............…….page 35

Figure S_51_. ^1^H-^1^H COSY spectrum of flavofungin V (**5**)……...……….………...…….page 36

Figure S_52_. HSQC spectrum of flavofungin V (**5**)…….….…………………..….…….page 36

Figure S_53_. HMBC spectrum of flavofungin V (**5**)………….…………………...…….page 37

Figure S_54_. HRESIMS of flavofungin V (**5**)………………………..……………...…..page 37

Figure S_55_. ^1^H-NMR spectrum of compound **5a**………………….…..……………….page 38

Figure S_56_. ^13^C-NMR spectrum of compound **5a**……………………….……………..page 38

Figure S_57_. HSQC spectrum of compound **5a**…………….……….....………….…….page 39

Figure S_58_. ^1^H-^1^HCOSY spectrum of compound **5a**………………………….……….page 39

Figure S_59_. NOESY spectrum of compound **5a**…………….…………….……...…….page 40

Figure S_60_. HRESIMS of compound **5a**……………….……………………...……….page 40

Figure S_61_. ^1^H-NMR spectrum of flavofungin VI (**6**)……..………….……….……….page 41

Figure S_62_. ^13^C-NMR spectrum of flavofungin VI (**6**)…….……………………….….page 41

Figure S_63_. ^1^H-^1^H COSY spectrum of flavofungin VI (**6**)…………………….……….page 42

Figure S_64_. HSQC spectrum of flavofungin VI (**6**)……….…………………..…….….page 42

Figure S_65_. HMBC spectrum of flavofungin VI (**6**)………….…………………..…….page 43

Figure S_66_. HRESIMS of flavofungin VI (**6**)………….……………..………….….….page 43

Figure S_67_. ^1^H-NMR spectrum of compound **6a**…………….………..………....…….page 44

Figure S_68_. ^13^C-NMR spectrum of compound **6a**…………….……………….……….page 44

Figure S_69_. HSQC spectrum of compound **6a**……………….……………….…….….page 45

Figures S_70-_S_72_. ^1^H-^1^HCOSY spectra of compound **6a**……………………….….…….page 45

Figures S_73-_S_76_. NOESY spectra of compound **6a**…………….…..……..…….…...….page 47

Figure S_77_. HRESIMS of compound **6a**………………………….…...………….……page 49

Figure S_78_. ^1^H-NMR spectrum of flavofungin VII (**7**)…….……………….……….....page 49

Figure S_79_. ^13^C-NMR spectrum of flavofungin VII (**7**)….……………….….…..…….page 50

Figure S_80_. ^1^H-^1^H COSY spectrum of flavofungin VII (**7**)…….……….….………….page 50

Figure S_81_. HSQC spectrum of flavofungin VII (**7**)………….…………..…………....page 51

Figures S_82-_S_84_. HMBC spectra of flavofungin VII (**7**)………….……..……………...page 51

Figure S_85_. NOESY spectrum of flavofungin VII (**7**)………………...….…………….page 53

Figure S_86_. HRESIMS of flavofungin VII (**7**)……….…….……..……………...…….page 53

Figure S_87_. ^1^H-NMR spectrum of flavofungin VIII (**8**)………….…………….…...….page 54

Figure S_88_. ^13^C-NMR spectrum of flavofungin VIII (**8**)……….…………...………….page 54

Figure S_89_. ^1^H-^1^H COSY spectrum of flavofungin VIII (**8**)………….………….…….page 55

Figure S_90_. HSQC spectrum of flavofungin VIII (**8**)………….…………….……...….page 55

Figure S_91_. HMBC spectrum of flavofungin VIII (**8**)………….……….………..…….page 56

Figure S_92_. HRESIMS of flavofungin VIII (**8**)……….….………..….…………….….page 56

Figure S_93_. ^1^H-NMR spectrum of compound **8a**….…………..…………..…….…......page 57

Figures S_94_-S_97_. HSQC spectra of compound **8a**…………….….…..….……………...page 57

Figure S_98_. ^1^H-^1^HCOSY spectrum of compound **8a**….…….…………..………….…..page 59

Figure S_99_. HRESIMS of compound **8a**……….……………….….…………….…….page 60

Figure S_100_. ^1^H-NMR spectrum of flavofungin IX (**9**)…….…….………..………..….page 60

Figure S_101_. ^13^C-NMR spectrum of flavofungin IX (**9**)…………..….….……....….….page 61

Figure S_102_. ^1^H-^1^H COSY spectrum of flavofungin IX (**9**)………….…..…….…....….page 61

Figure S_103_. HSQC spectrum of flavofungin IX (**9**)………….…….……….……...….page 62

Figures S_104_-S_106_. HMBC spectra of flavofungin IX (**9**)….……….………….……….page 62

Figure S_107_. HRESIMS of flavofungin IX (**9**) ….………………..….……….…….….page 64

Figure S_108_. ^1^H-NMR spectrum of compound **9a**……………...……….…….….…….page 64

Figure S_109_. ^13^C-NMR spectrum of compound **9a**………….….………..……..…...….page 65

Figure S_110_. HSQC spectrum of compound **9a**……………….….……...……….…….page 65

Figure S_111_. ^1^H-^1^H COSY spectrum of compound **9a**……..……………...…..…….….page 66

Figures S_112−_S_115_. NOESY spectra of compound **9a**…………….…….…….…...…….page 66

Figure S_116_. HRESIMS of compound **9a**…………………….…….………….……….page 68

Figure S_117_. ^1^H-NMR spectrum of compound **9b**….…………….…….……..…….….page 69

Figures S_118-120_. ^13^C-NMR spectra of compound **9b**….…………..………….……….page 69

Figure S_121_. HSQC spectrum of compound **9b**….……….….………..…….………….page 71

Figure S_122_. ^1^H-^1^H COSY spectrum of compound **9b**….…….………..…………...….page 71

Figures S_123_-S_126_. NOESY spectra of compound **9b**….………………………….…….page 72

Figure S_127_. HRESIMS of compound **9b**….……………………..…………….…...….page 74

Figure S_128_. ^1^H-NMR spectrum of compound **9R**…………….…….………...……….page 74

Figure S_129_. HSQC spectrum of compound **9R**….………….…………..….………….page 75

Figure S_130_. ^1^H-^1^H COSY spectrum of compound **9R**….………….……..………...….page 75

Figure S_131_. HRESIMS of compound **9R**….……………………………….……....….page 76

Figure S_132_. ^1^H-NMR spectrum of compound **9S**….……...….……………….……….page 76

Figure S_133_. HSQC spectrum of compound **9S**….………….……………....………….page 77

Figures S_134_-S_135_. ^1^H-^1^H COSY spectra of compound **9S**….………….…………...….page 77

Figure S_136_. HRESIMS of compound **9S**….……...…………….….…..…………...….page 78

Figure S_1_. 16S rDNA sequence of *Streptomyces* sp. ZQ4BG

ACCGCTAAATGCAGTCGAACGATGAAGCCCTTCGGGGTGGATTAGTGGCGAACGGGTGAGTAACACGTGGGCAATCTGCCCTGCACTCTGGGACAAGCCCTGGAAACGGGGTCTAATACCGGATAACACCCCCTCTCGCATGGGAGGGGGTTGAAAGCTCCGGCGGTGCAGGATGAGCCCGCGGCCTATCAGCTTGTTGGTGAGGTAGTGGCTCACCAAGGCGACGACGGGTAGCCGGCCTGAGAGGGCGACCGGCCACACTGGGACTGAGACACGGCCCAGACTCCTACGGGAGGCAGCAGTGGGGAATATTGCACAATGGGCGAAAGCCTGATGCAGCGACGCCGCGTGAGGGATGACGGCCTTCGGGTTGTAAACCTCTTTCAGCAGGGAAGAAGCGAAAGTGACGGTACCTGCAGAAGAAGCGCCGGCTAACTACGTGCCAGCAGCCGCGGTAATACGTAGGGCGCAAGCGTTGTCCCGGAATTATTGGGCGTAAAGAGCTCGTAGGCGGCTTGTCACGTCGGTTGTGAAAGCCCGGGGCTTAACCCCGGGTCTGCAGTCGATACGGGCAGGCTAGAGTTCGGTAGGGGAGATCGGAATTCCTGGTGTAGCGGTGAAATGCGCAGATATCAGGAGGAACACCGGTGGCGAAGGCGGATCTCTGGGCCGATACTGACGCTGAGGAGCGAAAGCGTGGGGAGCGAACAGGATTAGATACCCTGGTAGTCCACGCCGTAAACGGTGGGCACTAGGTGTGGGCAACATTCCACGTTGTCCGTGCCGCAGCTAACGCATTAAGTGCCCCGCCTGGGGAGTACGGCCGCAAGGCTAAAACTCAAAGGAATTGACGGGGGCCCGCACAAGCGGCGGAGCATGTGGCTTAATTCGACGCAACGCGAAGAACCTTACCAAGGCTTGACATACACCGGAAACGGCCAGAGATGGTCGCCCCCTTGTGGTCGGTGTACAGGTGGTGCATGGCTGTCGTCAGCTCGTGTCGTGAGATGTTGGGTTAAGTCCCGCAACGAGCGCAACCCTTGTCCCGTGTTGCCAGCAAGCCCTTCGGGGTGTTGGGGACTCACGGGAGACCGCCGGGGTCAACTCGGAGGAAGGTGGGGACGACGTCAAGTCATCATGCCCCTTATGTCTTGGGCTGCACACGTGCTACAATGGCCGGTACAATGAGCTGCGATACCGCGAGGTGGAGCGAATCTCAAAAAGCCGGTCTCAGTTCGGATTGGGGTCTGCAACTCGACCCCATGAAGTTGGAGTCGCTAGTAATCGCAGATCAGCATTGGTGCGGTGAATACGTTCCCGGGCCTTGTACACACCGCCCGTCACGTCACGAAAGTCGGTAACACCCGAAGCCGGTGGCCCAACCCCTTGTGGAGGGAGCTGAGA

Table S_1_. Sequences producing significant alignments

| Accession | Description | [Max score](http://blast.ncbi.nlm.nih.gov/Blast.cgi?CMD=Get&ALIGNMENTS=100&ALIGNMENT_VIEW=Pairwise&DATABASE_SORT=0&DESCRIPTIONS=100&DYNAMIC_FORMAT=on&FIRST_QUERY_NUM=0&FORMAT_OBJECT=Alignment&FORMAT_PAGE_TARGET=&FORMAT_TYPE=HTML&GET_SEQUENCE=yes&I_THRESH=&LINE_LENGTH=60&MASK_CHAR=2&MASK_COLOR=1&NUM_OVERVIEW=100&OLD_BLAST=false&PAGE=MegaBlast&QUERY_INDEX=0&QUERY_NUMBER=0&RESULTS_PAGE_TARGET=&RID=GBVWCUGG01R&SHOW_LINKOUT=yes&SHOW_OVERVIEW=yes&STEP_NUMBER=&OLD_VIEW=false&DISPLAY_SORT=1&HSP_SORT=1) | [Total score](http://blast.ncbi.nlm.nih.gov/Blast.cgi?CMD=Get&ALIGNMENTS=100&ALIGNMENT_VIEW=Pairwise&DATABASE_SORT=0&DESCRIPTIONS=100&DYNAMIC_FORMAT=on&FIRST_QUERY_NUM=0&FORMAT_OBJECT=Alignment&FORMAT_PAGE_TARGET=&FORMAT_TYPE=HTML&GET_SEQUENCE=yes&I_THRESH=&LINE_LENGTH=60&MASK_CHAR=2&MASK_COLOR=1&NUM_OVERVIEW=100&OLD_BLAST=false&PAGE=MegaBlast&QUERY_INDEX=0&QUERY_NUMBER=0&RESULTS_PAGE_TARGET=&RID=GBVWCUGG01R&SHOW_LINKOUT=yes&SHOW_OVERVIEW=yes&STEP_NUMBER=&OLD_VIEW=false&DISPLAY_SORT=2&HSP_SORT=1) | [Query cover](http://blast.ncbi.nlm.nih.gov/Blast.cgi?CMD=Get&ALIGNMENTS=100&ALIGNMENT_VIEW=Pairwise&DATABASE_SORT=0&DESCRIPTIONS=100&DYNAMIC_FORMAT=on&FIRST_QUERY_NUM=0&FORMAT_OBJECT=Alignment&FORMAT_PAGE_TARGET=&FORMAT_TYPE=HTML&GET_SEQUENCE=yes&I_THRESH=&LINE_LENGTH=60&MASK_CHAR=2&MASK_COLOR=1&NUM_OVERVIEW=100&OLD_BLAST=false&PAGE=MegaBlast&QUERY_INDEX=0&QUERY_NUMBER=0&RESULTS_PAGE_TARGET=&RID=GBVWCUGG01R&SHOW_LINKOUT=yes&SHOW_OVERVIEW=yes&STEP_NUMBER=&OLD_VIEW=false&DISPLAY_SORT=4&HSP_SORT=0) | [Iden](http://blast.ncbi.nlm.nih.gov/Blast.cgi?CMD=Get&ALIGNMENTS=100&ALIGNMENT_VIEW=Pairwise&DATABASE_SORT=0&DESCRIPTIONS=100&DYNAMIC_FORMAT=on&FIRST_QUERY_NUM=0&FORMAT_OBJECT=Alignment&FORMAT_PAGE_TARGET=&FORMAT_TYPE=HTML&GET_SEQUENCE=yes&I_THRESH=&LINE_LENGTH=60&MASK_CHAR=2&MASK_COLOR=1&NUM_OVERVIEW=100&OLD_BLAST=false&PAGE=MegaBlast&QUERY_INDEX=0&QUERY_NUMBER=0&RESULTS_PAGE_TARGET=&RID=GBVWCUGG01R&SHOW_LINKOUT=yes&SHOW_OVERVIEW=yes&STEP_NUMBER=&DISPLAY_SORT=3&HSP_SORT=3) |
| --- | --- | --- | --- | --- | --- |
| [GU350482.1](http://www.ncbi.nlm.nih.gov/nucleotide/284022420?report=genbank&log$=nucltop&blast_rank=1&RID=GBZ0206V01R) | *Streptomyces fulvissimus* strain MJM9627 | 2555 | 2555 | 99% | 99% |
| [LM999765.1](http://www.ncbi.nlm.nih.gov/nucleotide/673920936?report=genbank&log$=nucltop&blast_rank=2&RID=GBZ0206V01R) | *Streptomyces fulvissimus* strain DSM 40593T | 2545 | 2545 | 99% | 99% |
| [NR_041210.1](http://www.ncbi.nlm.nih.gov/nucleotide/343200523?report=genbank&log$=nucltop&blast_rank=3&RID=GBZ0206V01R) | *Streptomyces fulvissimus* strain NBRC 3717 | 2545 | 2545 | 99% | 99% |
| [NR_112408.1](http://www.ncbi.nlm.nih.gov/nucleotide/631251211?report=genbank&log$=nucltop&blast_rank=4&RID=GBZ0206V01R) | *Streptomyces fulvissimus* strain NBRC 13482 | 2545 | 2545 | 99% | 99% |
| [KJ751551.1](http://www.ncbi.nlm.nih.gov/nucleotide/667755360?report=genbank&log$=nucltop&blast_rank=5&RID=GBZ0206V01R) | *Streptomyces fulvissimus* strain CZB40 | 2527 | 2527 | 99% | 99% |
| [JN999903.1](http://www.ncbi.nlm.nih.gov/nucleotide/379975157?report=genbank&log$=nucltop&blast_rank=6&RID=GBZ0206V01R) | *Streptomyces flavofungini* strain NXPT4 | 2527 | 2527 | 99% | 99% |
| [NR_041125.1](http://www.ncbi.nlm.nih.gov/nucleotide/343200438?report=genbank&log$=nucltop&blast_rank=7&RID=GBZ0206V01R) | *Streptomyces flavofungini* strain NXPT4 | 2523 | 2523 | 99% | 99% |

Figure S_2_. Plausible mechanism for the formation of flavofungins III−IX (**3**−**9**) from flavofungins I (**1**) and II (**2**).

Table S_2_. Antiproliferative activity of **1**, **2**, and **10** (mean ± s.d., n = 4)

|  | **1** | **2** | **10** | DOX |
| --- | --- | --- | --- | --- |
| U251(IC_50_: μM) | 87.49 ± 0.04 | 19.45 ± 0.29 | 42.75 ± 0.03 | 9.61 ± 1.25 |
| U87MG (IC_50_: μM) | 45.91 ± 0.05 | 56.67 ± 0.08 | 29.36 ± 0.08 | 1.88 ± 0.37 |
| SHG44 (IC_50_: μM) | 57.19 ± 0.23 | 20.53 ± 0.08 | 10.86 ± 1.14 | 2.54 ± 0.23 |
| C6 (IC_50_: μM) | 79.86 ± 0.06 | 15.67 ± 0.12 | 12.86 ± 2.15 | 0.50 ± 0.10 |
| HA (CC_50_: μM) | NT | 259.13 ± 3.68 | 144.12 ± 3.12 | 8.70 ± 1.20 |
| Selectivity index  (CC_50_/IC_50_) | − | 13.3, 4.6, 12.6, 16.5 | 3.4, 4.9, 13.3, 11.2 | 0.9, 4.6, 3.4, 17.4 |
| HFF-1(CC_50_: μM) | NT | 100.31 ± 2.89 | 55.79 ± 2.13 | 0.27 ± 0.01 |
| Selectivity index  (CC_50_/IC_50_) | − | 5.2, 1.8, 4.9, 6.4 | 1.3, 1.9, 5.1, 4.3 | 0.03, 0.14, 0.11, 0.54 |

NT: no testing

Table S_3_. ^13^C-NMR data of acetonide derivatives (125 MHz, in DMSO-*d*_6_)

| **No.** | **3a***^#^* | **4a** | **5a** | **6a** | **8a***^#^* | **9a** | **9b*** |
| --- | --- | --- | --- | --- | --- | --- | --- |
| 1 | − | 166.3 | 165.9 | 165.6 | − | 166.2 | 167.0 |
| 2 | 120.5 | 120.9 | 120.6 | 120.8 | 120.3 | 120.3 | 121.4 |
| 3 | 144.5 | 144.7 | 144.3 | 143.6 | 144.3 | 144.9 | 144.8 |
| 4 | 129.9 | 130.2 | 129.8 | 129.6 | 129.7 | 129.4 | 130.0 |
| 5 | 140.8 | 141.2 | 140.8 | 140.7 | 140.7 | 141.1 | 140.9 |
| 6 | 131.6 | 132.3 | 131.9 | 131.3 | 131.6 | 131.0 | 131.5 |
| 7 | 136.8 | 136.5 | 136.2 | 136.6 | 136.3 | 137.1 | 136.8 |
| 8 | 131.5 | 130.8 | 130.4 | 130.8 | 129.9 | 130.2 | 129.3 |
| 9 | 135.9 | 136.1 | 136.0 | 134.2 | 136.8 | 137.1 | 136.9 |
| 10 | 74.7 | 86.4 | 86.4 | 81.9 | 85.7 | 87.6 | 86.8 |
| 11 | 72.3 | 74.9 | 74.7 | 72.1 | 74.9 | 71.8 | 76.1 |
| 12 | 25.6 | 40.5 | 40.3 | 41.3 | 39.3 | 40.3 | 28.8 |
| 13 | 68.1 | 79.4 | 79.1 | 78.0 | 74.5 | 67.1 | 72.1 |
| 14 | 43.2 | 42.3 | 41.9 | 43.8 | 40.5 | 44.1 | 47.0 |
| 15 | 67.2 | 69.1 | 68.8 | 67.1 | 63.9 | 81.5 | 81.7 |
| 16 | 42.0 | 40.8 | 40.7 | 39.0 | 42.7 | 39.6 | 39.6 |
| 17 | 66.4 | 67.0 | 66.7 | 66.8 | 66.4 | 65.6 | 66.8 |
| 18 | 36.5 | 37.3 | 37.1 | 37.0 | 36.5 | 34.9 | 35.9 |
| 19 | 65.8 | 64.8 | 64.5 | 65.0 | 65.3 | 64.1 | 65.1 |
| 20 | 42.0 | 42.3*^a^* | 42.1*^a^* | 42.0*^a^* | 41.6*^a^* | 42.3 | 43.8 |
| 21 | 62.7 | 61.9 | 61.6 | 61.9 | 61.9 | 61.1 | 62.0 |
| 22 | 38.2 | 38.5 | 38.3 | 38.0 | 38.1 | 38.8 | 39.3 |
| 23 | 62.4 | 62.3 | 62.0 | 62.4 | 62.1 | 61.5 | 62.9 |
| 24 | 42.0 | 42.4*^a^* | 42.2*^a^* | 42.1*^a^* | 42.0*^a^* | 43.5 | 43.3 |
| 25 | 65.2 | 64.7 | 64.4 | 64.9 | 64.8 | 63.5 | 64.3 |
| 26 | 36.8 | 37.9 | 37.6 | 37.3 | 37.3 | 38.7 | 39.3 |
| 27 | 68.5 | 68.1 | 67.7 | 67.8 | 67.6 | 66.8 | 68.3 |
| 28 | 130.5 | 131.1 | 130.7 | 130.6 | 131.0 | 130.8 | 131.2 |
| 29 | 132.5 | 132.3 | 131.9 | 131.0 | 131.3 | 130.4 | 131.9 |
| 30 | 35.8 | 35.8 | 35.4 | 35.9 | 35.6 | 35.4 | 36.7 |
| 31 | 80.0 | 80.7 | 78.6 | 79.5 | 80.0 | 79.8 | 80.6 |
| 32 | 28.9 | 29.1 | 35.0 | 28.8 | 28.5 | 28.9 | 29.5 |
| 33 | 18.6 | 18.8 | 14.5 | 19.0 | 18.6 | 18.6 | 19.4 |
| 34 | 19.4 | 19.8 | 25.0 | 19.4 | 19.0 | 19.6 | 19.7 |
| 35 | − | − | 10.5 | − | − | − | − |
| Me-14 | 10.5 | 14.4 | 14.3 | 10.2 | − | 12.5 | 15.7 |
| Me-30 | 12.0 | 11.7 | 11.2 | 12.0 | 11.6 | 11.0 | 11.9 |
| 36 | − | − | − | − | − | − | 98.5*^a^* |
| 37 | 19.5 | − | − | − | − | − | 27.3 |
| 38 | 29.6*^a^* | − | − | − | − | − | 32.2 |
| 39 | − | 98.0*^b^* | 97.6*^b^* | 97.6*^b^* | − | 97.4*^a^* | 98.6*^a^*. |
| 40 | 19.5 | 19.9 | 19.7 | 19.7 | 19.5 | 19.5 | 19.9 |
| 41 | 29.9*^a^* | 30.2*^c^* | 30.0*^c^* | 30.0*^c^* | 30.0 | 30.0*^b^* | 30.4*^b^* |
| 42 | − | 100.1 | 99.7 | 99.6 | − | 99.5 | 100.4 |
| 43 | 24.5 | 24.5*^d^* | 24.3*^d^* | 24.4*^d^* | 24.4 | 24.4*^c^* | 24.9*^c^* |
| 44 | 24.5 | 24.6*^d^* | 24.4*^d^* | 24.5*^d^* | 24.4 | 24.7*^c^* | 25.3*^c^* |
| 45 | − | 98.1*^b^* | 97.7*^b^* | 97.8*^b^* | − | 97.6*^a^* | 98.8*^a^* |
| 46 | 29.9*^a^* | 30.3*^c^* | 30.1*^c^* | 30.1*^c^* | 30.0 | 30.2*^b^* | 30.6*^b^* |
| 47 | 19.5 | 19.9 | 19.7 | 19.7 | 19.5 | 19.5 | 19.9 |

*^#^* Data were obtained from HSQC spectra; *Data were obtained in CDCl_3_-*d*; *^a^*^−^*^d^* Data with the same labels in each column may be interchanged.

Table S_4_. ^1^H-NMR data of acetonide derivatives (500 MHz, in DMSO-*d*_6_, *J* = Hz)

| No. | **3a** | **4a** | **5a** |
| --- | --- | --- | --- |
| 2 | 5.97, d (15.1) | 5.87, d (15.1) | 5.89, d (15.1) |
| 3 | 7.22, dd (15.1, 11.3) | 7.18, dd (15.1, 11.5) | 7.19, dd (15.1, 11.3) |
| 4 | 6.46, dd (14.9, 11.3) | 6.41, dd (14.7, 11.5) | 6.42, dd (14.8, 11.3) |
| 5 | 6.70, dd (14.9, 10.9) | 6.72, dd (14.7, 10.9) | 6.74, dd (14.8, 10.7) |
| 6 | 6.36, dd (15.1, 10.9) | 6.33, dd (14.7, 10.9) | 6.34, dd (14.8, 10.7) |
| 7 | 6.44, dd (15.1, 10.9) | 6.41, dd (14.7, 11.5) | 6.42, dd (14.8, 11.3) |
| 8 | 6.30, dd (15.1, 10.9) | 6.27, dd (15.0, 11.5) | 6.28, dd (15.0, 11.3) |
| 9 | 5.71, dd (15.1, 7.9) | 5.79, dd (15.0, 7.5) | 5.80, dd (15.0, 7.5) |
| 10 | 3.83, m | 4.03, dd (7.5, 2.9) | 4.04, dd (7.5, 2.7) |
| 11 | 3.70, m | 3.89, m | 3.89, m |
| 12 | 0.97, m; 1.16, m | 1.75, m; 1.86, m | 1.76, m; 1.86, m |
| 13 | 4.20, m | 3.93, m | 3.95, m |
| 14 | 1.55, m | 1.62, m | 1.63, m |
| 15 | 3.13, m | 3.70, m | 3.70, m |
| 16 | 1.40, m; 1.46, m | 1.40, m; 1.79, m | 1.40, m; 1.81, m |
| 17 | 4.02, m | 3.99, m | 4.01, m |
| 18 | 0.85, m; 1.65, m | 0.89, m; 1.35, m | 0.90, m; 1.36, m |
| 19 | 3.86, m | 3.86, m | 3.86, m |
| 20 | 1.40, m; 1.46, m | 1.29, m; 1.39, m | 1.29, m; 1.40, m |
| 21 | 3.82, m | 3.80, m | 3.81, m |
| 22 | 1.52, m | 1.32, m; 1.37, m | 1.33, m; 1.38, m |
| 23 | 3.72, m | 3.74, m | 3.75, m |
| 24 | 1.40, m; 1.46 | 1.29, m; 1.39 | 1.29, m; 1.40 |
| 25 | 3.84, m | 3.82, m | 3.83, m |
| 26 | 0.92, m; 1.37, m | 0.81, m; 1.25, m | 0.82, m; 1.25, m |
| 27 | 4.26, m | 4.24, m | 4.25, m |
| 28 | 5.37, dd (15.9, 5.3) | 5.34, dd (15.8, 4.8) | 5.37, dd (16.0, 4.8) |
| 29 | 5.46, dd (15.9, 6.3) | 5.44, dd (15.8, 5.8) | 5.46, dd (16.0, 5.5) |
| 30 | 2.55, m | 2.54, m | 2.55, m |
| 31 | 4.64, dd (9.8, 2.4) | 4.63, dd (9.7, 2.6) | 4.74, dd (10.1, 2.5) |
| 32 | 1.83, m | 1.81, m | 1.62, m |
| 33 | 0.90, d (6.7) | 0.89, d (6.8) | 0.88, d (6.9) |
| 34 | 0.79, d (6.7) | 0.78, d (6.8) | 0.99, m; 1.35, m |
| 35 | − | − | 0.81, t (7.5) |
| Me-14 | 0.70, d (6.9) | 0.79, d (6.5) | 0.80, d (6.9) |
| Me-30 | 0.97, d (6.9) | 0.96, d (7.0) | 0.97, d (6.8) |
| 37 | 1.38, s | − | − |
| 38 | 1.24, s | − | − |
| 40 | 1.35, s | 1.35, s | 1.37, s |
| 41 | 1.28, s | 1.22, s | 1.23*^a^*, s |
| 43 | 1.17, s | 1.25, s | 1.27, s |
| 44 | 1.20, s | 1.20, s | 1.22, s |
| 46 | 1.22, s | 1.22, s | 1.24*^a^*, s |
| 47 | 1.34, s | 1.31, s | 1.32, s |
| OH-10 | 4.88, d (4.2) | − | − |
| OH-11 | − | 5.17, br.s | 5.08, d (3.5) |
| OH-15 | 4.40, d (6.6) | 4.34, d (4.8) | 4.26, d (4.8) |

*^a^*,*^b^* Data with the same labels in each column may be interchanged.

Table S_5_. ^1^H-NMR data of acetonide derivatives (500 MHz, in DMSO-*d*_6_, *J* = Hz)

| No. | **6a** | **8a** | **9a** | **9b*** |
| --- | --- | --- | --- | --- |
| 2 | 5.79, d (15.1) | 5.92, d (15.0) | 5.87, d (15.1) | 5.80, d (15.1) |
| 3 | 7.18, dd (15.1, 11.3) | 7.21, dd (15.0, 11.2) | 7.21, dd (15.1, 11.4) | 7.23, dd (15.1, 11.3) |
| 4 | 6.37, dd (14.9, 11.3) | 6.44, dd (14.8, 11.2) | 6.44, dd (14.8, 11.4) | 6.30, dd (14.9, 11.3) |
| 5 | 6.78, dd (14.9, 11.2) | 6.73, dd (14.8, 10.4) | 6.71, dd (14.8, 11.0) | 6.50, dd (14.9, 10.8) |
| 6 | 6.28, dd (14.8, 11.2) | 6.38, dd (14.8, 10.4) | 6.30, dd (14.8, 11.0) | 6.22, dd (14.6, 10.8) |
| 7 | 6.50, dd (14.8, 11.0) | 6.42, dd (14.8, 10.7) | 6.44, dd (14.8, 11.4) | 6.34, dd (14.6, 10.5) |
| 8 | 6.28, dd (14.8, 11.0) | 6.31, dd (14.9, 10.7) | 6.17, dd (15.1, 11.4) | 6.25, dd (15.0, 10.5) |
| 9 | 5.93, dd (14.8, 5.7) | 5.84, dd (15.0, 6.6) | 5.81, dd (15.1, 6.6) | 5.79, dd (15.0, 6.6) |
| 10 | 4.30, dd (5.7, 3.9) | 4.14, dd (6.6, 2.3) | 3.57, dd (8.8, 6.6) | 4.20, t (6.6) |
| 11 | 4.20, m | 3.99, m | 3.31, m | 3.97, m |
| 12 | 1.66, m; 1.91, m | 1.68, m; 1.84, m | 1.73, m; 1.90, m | 2.12, m; 2.34, m |
| 13 | 3.80, m | 4.35, m | 3.90, m | 4.08, m |
| 14 | 1.62, m | 1.64, m | 1.73, m | 1.60, m |
| 15 | 3.85, m | 3.74, m | 3.38, m | 3.85, m |
| 16 | 1.41, m; 1.48, m | 1.51, m; 1.58, m | 1.51, m; 1.66, m | 1.68, m; 1.77, m |
| 17 | 4.01, m | 3.93, m | 4.06, m | 4.17, m |
| 18 | 0.91, m; 1.60, m | 0.99, m; 1.37, m | 1.21, m; 1.56, m | 1.35, m; 1.49, m |
| 19 | 3.92, m | 3.88, m | 3.73, m | 3.79, m |
| 20 | 1.39, m; 1.47, m | 1.34, m; 1.43, m | 1.31, m; 1.36, m | 1.28, m |
| 21 | 3.86, m | 3.81, m | 3.78, m | 3.95, m |
| 22 | 1.36, m; 1.42, m | 1.39, m; 1.44, m | 1.16, m; 1.33, m | 1.29, m; 1.42, m |
| 23 | 3.72, m | 3.79, m | 3.75, m | 3.84, m |
| 24 | 1.39, m; 1.47 | 1.34, m; 1.43 | 1.11, m; 1.20 | 1.54, m |
| 25 | 3.86, m | 3.86, m | 3.84, m | 3.92, m |
| 26 | 0.76, m; 1.37, m | 0.87, m; 1.40, m | 0.68, m; 1.38, m | 0.97, m; 1.34, m |
| 27 | 4.28, m | 4.27, m | 4.29, m | 4.28, m |
| 28 | 5.33, dd (15.9, 4.9) | 5.38, dd (15.8, 4.1) | 5.36, dd (15.9, 3.6) | 5.40, dd (15.9, 4.3) |
| 29 | 5.53, dd (15.9, 5.9) | 5.47, dd (15.8, 5.8) | 5.48, dd (15.9, 5.2) | 5.65, dd (15.9, 5.2) |
| 30 | 2.56, m | 2.57, m | 2.57, m | 2.63, m |
| 31 | 4.63, dd (9.8, 2.8) | 4.65, dd (9.0, 2.7) | 4.68, dd (9.6, 2.8) | 4.81, dd (9.5, 2.6) |
| 32 | 1.88, m | 1.84, m | 1.84, m | 1.92, m |
| 33 | 0.91, d (6.7) | 0.89, d (6.7) | 0.91, d (6.6) | 0.93, d (7.0) |
| 34 | 0.82, d (6.7) | 0.79, d (6.7) | 0.81, d (6.6) | 0.90, d (7.0) |
| Me-14 | 0.70, d (6.9) | − | 0.78, d (6.8) | 0.95, d (7.0) |
| Me-30 | 1.03, d (7.0) | 0.97, d (6.9) | 0.97, d (6.8) | 1.03, d (7.0) |
| 37 | − | − | − | 1.49, s |
| 38 | − | − | − | 1.42, s |
| 40 | 1.37, s | 1.34, s | 1.35, s | 1.43, s |
| 41 | 1.23*^a^*, s | 1.23, s | 1.23, s | 1.39, s |
| 43 | 1.25, s | 1.25, s | 1.18, s | 1.30, s |
| 44 | 1.23, s | 1.19, s | 1.18, s | 1.30, s |
| 46 | 1.25*^a^*, s | 1.23, s | 1.28s | 1.37, s |
| 47 | 1.34, s | 1.32, s | 1.31 s | 1.39, s |
| OH-11 | 4.80, d (3.7) | 5.08, br.s | 4.68, d (3.0) | − |
| OH-13 | − | − | 4.44, br.s | − |
| OH-15 | 4.28, d (4.7) | 4.36, d (4.1) | − | − |

*Data were obtained in CDCl_3_-*d*; *^a^* Data with the same labels in each column may be interchanged.

Table S_6_. ^1^H-NMR data of compounds **4S**, **4R**, **9S**, and **9R** (500 MHz, in DMSO-*d*_6_, *J* = Hz)

| No. | **4S** | **4R** | **9S** | **9R** |
| --- | --- | --- | --- | --- |
| 2 | 5.94, d (15.2) | 5.93, d (15.1) | 5.89, d (15.2) | 5.89, d (15.1) |
| 3 | 7.18, dd (15.2, 11.4) | 7.17, dd (15.1, 11.3) | 7.17, dd (15.2, 11.6) | 7.19, dd (15.1, 11.4) |
| 4 | 6.48, dd (14.9, 11.4) | 6.48, dd (14.8, 11.3) | 6.47, dd (14.8, 11.6) | 6.48, dd (14.8, 11.4) |
| 5 | 6.71, dd (14.9, 10.6) | 6.71, dd (14.8, 11.0) | 6.68, dd (14.8, 11.4) | 6.71, dd (14.8, 11.0) |
| 6 | 6.38, dd (14.9, 10.6) | 6.41, dd (14.7, 11.0) | 6.17, dd (14.9, 11.4) | 6.32, dd (14.9, 11.0) |
| 7 | 6.45, dd (14.9, 10.6) | 6.45, dd (14.7, 11.1) | 6.33, dd (14.9, 11.4) | 6.45, dd (14.9, 10.7) |
| 8 | 6.32, dd (14.9, 10.6) | 6.37, dd (15.0, 11.1) | 5.91, dd (15.0, 11.4) | 6.20, dd (15.2, 10.7) |
| 9 | 5.89, dd (14.9, 7.3) | 5.90, dd (15.0, 7.2) | 5.57, dd (15.0, 8.3) | 5.70, dd (15.2, 8.9) |
| 10 | 4.26, d (7.3) | 4.40, d (7.2) | 3.65, m | 3.87, m |
| 11 | 5.22, m | 5.24, m | 5.08, m | 5.11, m |
| 12 | 2.02, m, | 1.99, m | 2.01, m; 2.39, m | 2.01, m; 2.26, m |
| 13 | 3.83, m | 3.85, m | 5.29, m | 5.34, m |
| 14 | 2.08, m | 2.12, m | 2.02, m | 1.99, m |
| 15 | 5.30, m | 5.30, m | 3.21, m | 3.28, m |
| 16 | 1.59, m; 2.02, m | 1.50, m; 1.90, m | 1.45, m; 1.63, m | 1.41, m; 1.63, m |
| 17 | 3.68, m | 3.62, m | 3.56, m | 3.62, m |
| 18 | 1.37, m; 1.46, m | 1.34, m; 1.43, m | 1.36, m | 1.36, m |
| 19 | 3.89, m | 3.88, m | 3.84, m | 3.88, m |
| 20 | 1.13, 1.35 | 1.13, 1.35 | 0.90, 1.26 | 1.00, 1.36 |
| 21 | 3.95, m | 3.96, m | 3.83, m | 3.87, m |
| 22 | 1.03, m; 1.11, m | 1.03, m; 1.11, m | 1.00, m; 1.26, m | 1.00, m; 1.25, m |
| 23 | 3.88, m | 3.89, m | 3.83, m | 3.90, m |
| 24 | 1.08, m; 1.34, m | 1.08, m; 1.34, m | 0.90, m; 1.32, m | 0.96, m; 1.25, m |
| 25 | 3.76, m | 3.76, m | 3.66, m | 3.70, m |
| 26 | 1.10, m; 1.34, m | 1.10, m; 1.34, m | 1.04, m; 1.31, m | 1.10, m; 1.30, m |
| 27 | 4.08, m | 4.08, m | 4.10, m | 4.13, m |
| 28 | 5.40, dd (15.9, 4.3) | 5.40, dd (16.0, 4.2) | 5.43, dd (15.9, 3.4) | 5.45, dd (15.6, 3.8) |
| 29 | 5.49, dd (15.9, 5.0) | 5.49, dd (16.0, 5.1) | 5.51, dd (15.9, 4.3) | 5.54, dd (15.6, 4.8) |
| 30 | 2.56, m | 2.56, m | 2.56, m | 2.57, m |
| 31 | 4.66, dd (9.9, 2.2) | 4.66, dd (9.9, 2.2) | 4.61, dd (10.0, 1.5) | 4.65, dd (10.1, 2.1) |
| 32 | 1.84, m | 1.84, m | 1.80 m | 1.83, m |
| 33 | 0.92, d (6.7) | 0.92, d (6.6) | 0.89, d (6.7) | 0.92, d (6.8) |
| 34 | 0.81, d (6.7) | 0.81, d (6.6) | 0.77, d (6.7) | 0.81, d (6.8) |
| Me-14 | 0.69, d (7.0) | 0.83, d (7.0) | 0.79, d (6.9) | 0.57, d (6.8) |
| Me-30 | 0.97, d (6.9) | 0.97, d (6.8) | 0.94, d (6.7) | 0.97, d (6.9) |
| OH-17 | 4.67, d (5.5) | 4.63, d (5.1) | 4.71, d (5.2) | 4.33, d (4.8) |
| OH-19 | 4.29, d (4.9) | 4.29, d (4.8) | 4.46, d (5.5) | 3.93, d (5.5) |
| OH-21 | 4.01, d (5.9) | 4.02, d (5.7) | 4.18, d (5.4) | 3.82, d (5.8) |
| OH-23 | 4.10, d (5.8) | 4.08, d (5.5) | 4.20, d (6.1) | 4.19, d (5.2) |
| OH-25 | 4.41, d (4.9) | 4.42, d (4.9) | 4.54, d (5.5) | 4.37, d (5.0) |
| OH-27 | 4.71, d (3.2) | 4.72, d (3.0) | 4.81, d (3.3) | 4.65, d (2.9) |
| Ar-H | 7.44−7.49, 10H, m | 7.42−7.48, 10H, m | 7.25−7.45, 10H, m | 7.36−7.50, 10H, m |
| OCH_3_ | 3.43, s; 3.45, s | 3.39, s; 3.46, s | 3.38, s; 3.38, s | 3.39, s; 3.45, s |

**General Experimental Procedures.** UV and IR spectra were recorded on METASH UV-8000 (Shanghai METASH Instruments Co. Ltd., China) and AVATAR 370 FT-IR spectrometer (Thermo Nicolet), respectively. HRESIMS data were obtained from an Agilent 6230 TOF LC/MS spectrometer. Each sample was dissolved in the DMSO-*d*_6_ or CDCl_3_-*d* of 500 μL and then transferred to a brown NMR tube (80 × 5 mm) for NMR analysis. NMR spectra were acquired on a Bruker 500 spectrometer using standard pulse programs and acquisition parameters and chemical shifts were expressed in *δ* (ppm). Diaion HP-20 (Mitsubishi Chemical, Japan) and octadecyl-functionalized silica gel (ODS, Cosmosil 75C_18_ Prep, Nacalai Tesque Inc., Japan) were used for column chromatography. HPLC separation was performed on a CXTH LC-3000 prepared HPLC system (Beijing Chuangxintongheng Science & Technology Co., Ltd., China) using a Sepax Amethyst C_18_-H column (250 × 21.2 mm, 5μm, Sepax Technologies, USA) or an Agilent 1260 HPLC system using an Agilent Zorbax SB-C_18_ column (250 × 9.4 mm, 5 μm). All solvents used in this study were purchased from the Sinopharm Chemical Reagent Co. Ltd. (Shanghai, China). Columns for chromatography and containers for sample collection and storage were brown glasses. Human glioma U251, U87-MG, SHG-44 cells, rat glioma C6 cells, normal human foreskin fibroblast (HFF-1), and normal human astrocytes (HA) were purchased from the Cell Bank of the Chinese Academy of Sciences. Methicillin-resistant *Staphylococcus aureus* ATCC 43300, *Escherichia coli* ATCC 25922, and *Candida albicans* were gifts from Drs. Zhongjun Ma, Pinmei Wang, and Bin Wu, respectively. Doxorubicin (DOX, >98.0%) was ordered from Sigma-Aldrich, gentamicin (99.6%), and amphotericin B (>95.0%) from Meilune Biotechnology Co. Ltd. (Dalian, China). Anhydrous dichloromethane, anhydrous pyridine, pyridinium-*p*-toluenesulfonate, 2,2-dimethoxypropane, 4-dimethylaminopyridine, (*S*)-(+)-α- methoxy-α-(trifluoromethyl)phenylacetyl chloride [(*S*)-(+)-MTPA-Cl)], and (*R*)-(−)-α- methoxy-α-(trifluoromethyl)phenylacetyl chloride [(*R*)-(−)-MTPA-Cl)] were purchased from Aladdin Industrial Corporation (Shanghai, China). Mueller Hinton Broth (MHB), Nutrient Broth (NB), and Gause′s-agar were purchased from Hangzhou Microbial Reagent Co. Ltd. (Hangzhou, China) and [Guangdong Huankai Microbial Science and Technology Co. Ltd.](http://huankai.en.alibaba.com/) (Guangzhou, China), respectively.

Figure S_3_. ^1^H-NMR spectrum of flavofungin I (**1**)


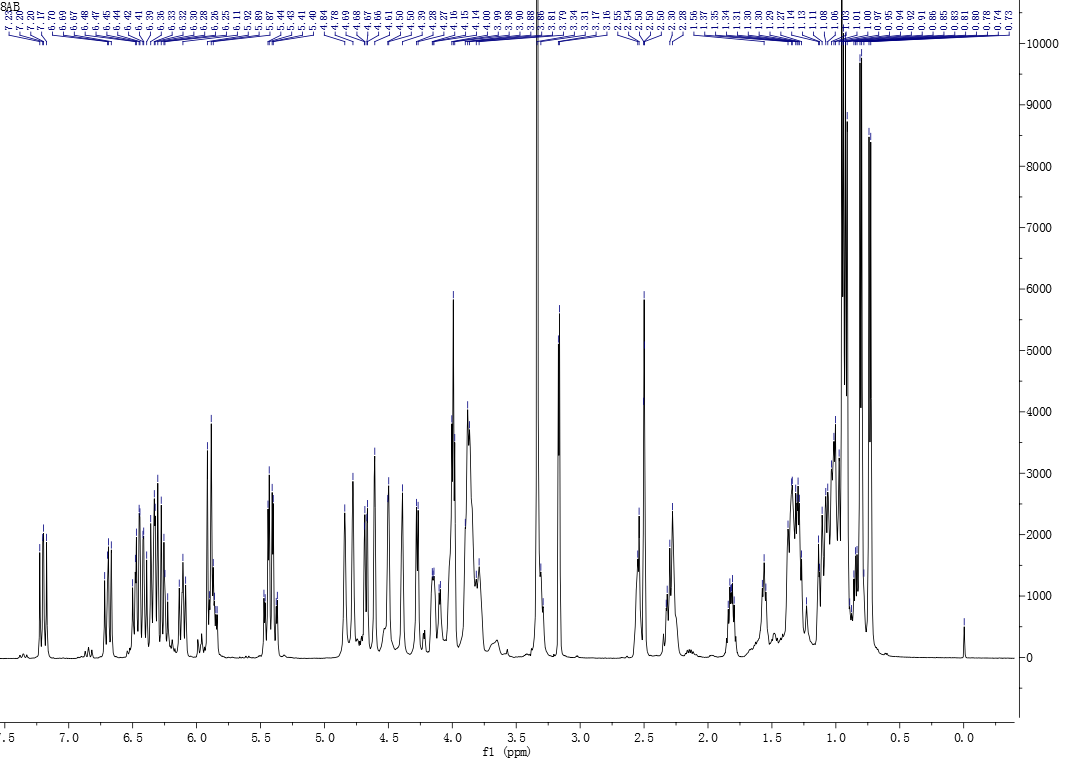


Figure S_4_. ^13^C-NMR spectrum of flavofungin I (**1**)


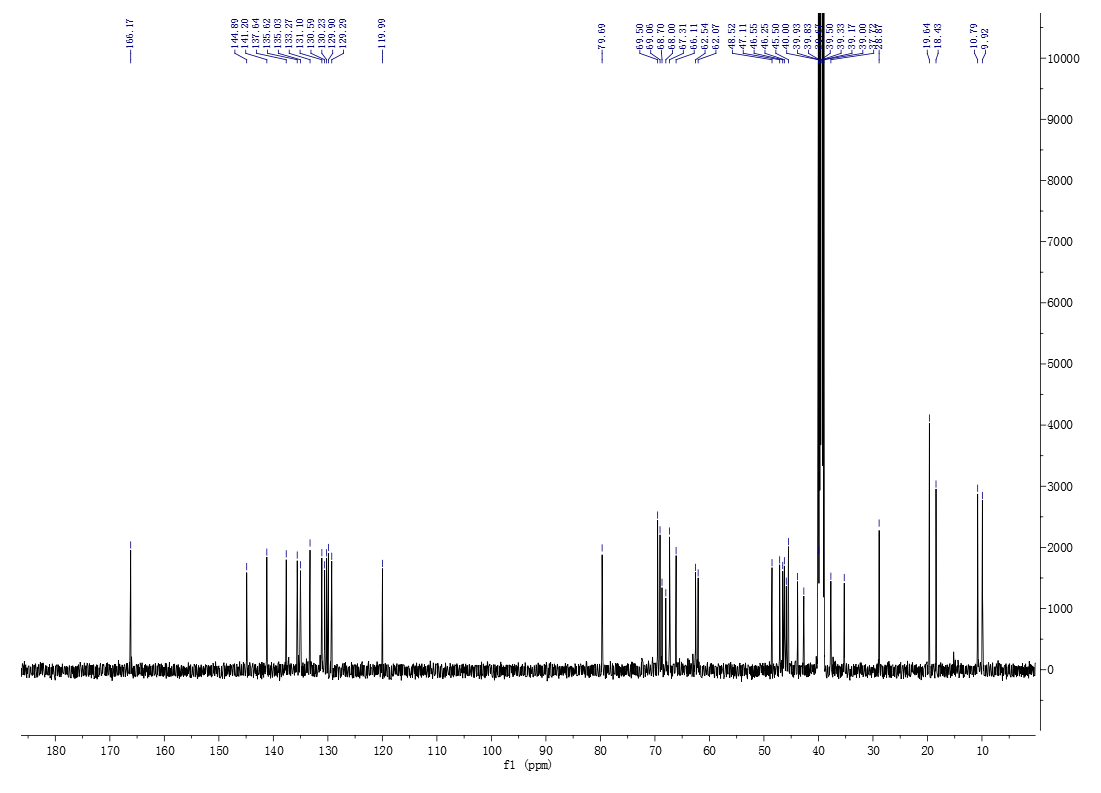


Figure S_5_. ^1^H-^1^H COSY spectrum of flavofungin I (**1**)


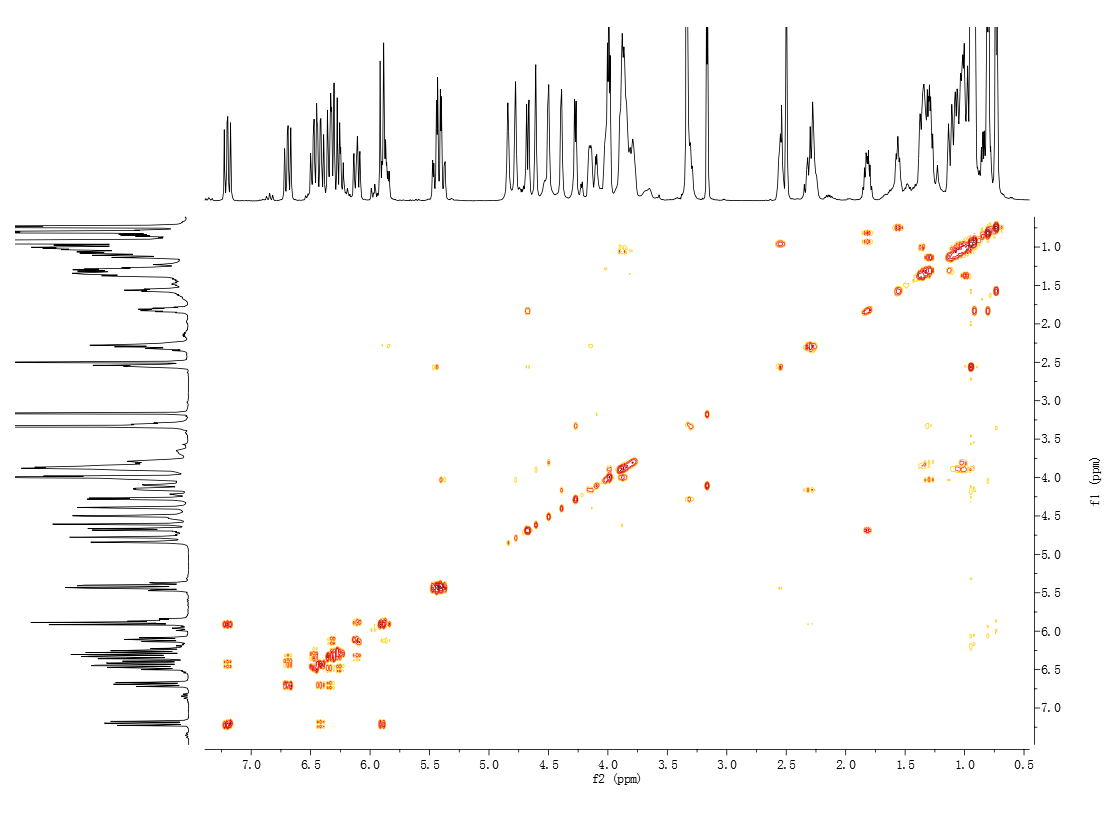


Figure S_6_. HSQC spectrum of flavofungin I (**1**)


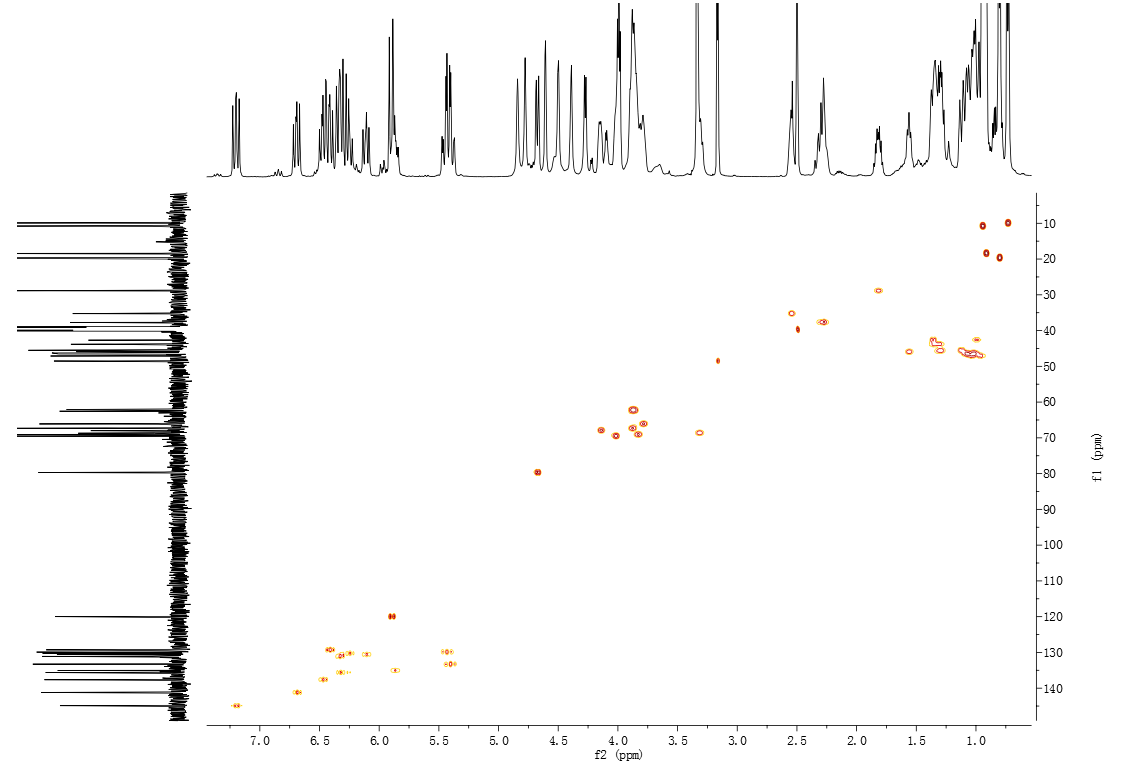


Figure S_7_. HMBC spectrum of flavofungin I (**1**)


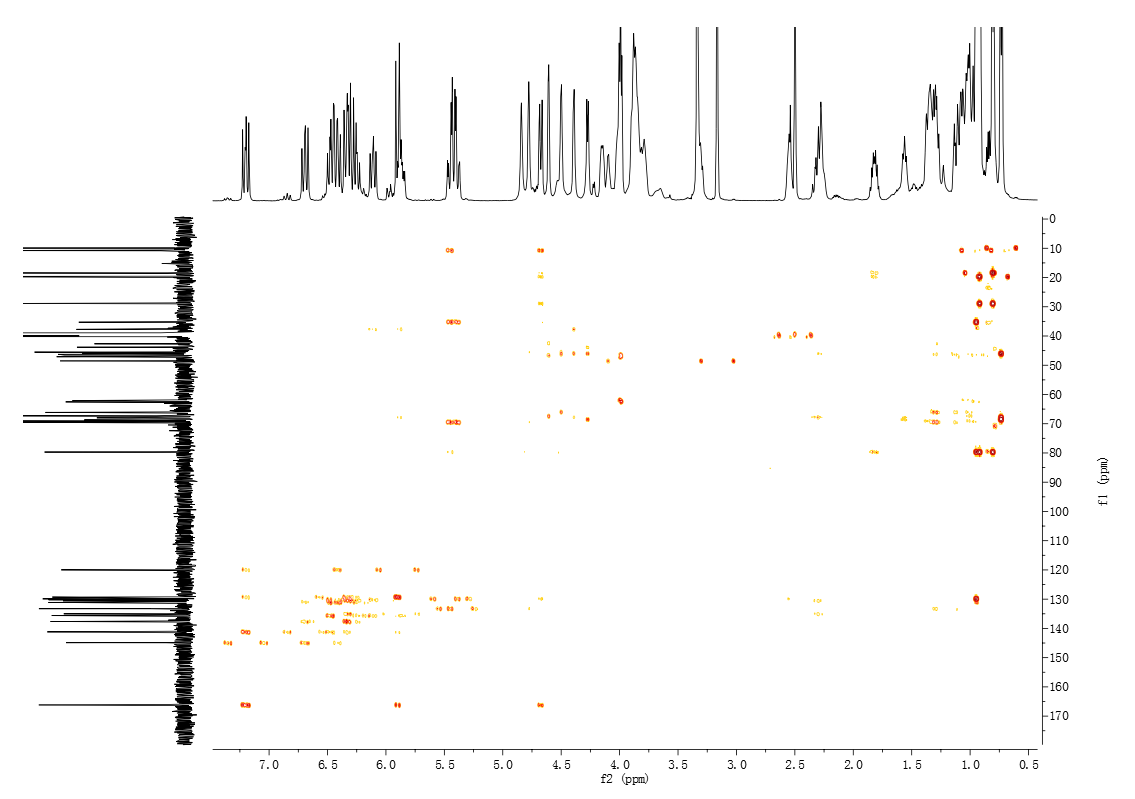


Figure S_8_. HRESIMS of Flavofungin I (**1**)

Figure S_9_. ^1^H-NMR spectrum of flavofungin II (**2**)


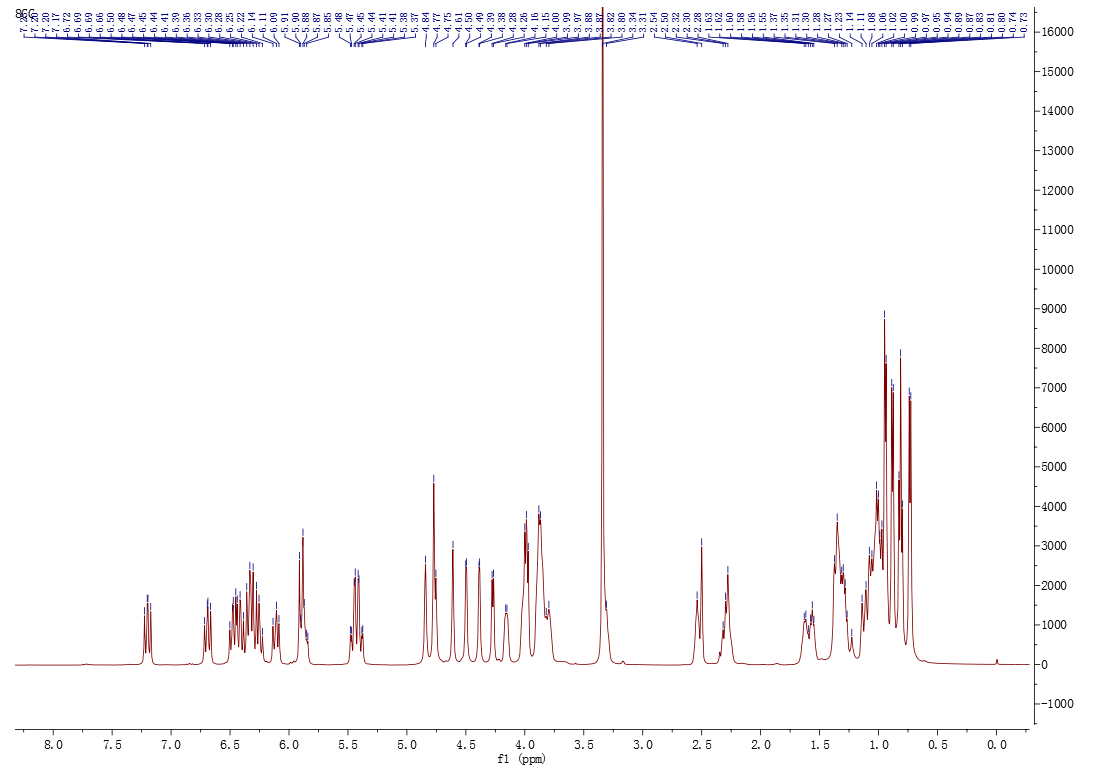


Figure S_10_. ^13^C-NMR spectrum of flavofungin II (**2**)


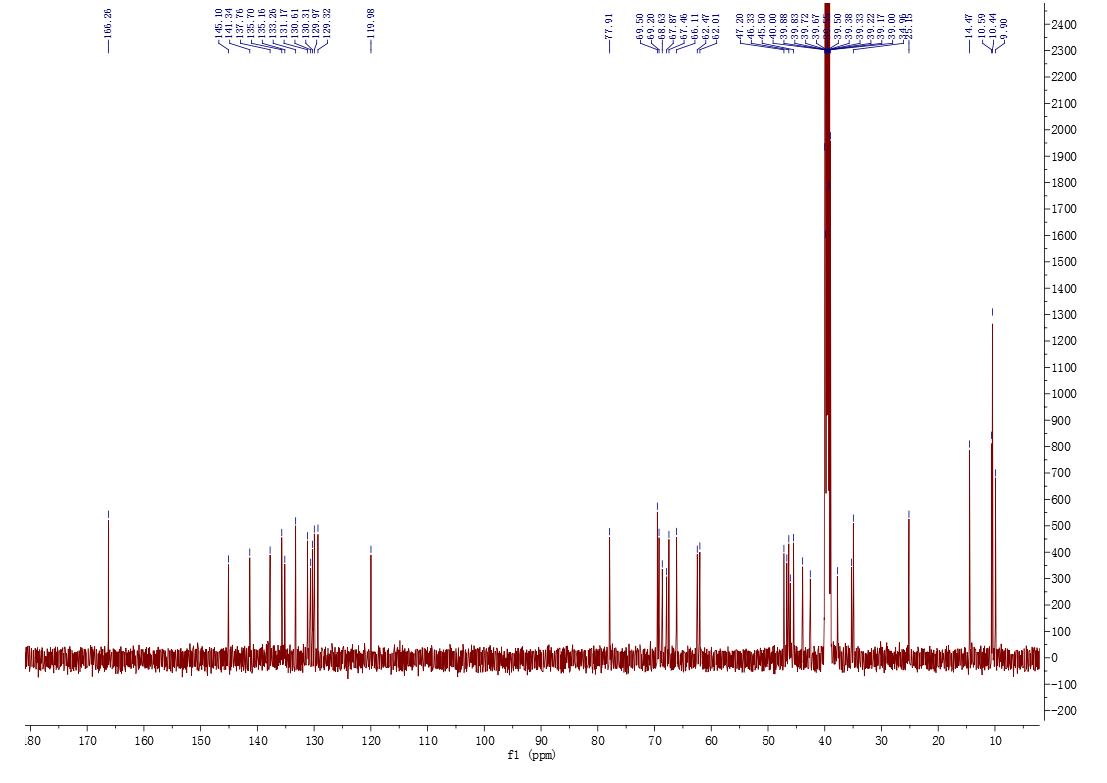


Figure S_11_. ^1^H-^1^H COSY spectrum of flavofungin II (**2**)


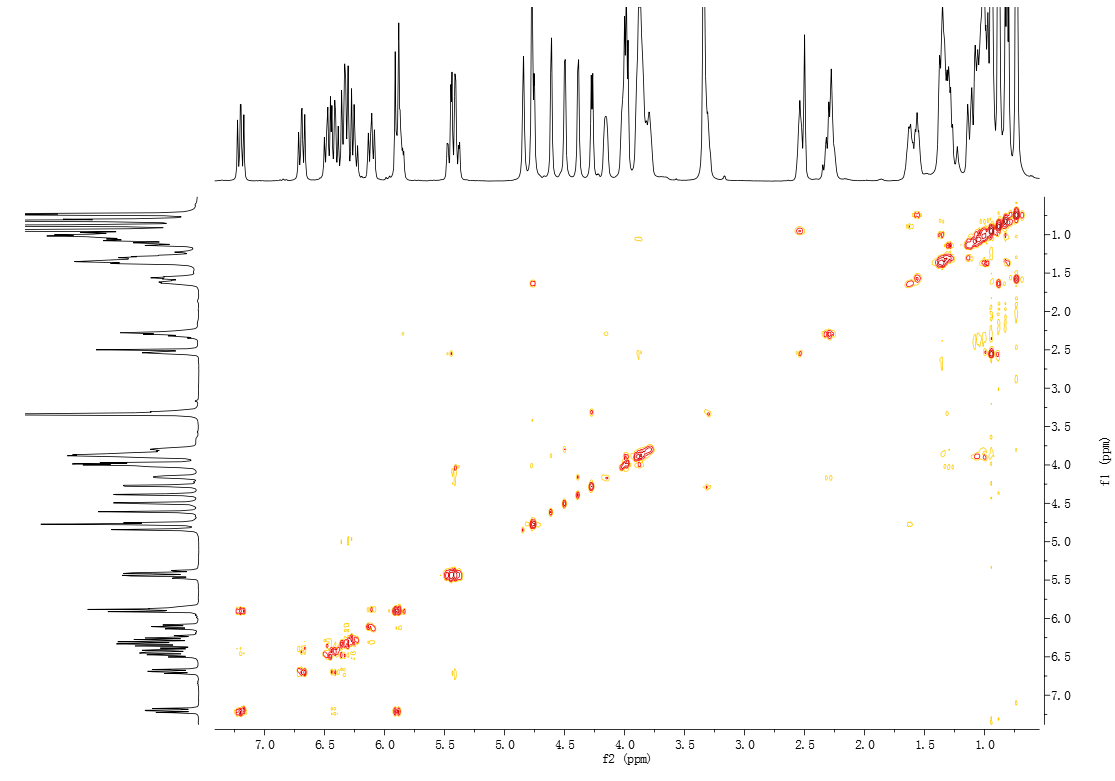


Figure S_12_. HSQC spectrum of flavofungin II (**2**)


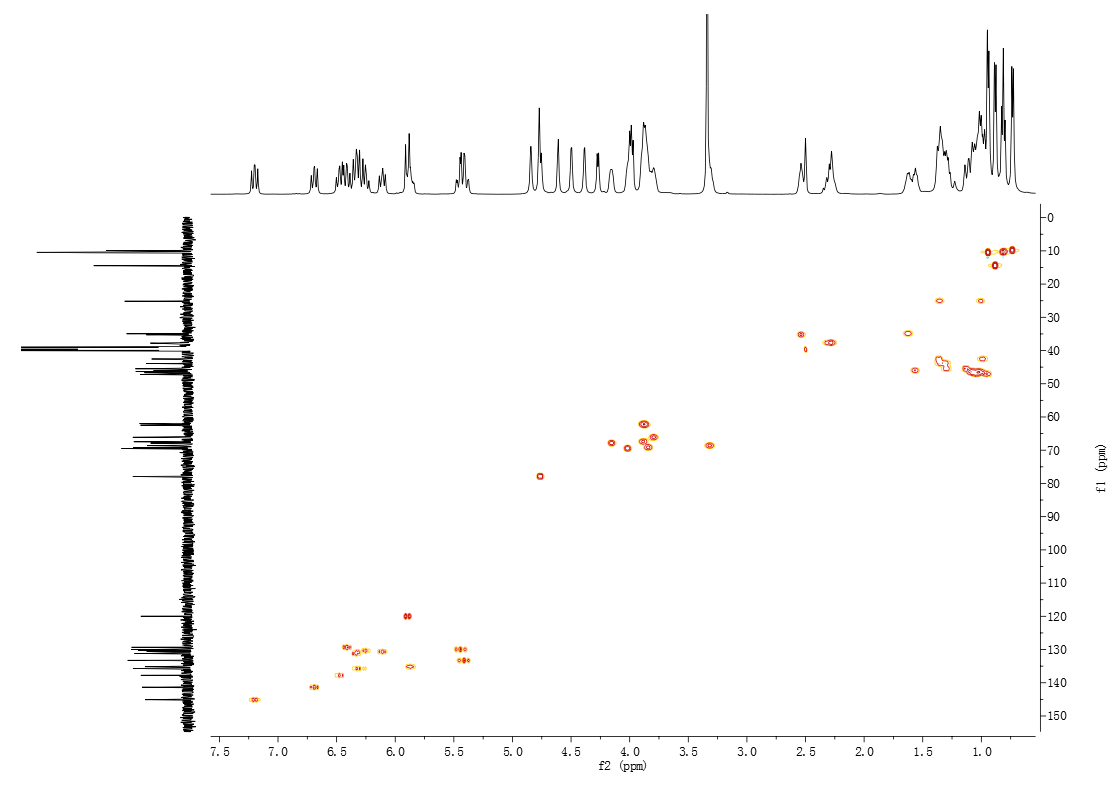


Figure S_13_. HRESIMS of flavofungin II (**2**)

Figure S_14_. ^1^H-NMR spectrum of flavofungin III (**3**)


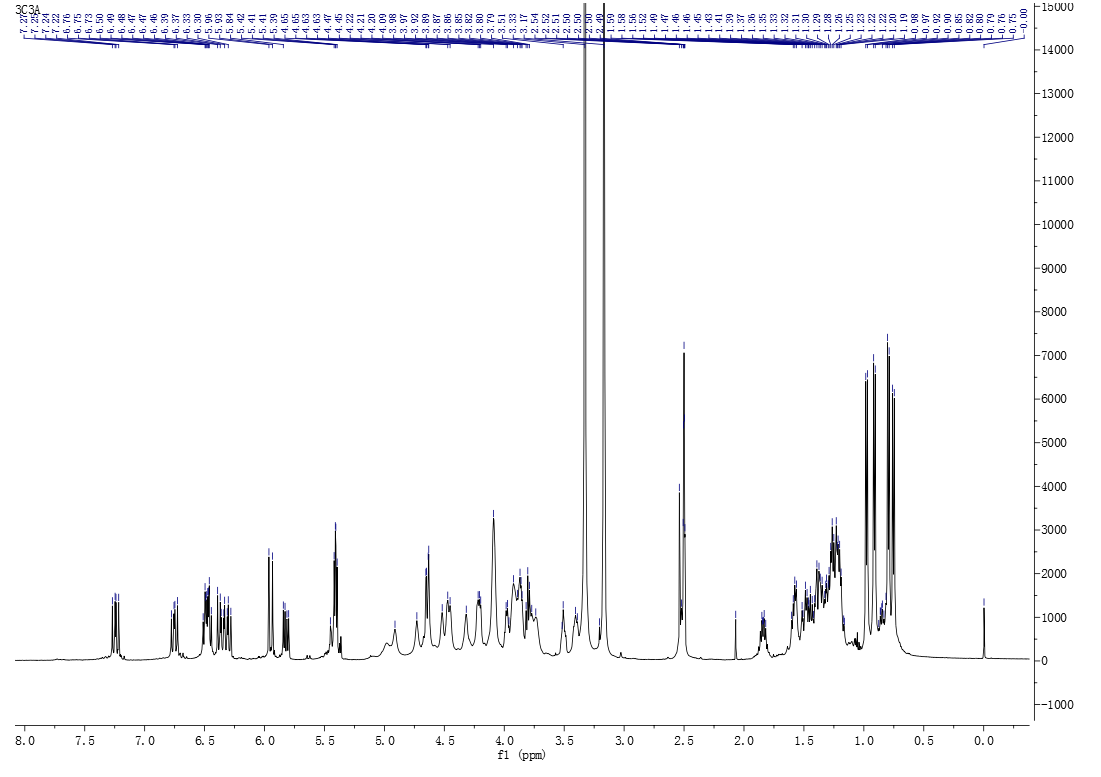


Figure S_15_. ^13^C-NMR spectrum of flavofungin III (**3**)


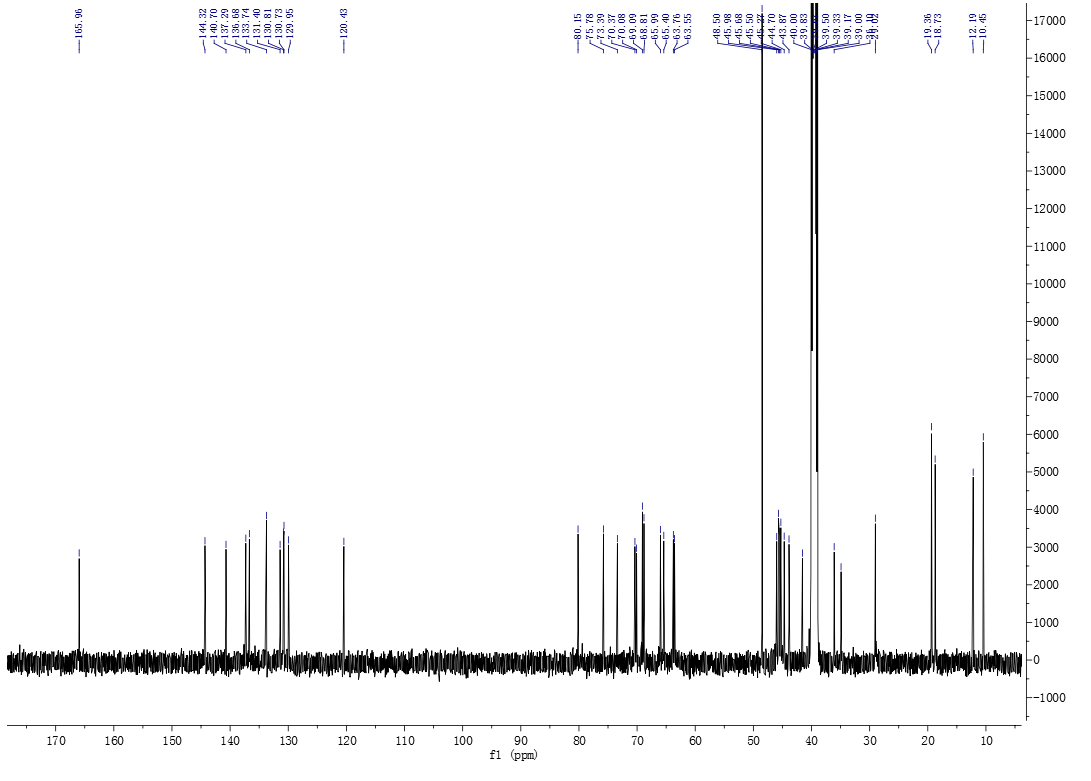


Figure S_16_. ^1^H-^1^H COSY spectrum of flavofungin III (**3**)


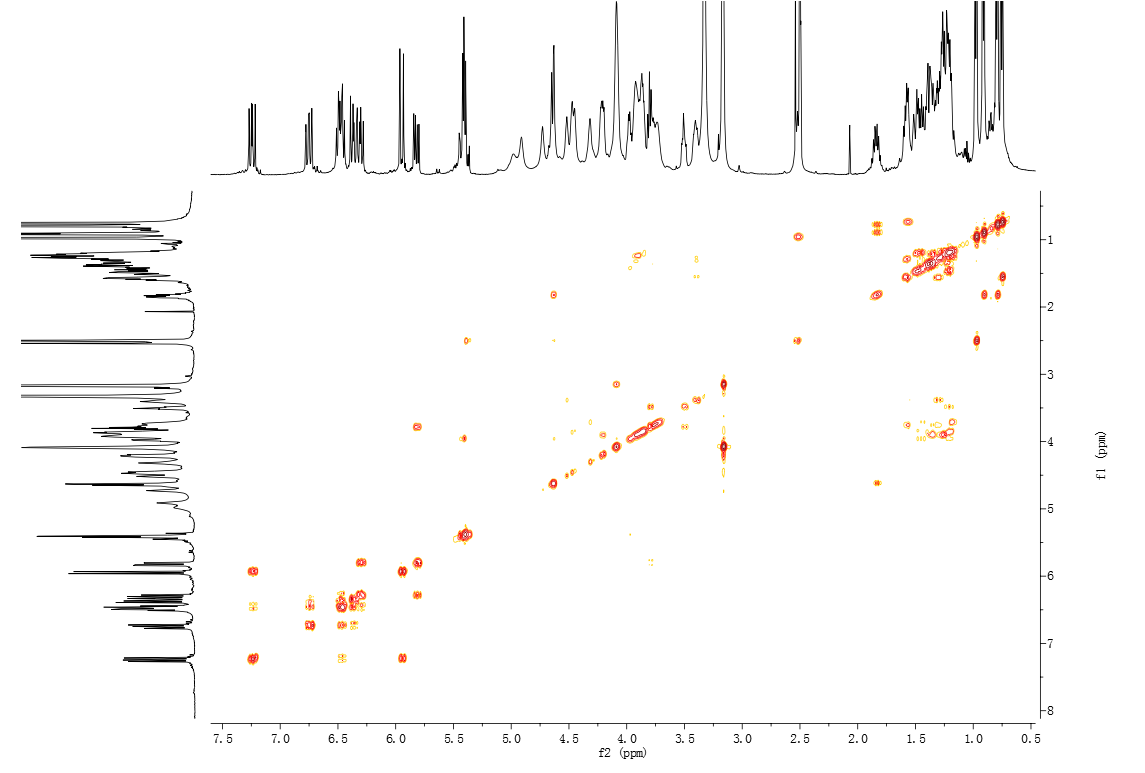


Figure S_17_. HSQC spectrum of flavofungin III (**3**)


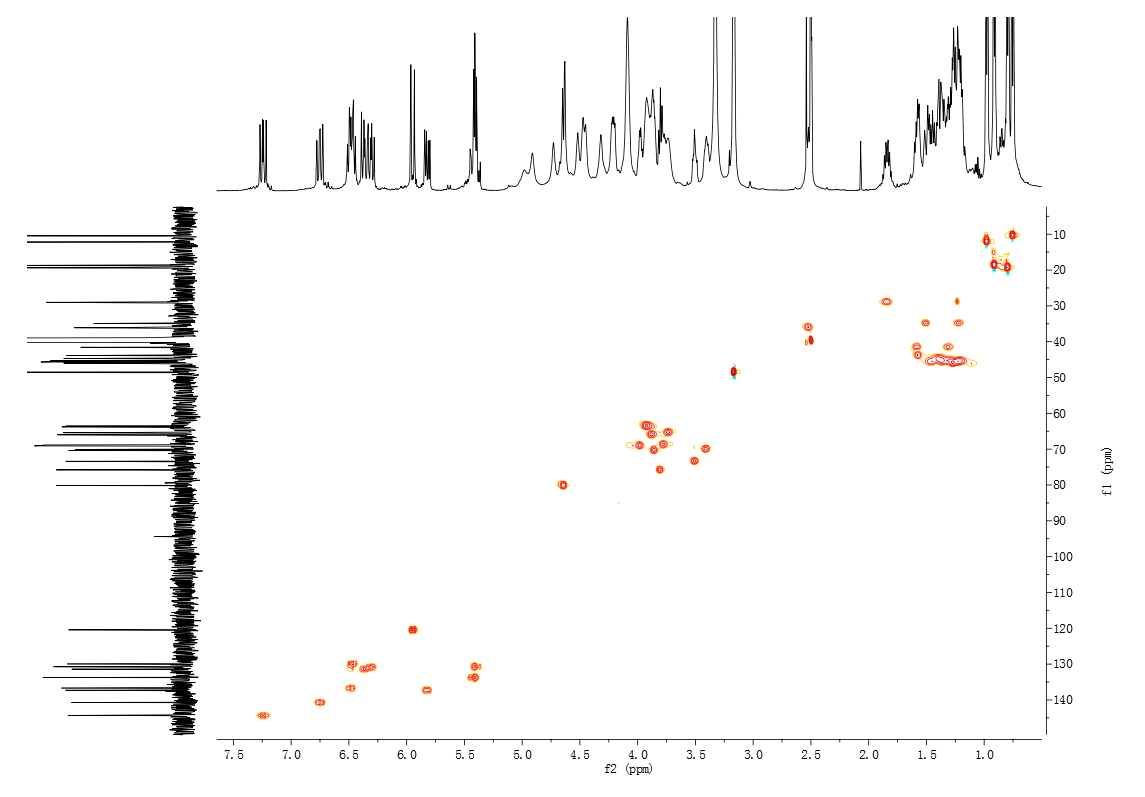


Figure S_18_. HMBC spectrum of flavofungin III (**3**)


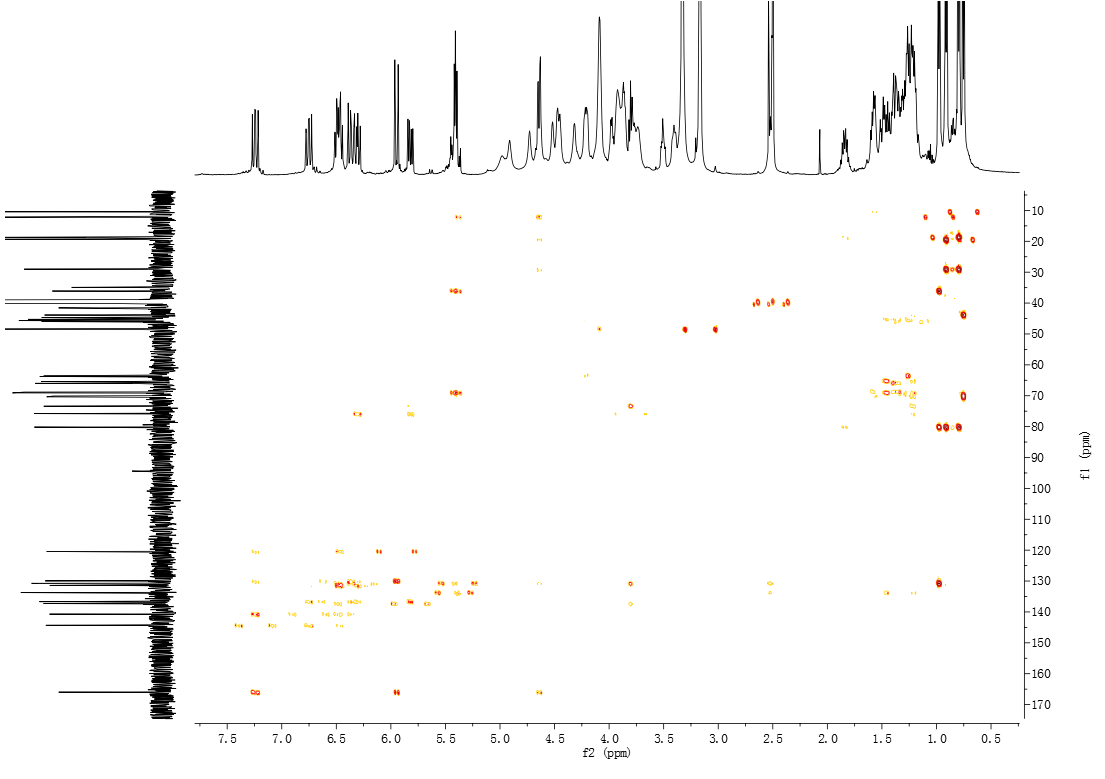


Figure S_19_. HRESIMS of flavofungin III (**3**)

Figure S_20_. ^1^H-NMR spectrum of compound **3a**


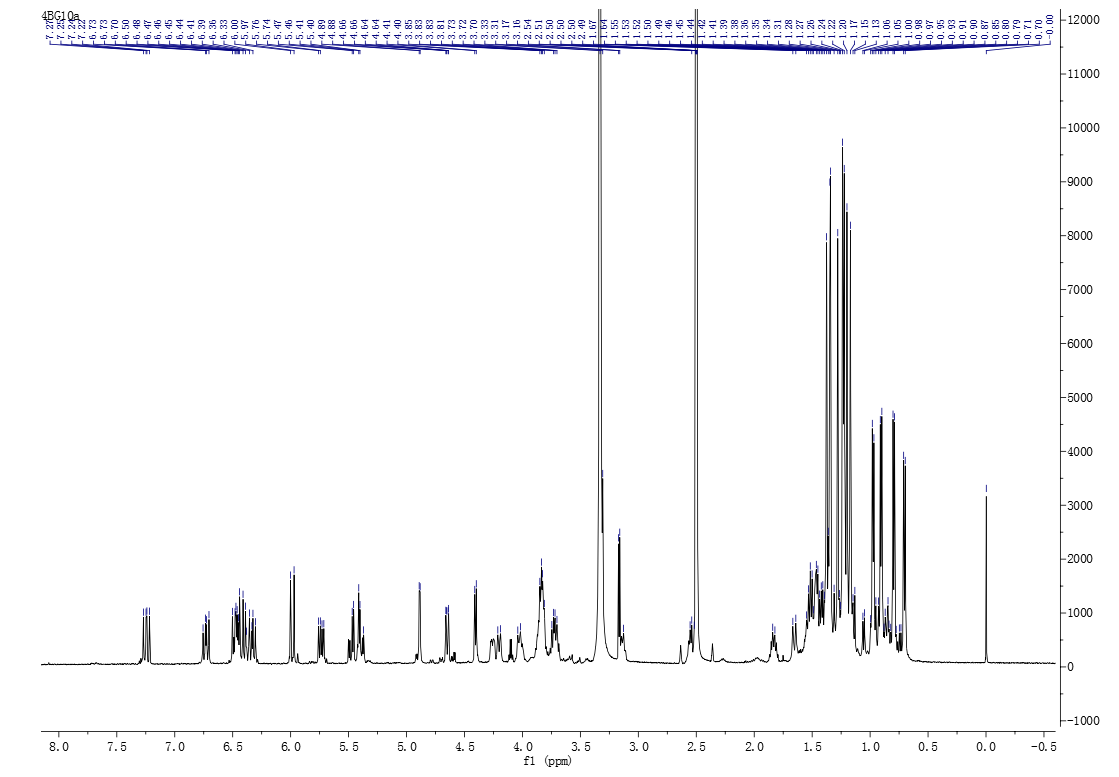


Figure S_21_. HSQC spectrum of compound **3a**


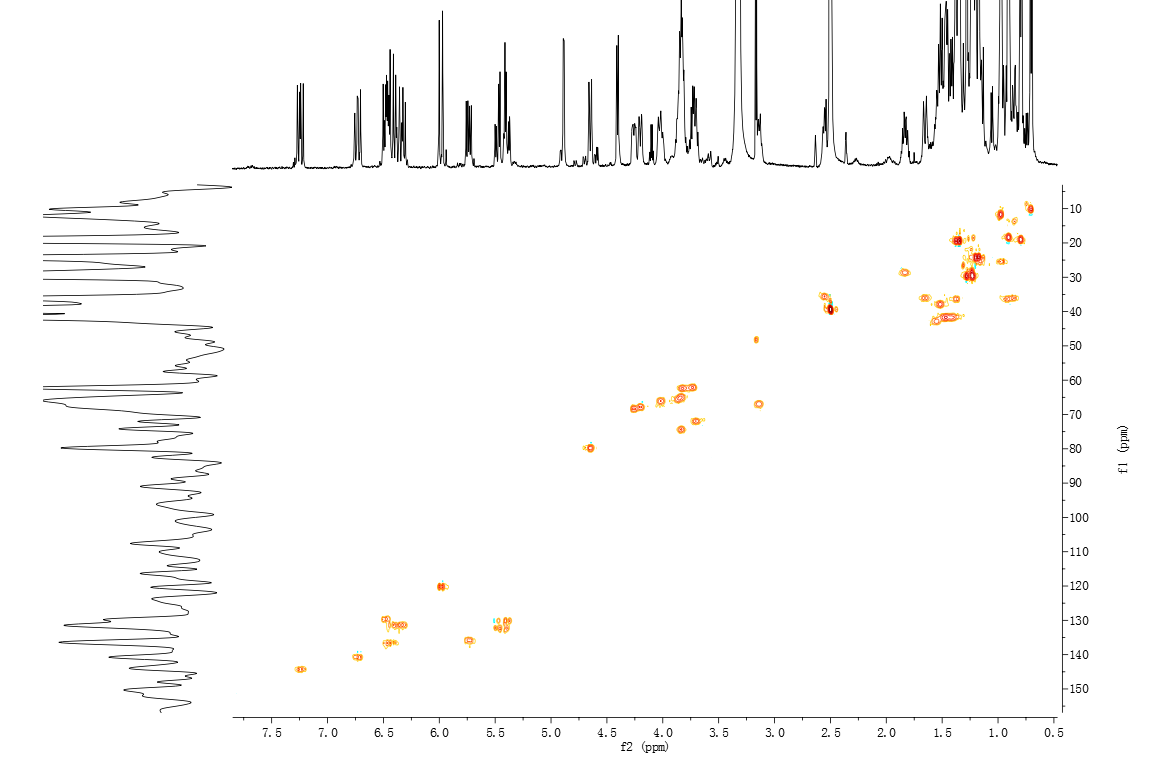


Figure S_22_. ^1^H-^1^H COSY spectrum of compound **3a**

**
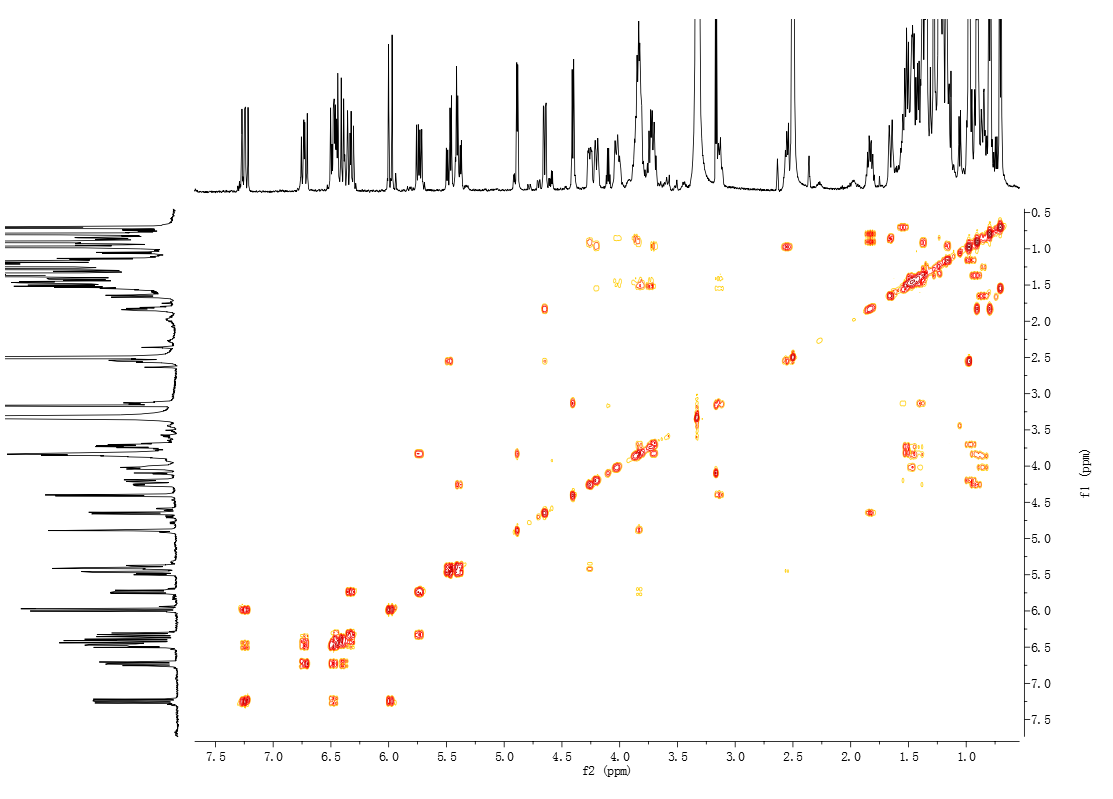
**

Figure S_23_. NOESY spectrum of compound **3a**


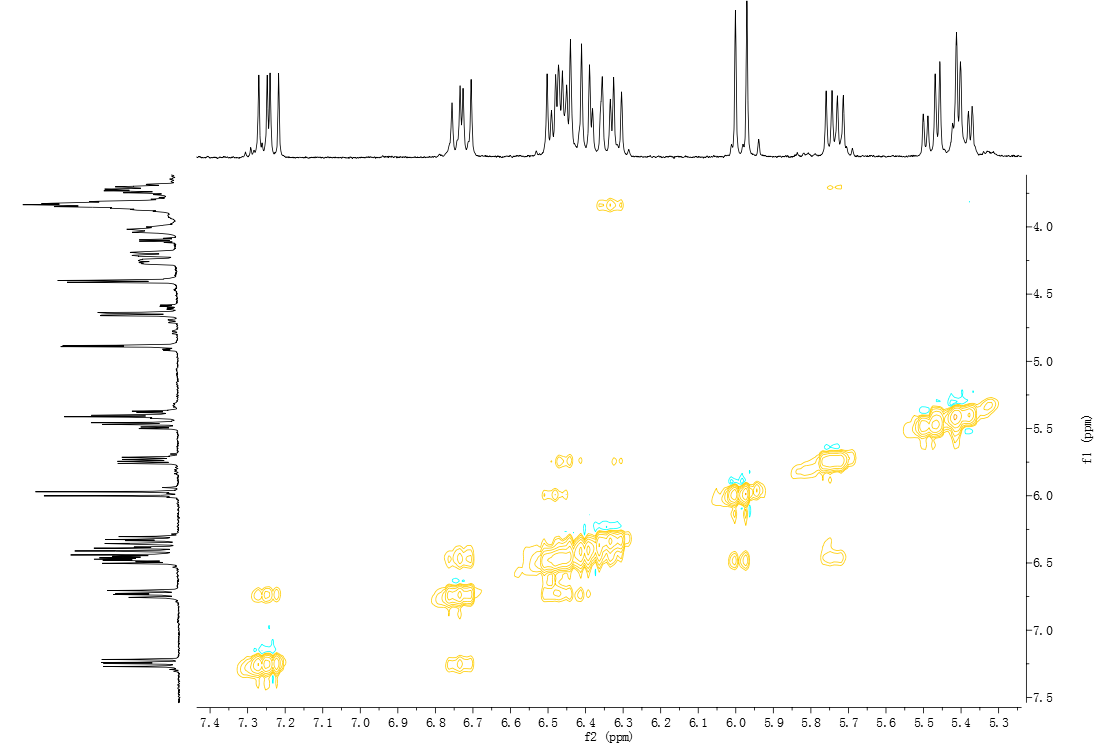


Figure S_24_. NOESY spectrum of compound **3a**


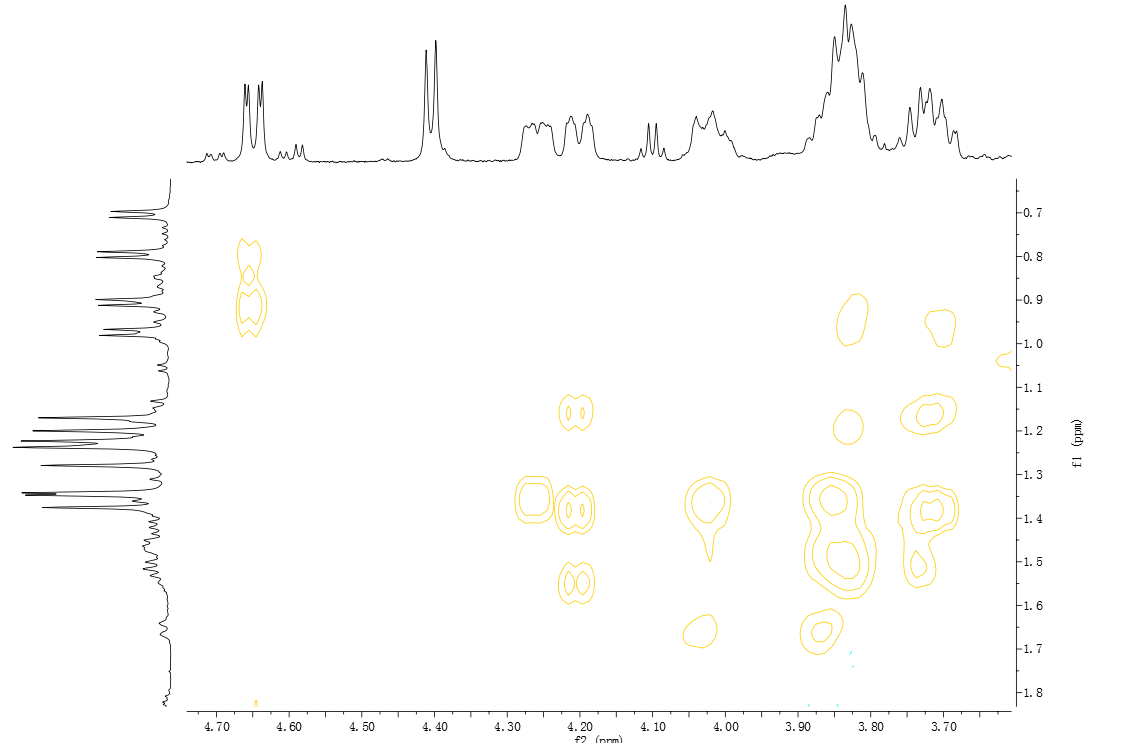


Figure S_25_. NOESY spectrum of compound **3a**


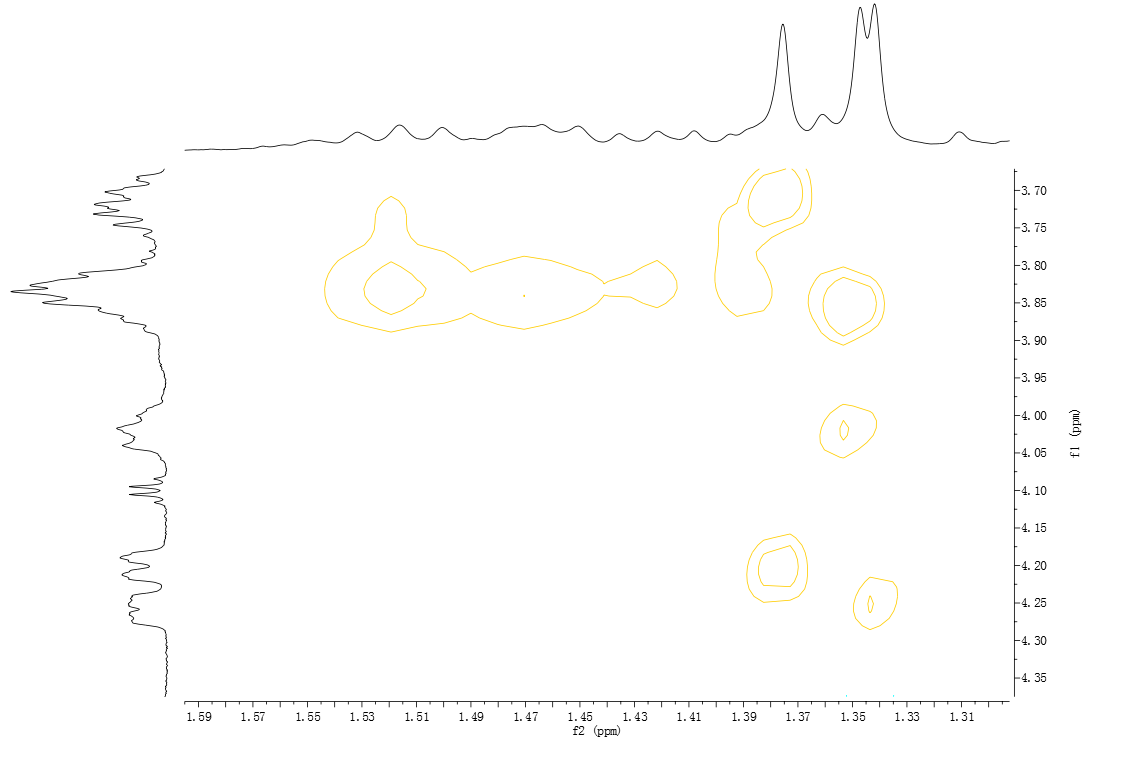


Figure S_26_. HRESIMS of compound **3a**

**
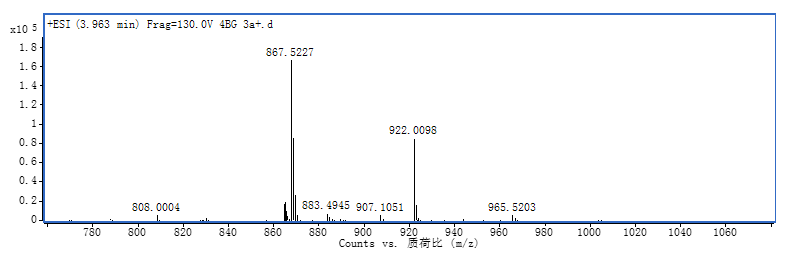
**

Figure S_27_. ^1^H-NMR spectrum of flavofungin IV (**4**)


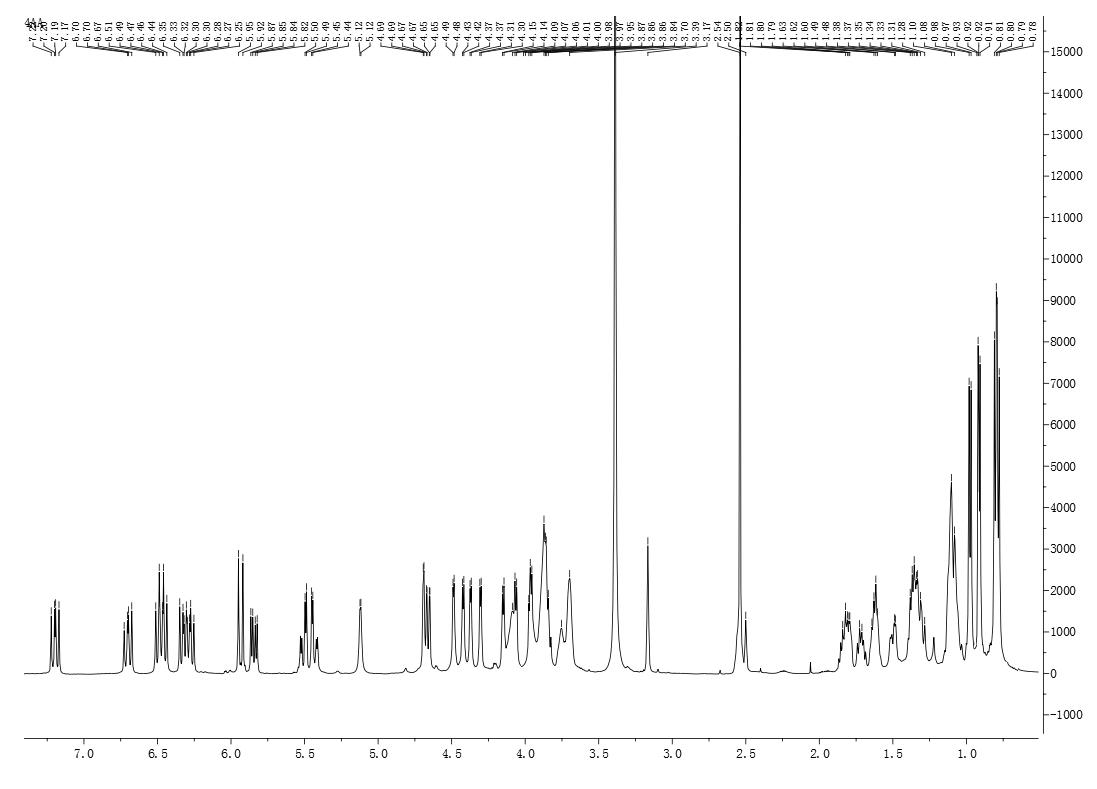


Figure S_28_. ^13^C-NMR spectrum of flavofungin IV (**4**)


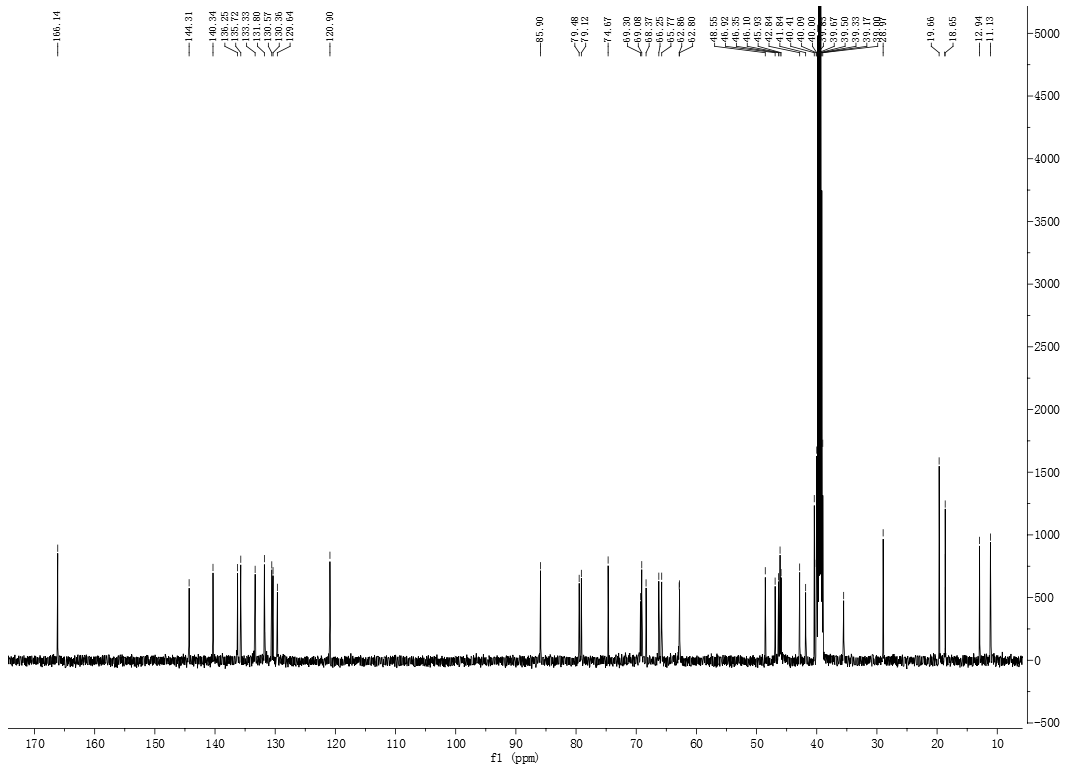


Figure S_29_. ^1^H-^1^H COSY spectrum of flavofungin IV (**4**)


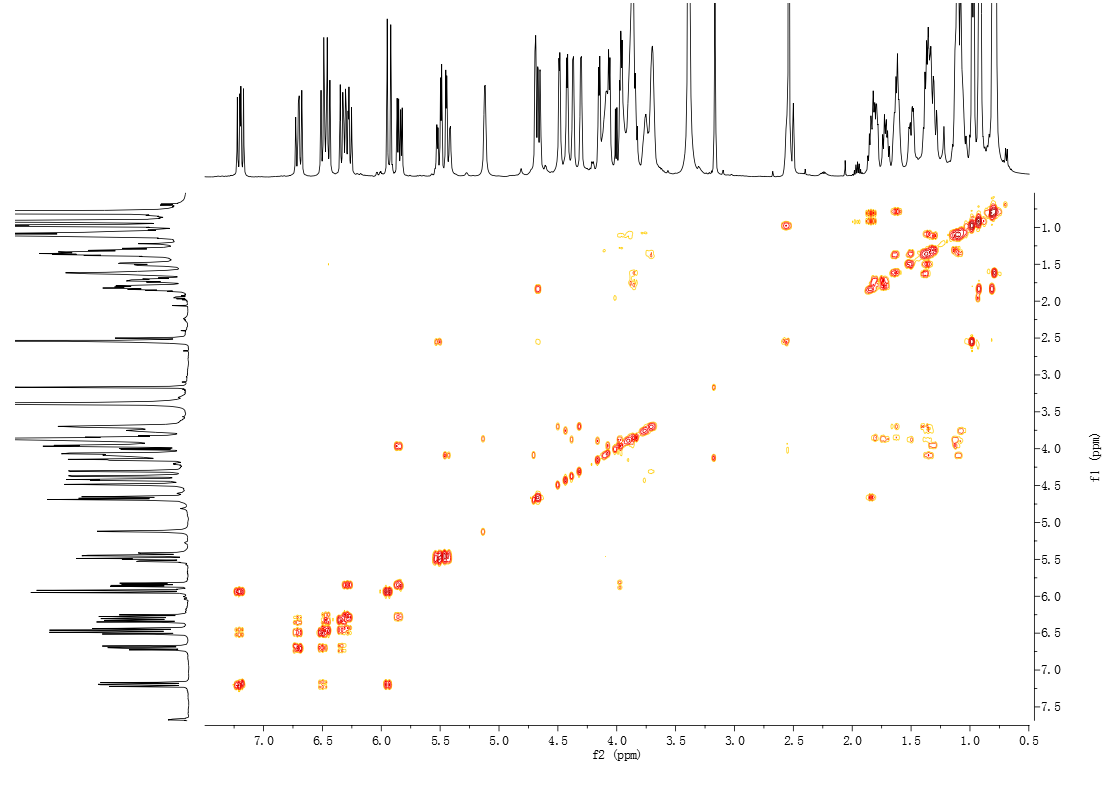


Figure S_30_. HSQC spectrum of flavofungin IV (**4**)

**
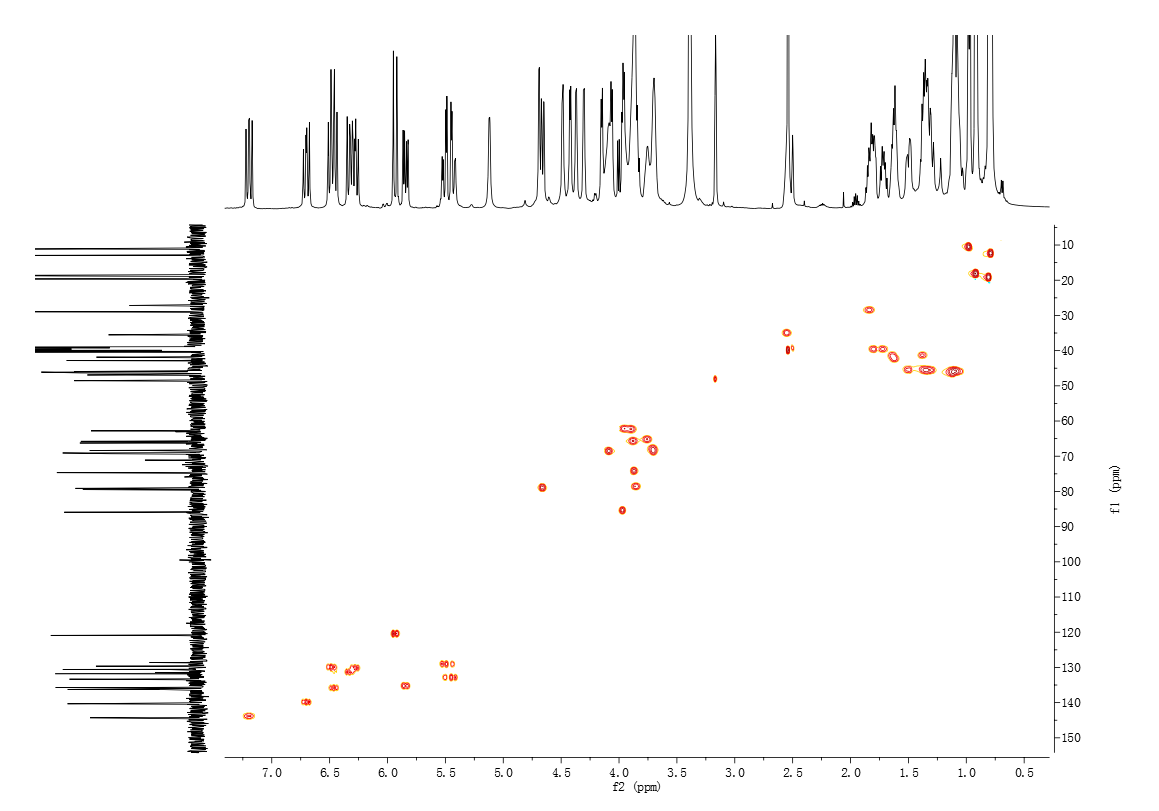
**

Figure S_31_. HMBC spectrum of flavofungin IV (**4**)


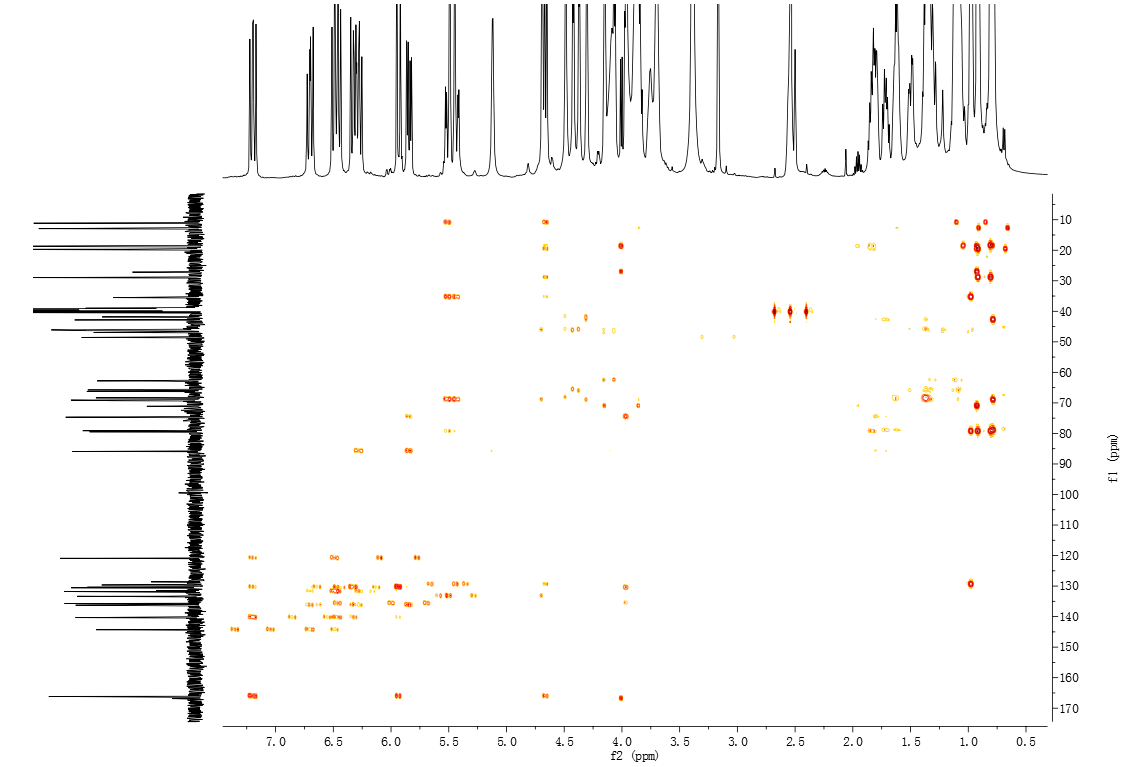


Figure S_32_. HRESIMS of flavofungin IV (**4**)

Figure S_33_. ^1^H-NMR spectrum of compound **4a**


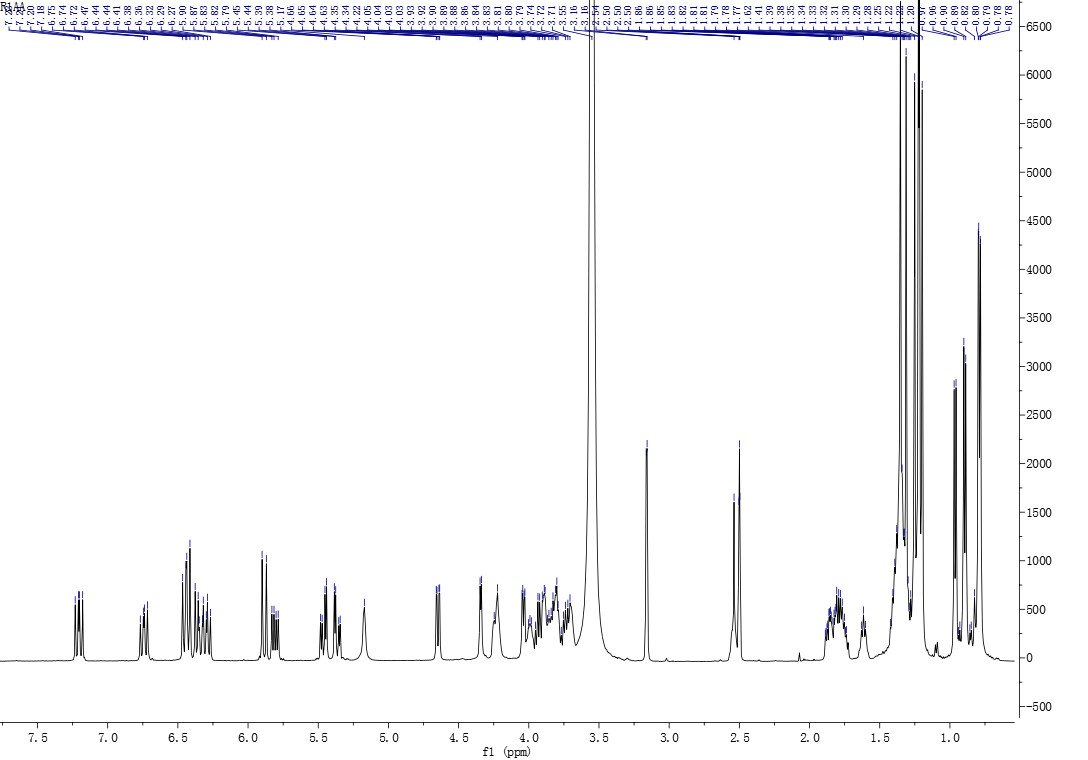


Figure S_34_. ^13^C-NMR spectrum of compound **4a**


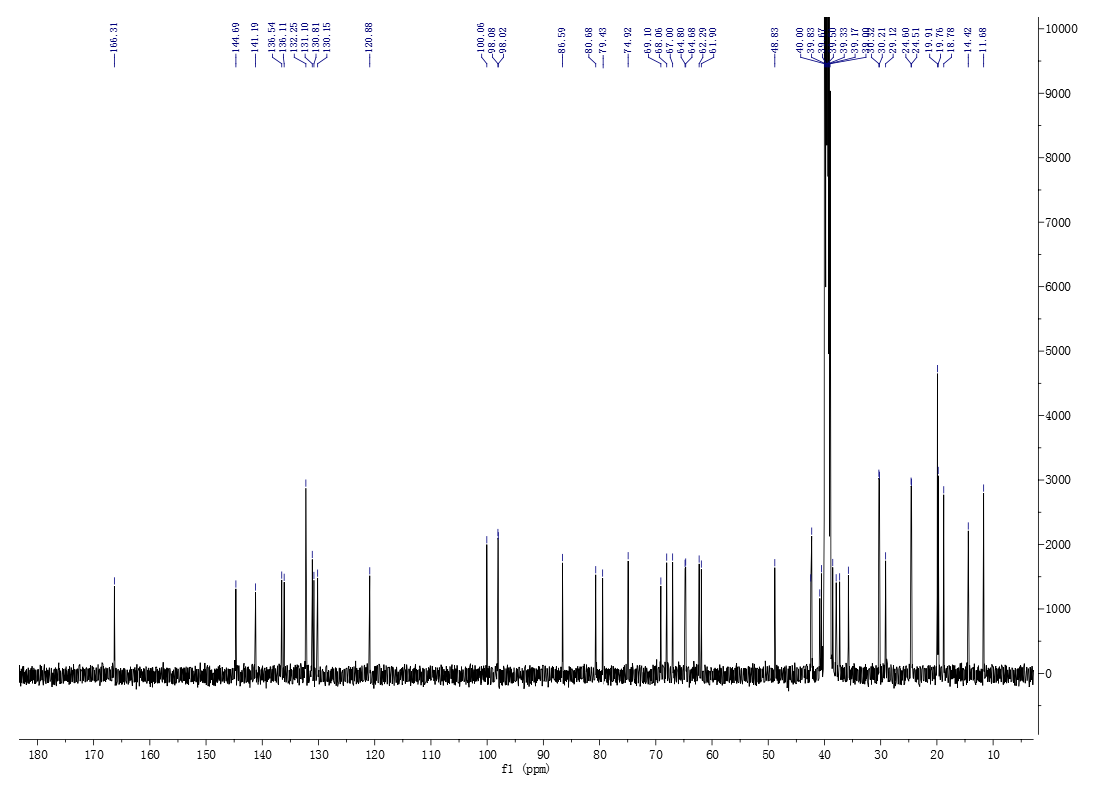


Figure S_35_. HSQC spectrum of compound **4a**


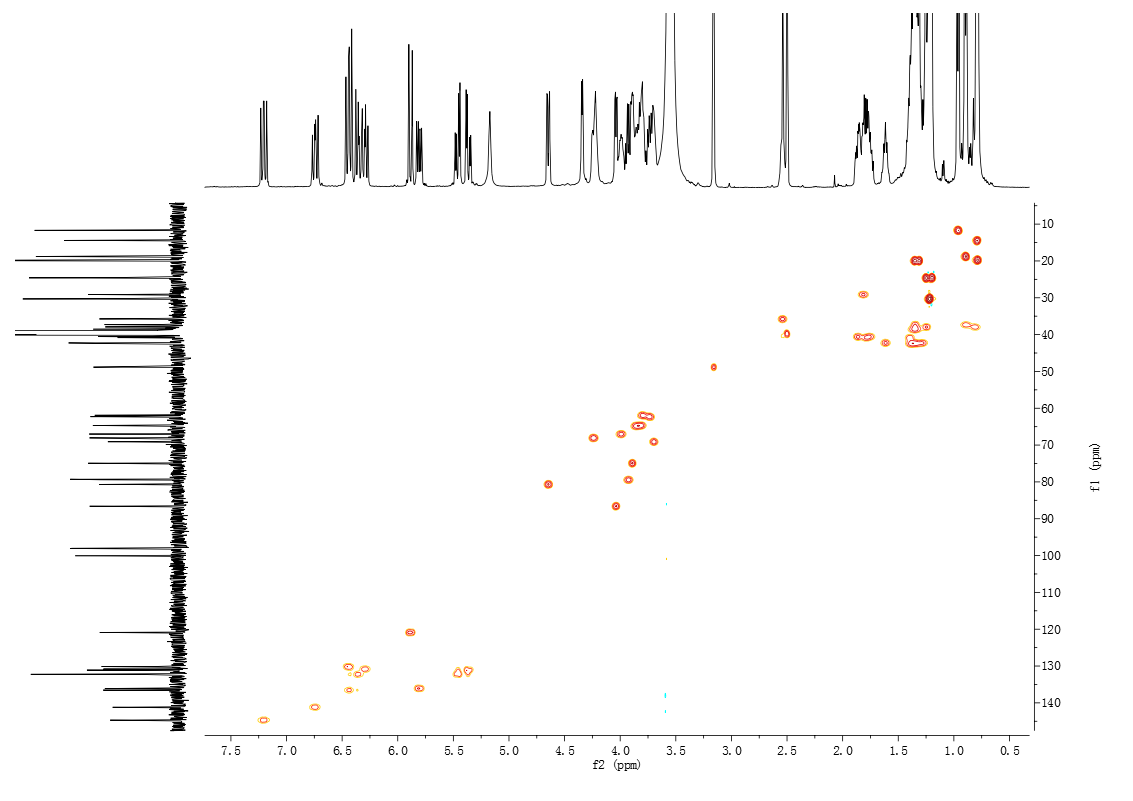


Figure S_36_. HMBC spectrum of compound **4a**


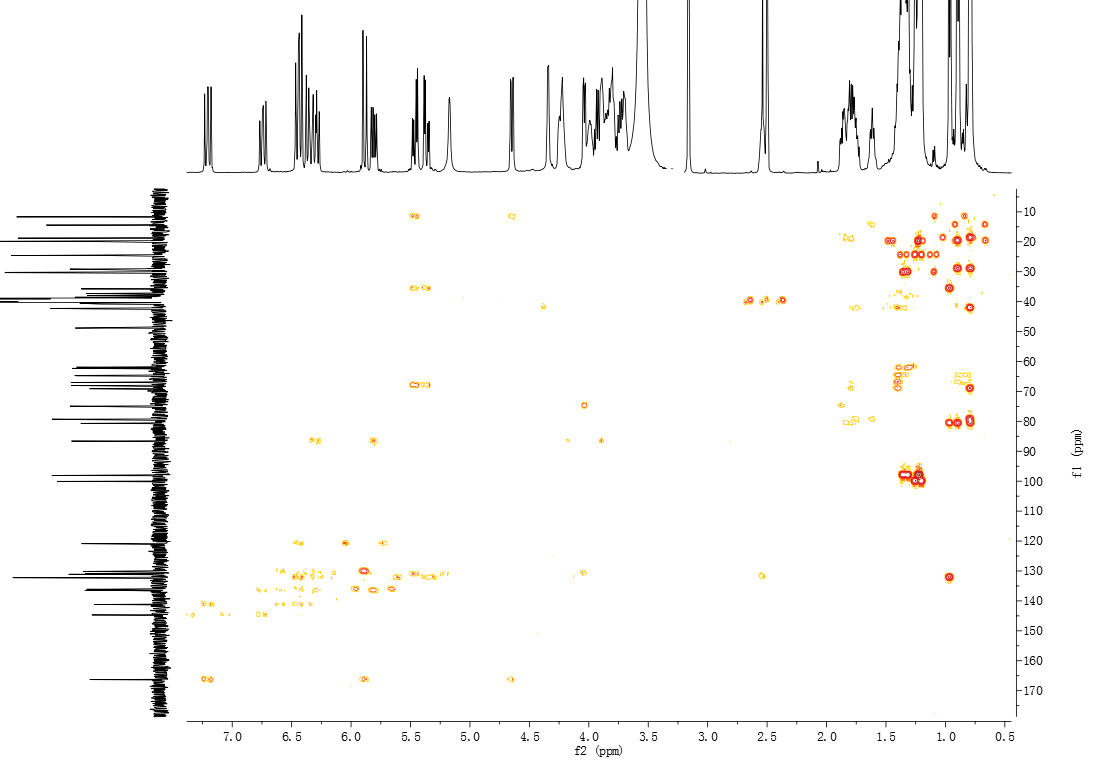


Figure S_37_. NOESY spectrum of compound **4a**


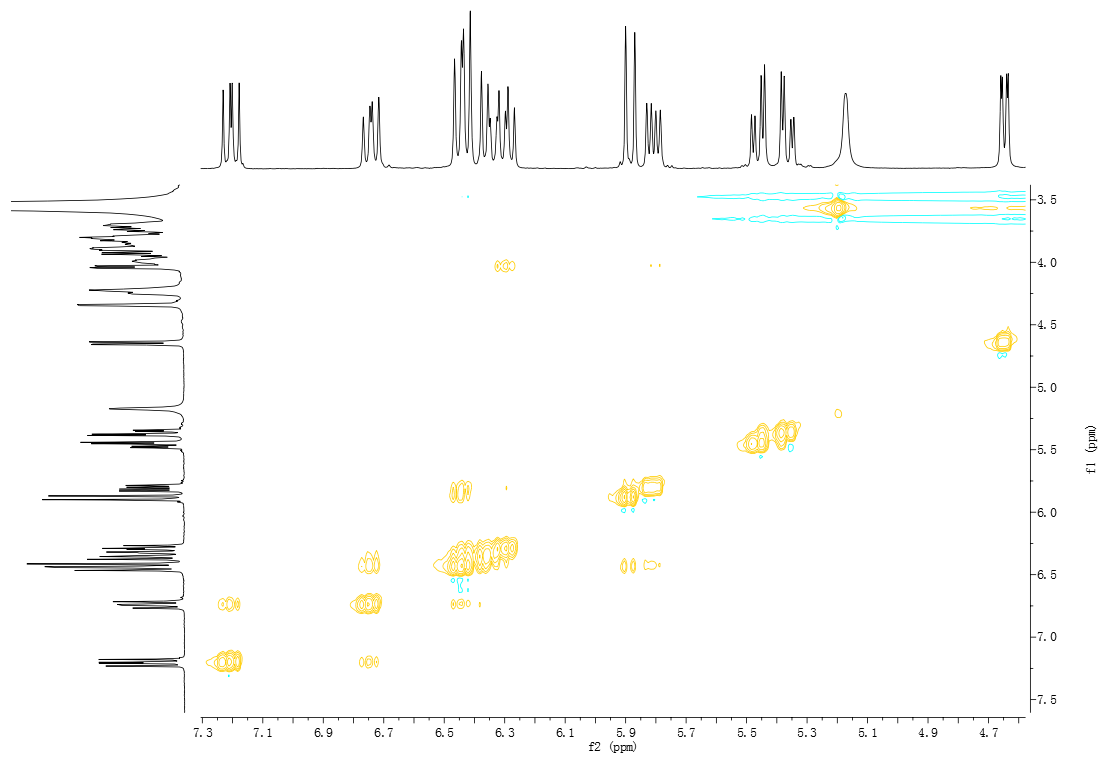


Figure S_38_. NOESY spectrum of compound **4a**


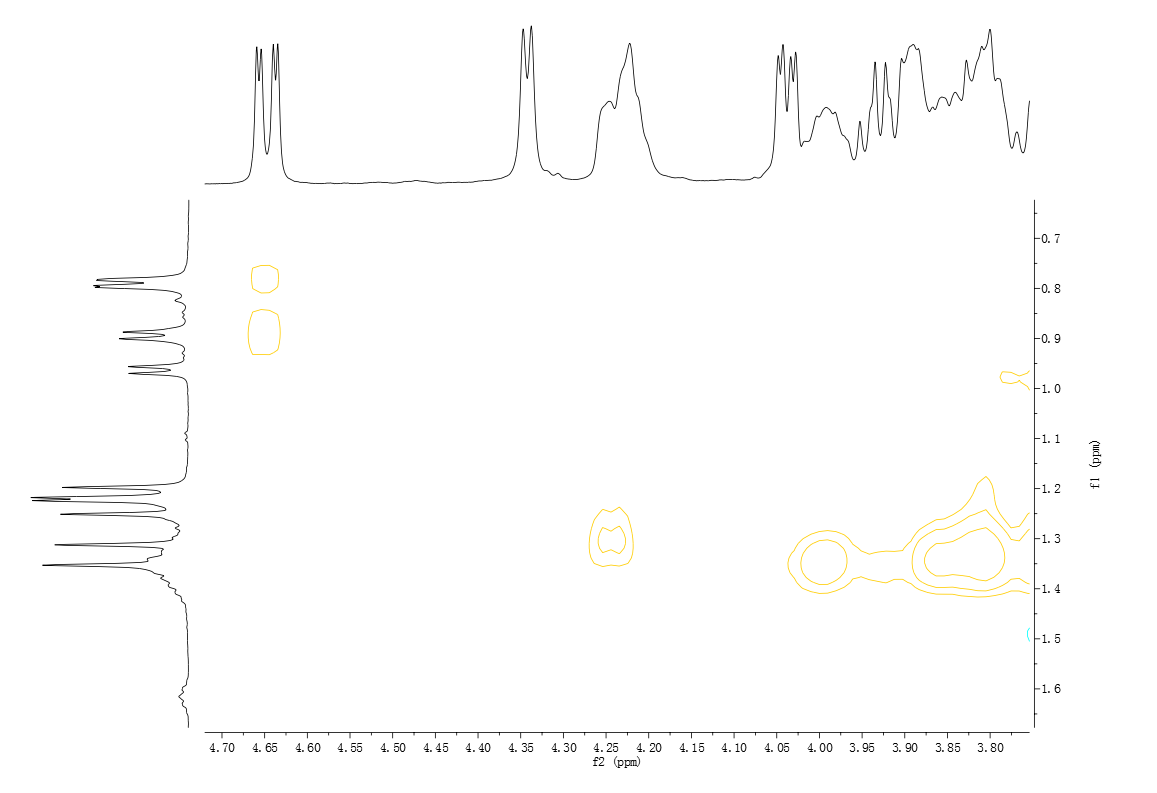


Figure S_39_. NOESY spectrum of compound **4a**


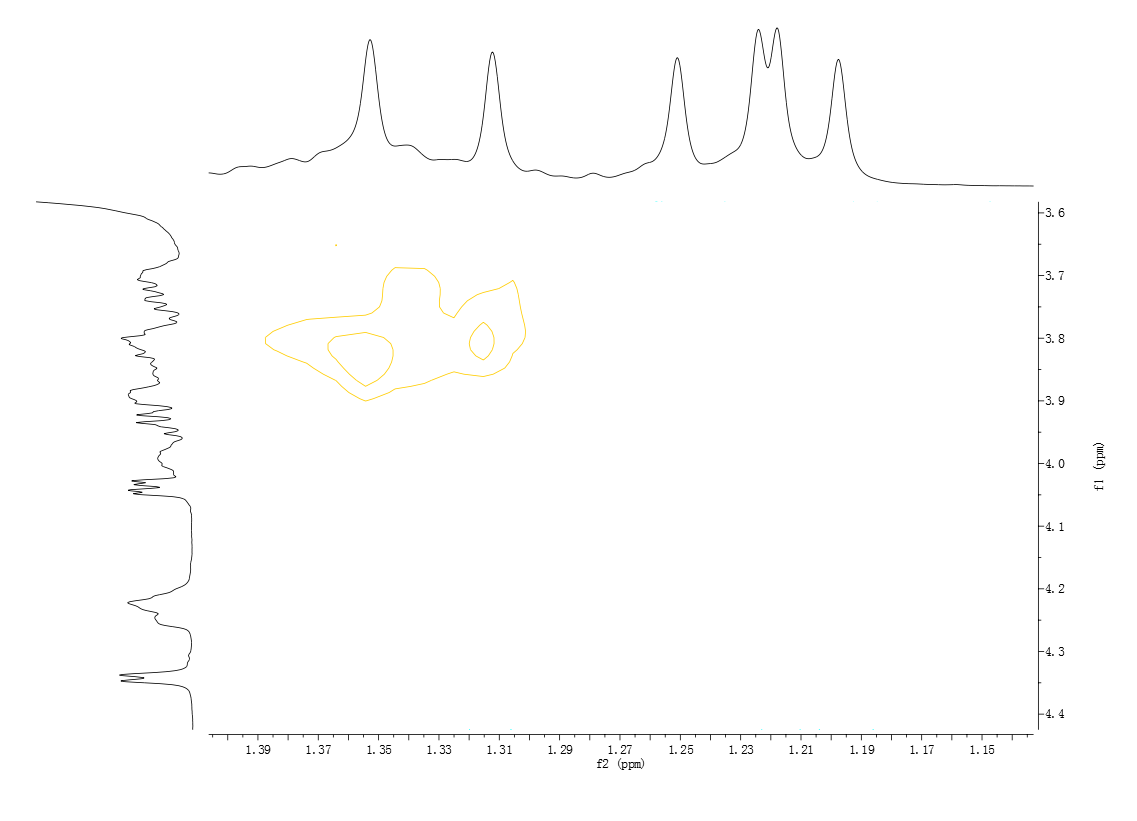


Figure S_40_. HRESIMS of compound **4a**

Figure S_41_. ^1^H-NMR spectrum of compound **4R**


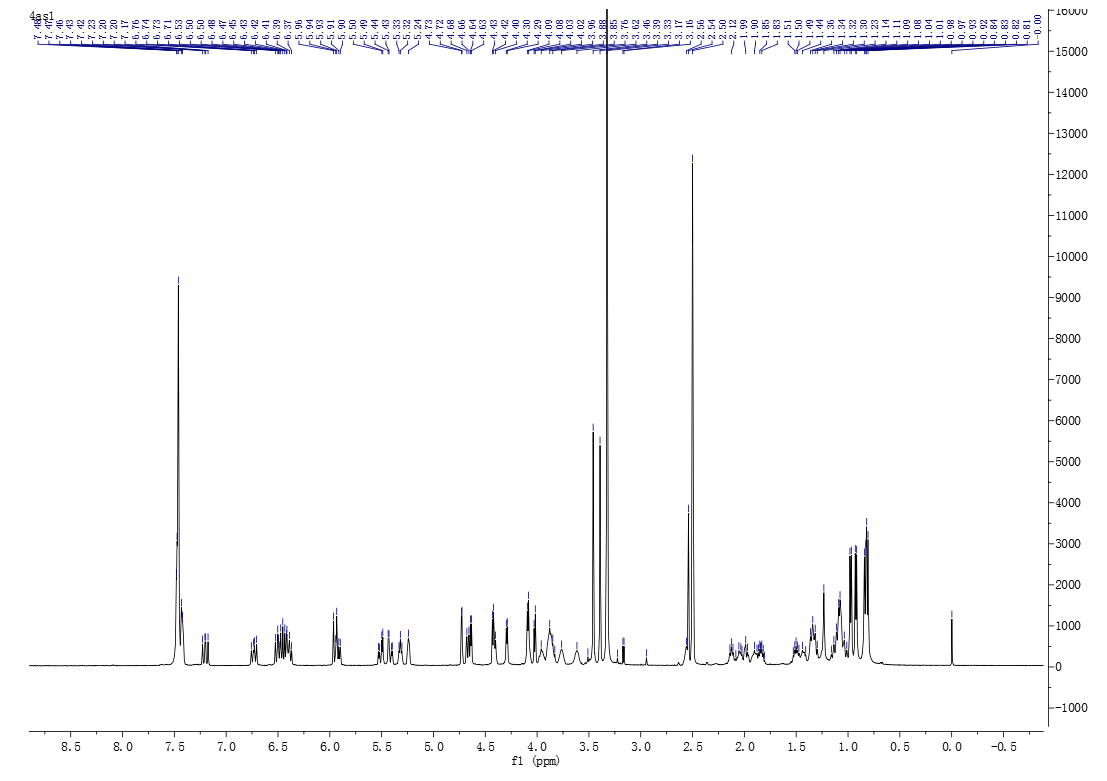


Figure S_42_. HSQC spectrum of compound **4R**

**
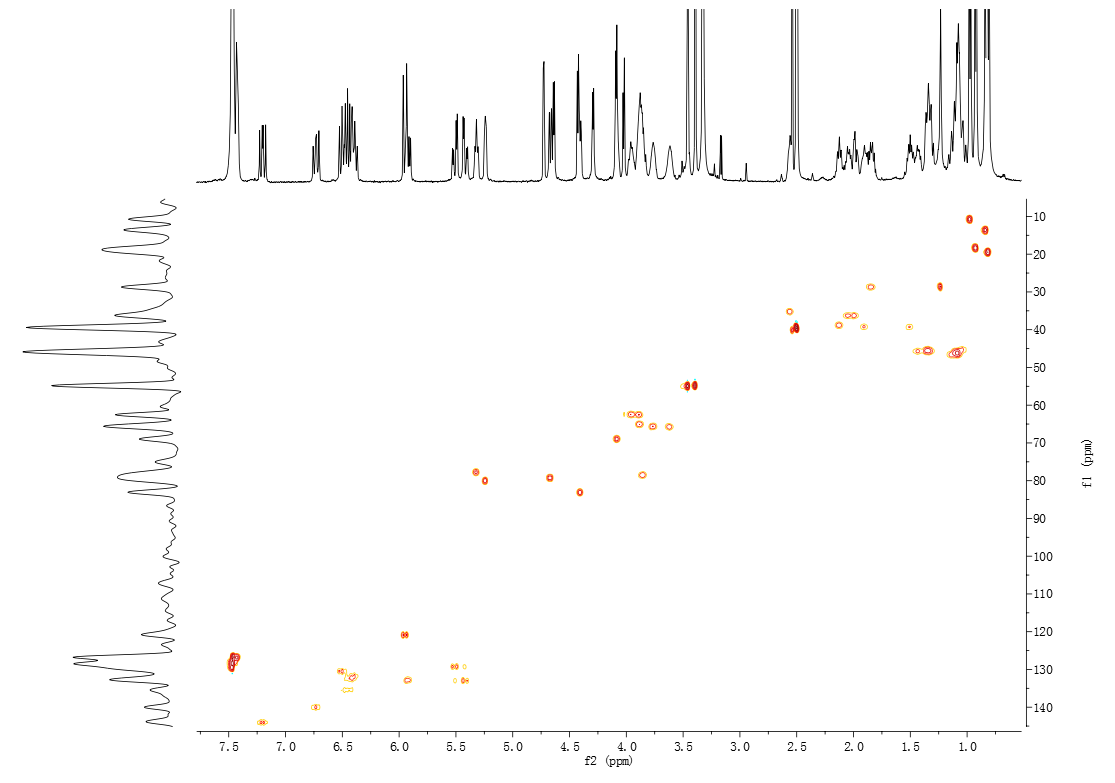
**

Figure S_43_. ^1^H-^1^H COSY spectrum of compound **4R**


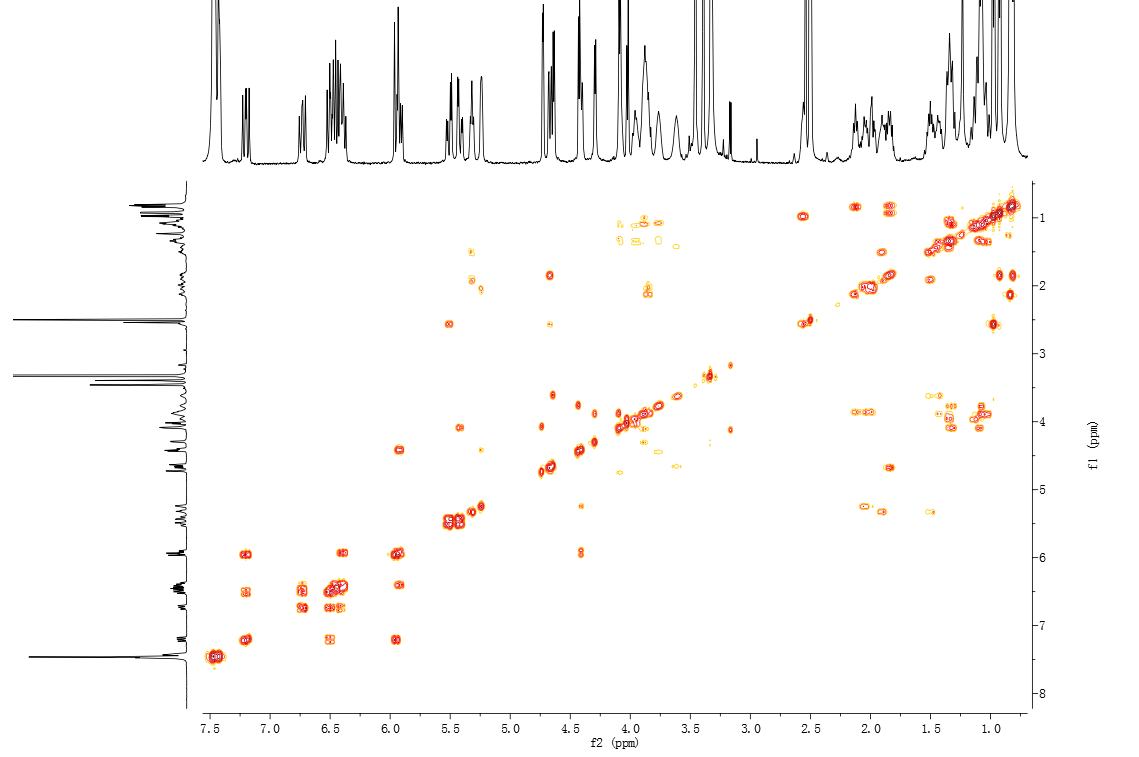


Figure S_44_. HRESIMS of compound **4R**

Figure S_45_. ^1^H-NMR spectrum of compound **4S**


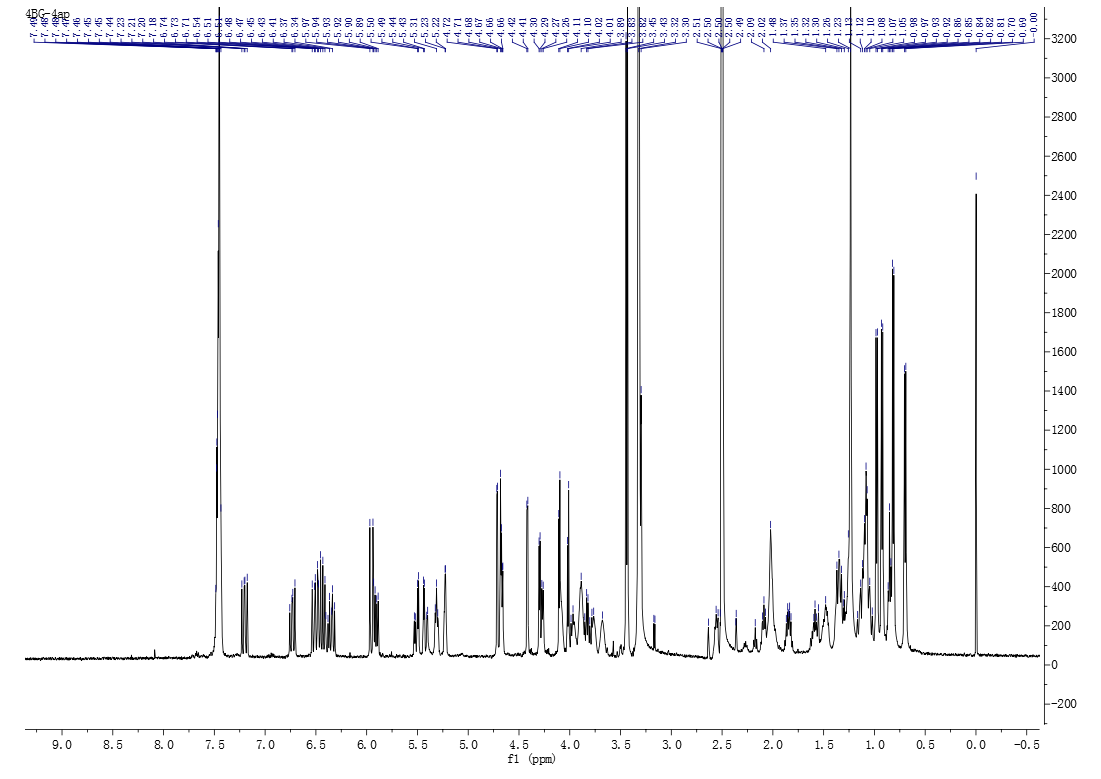


Figure S_46_. HSQC spectrum of compound **4S**


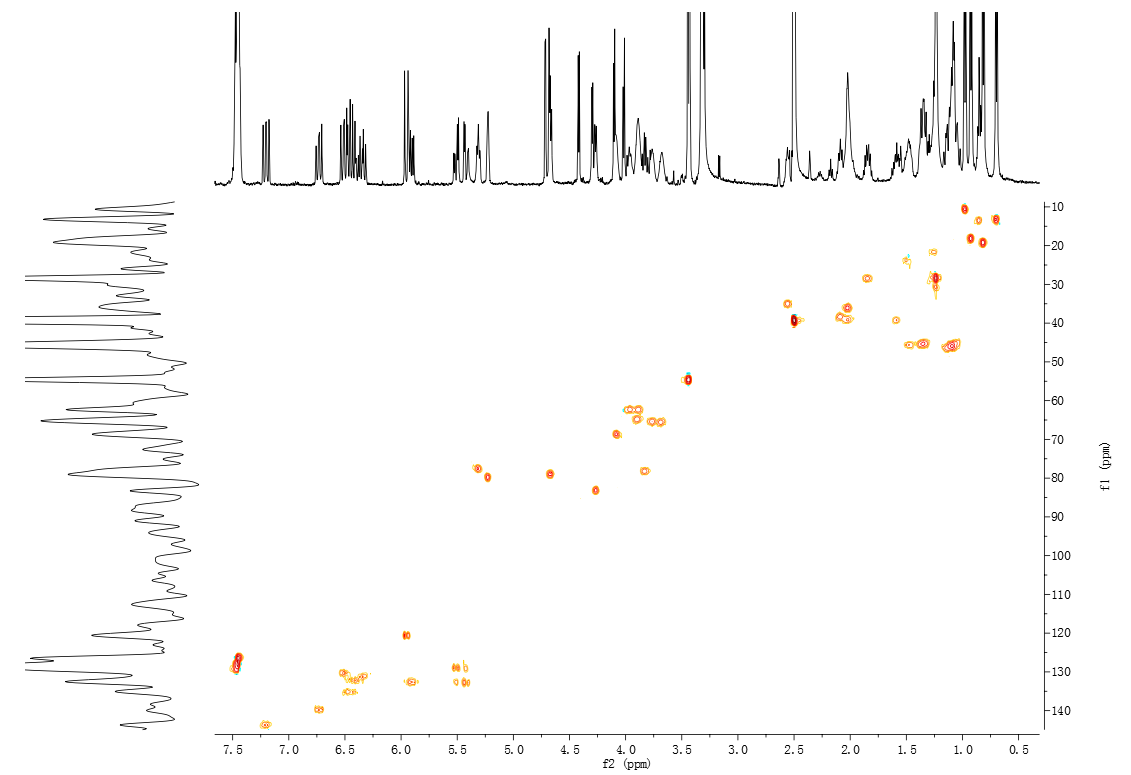


Figure S_47_. ^1^H-^1^H COSY spectrum of compound **4S**


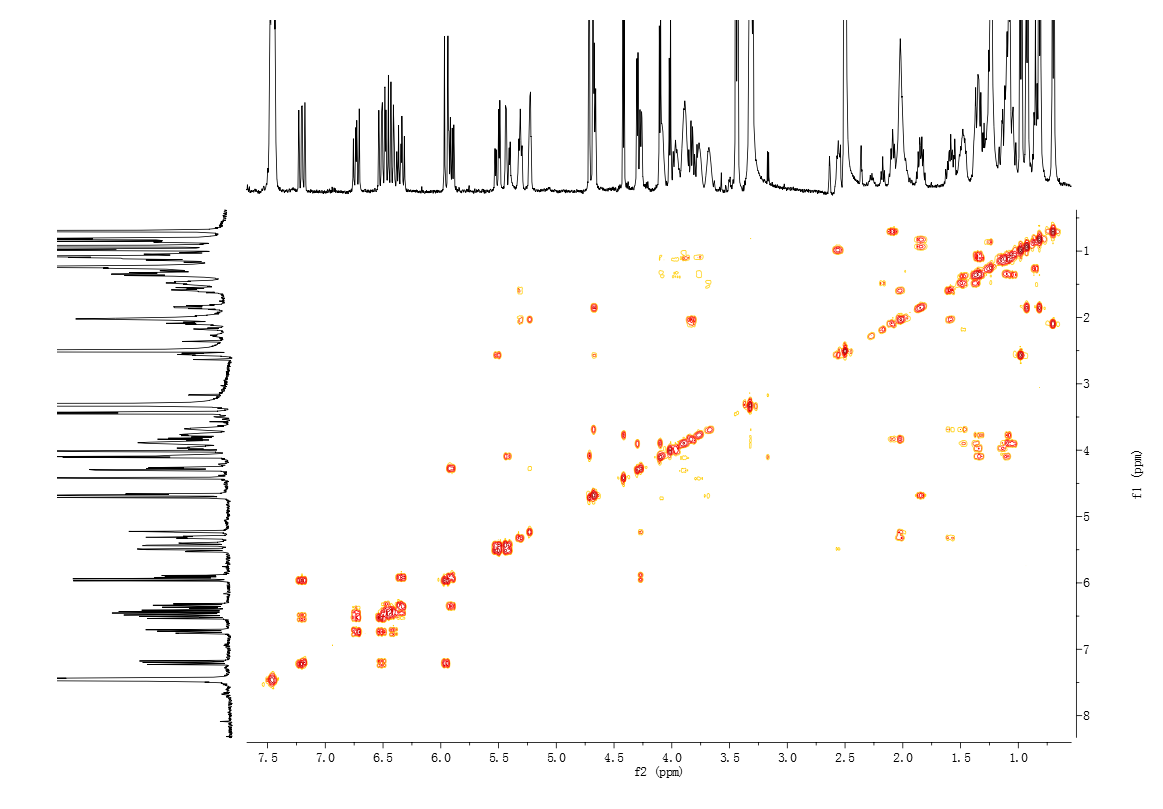


Figure S_48_. HRESIMS of compound **4S**


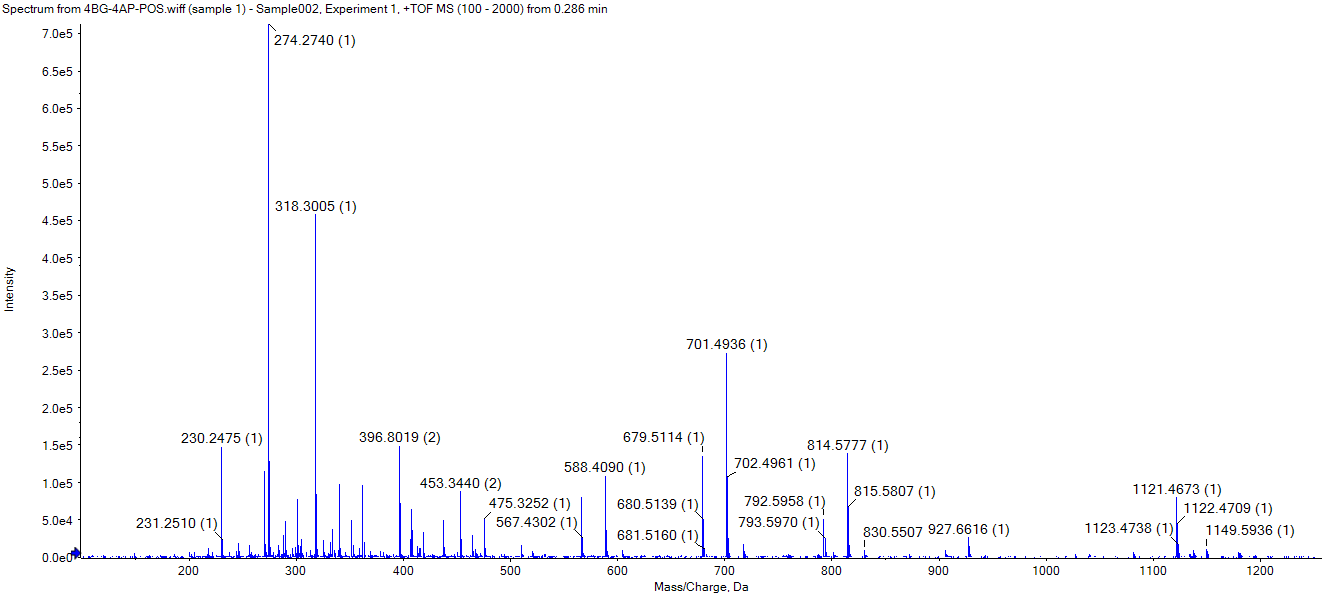


Figure S_49_. ^1^H-NMR spectrum of flavofungin V (**5**)


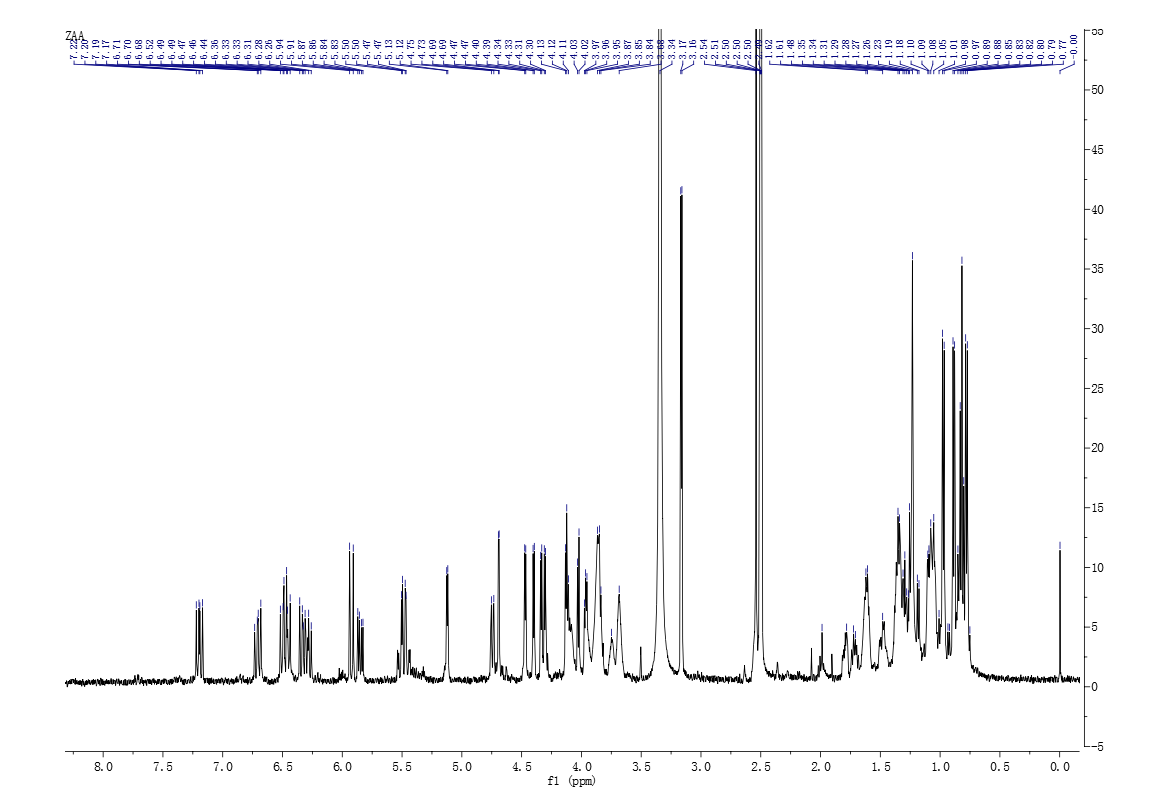


Figure S_50_. ^13^C-NMR spectrum of flavofungin V (**5**)


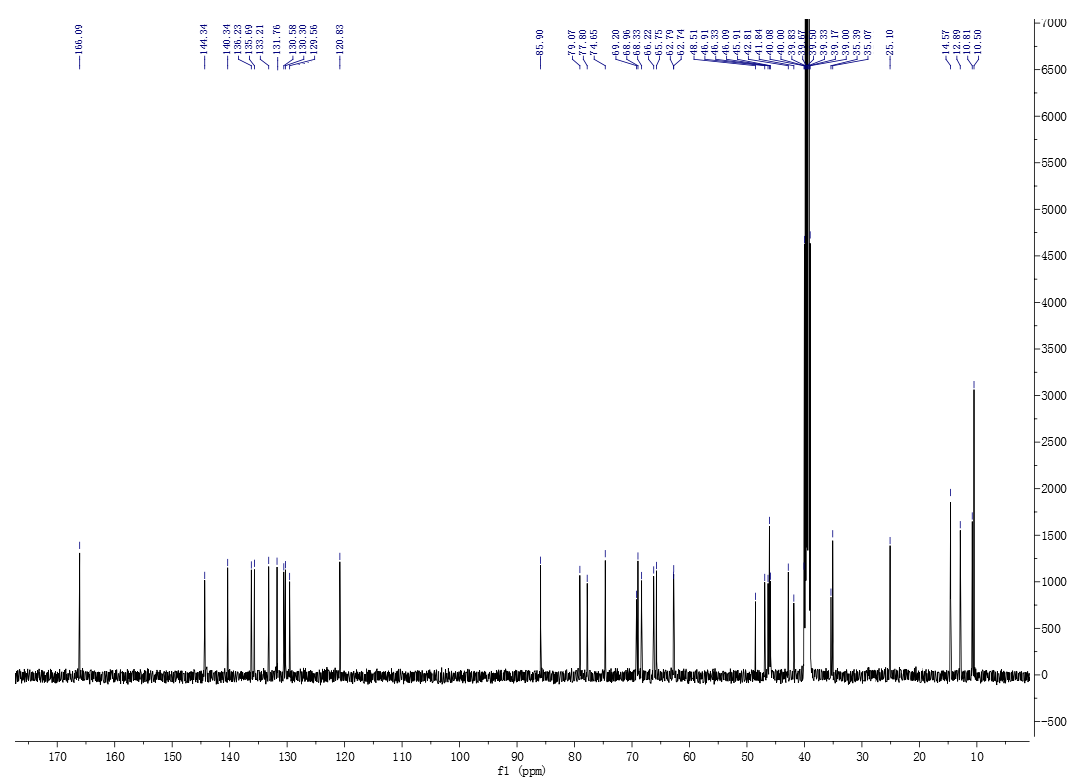


Figure S_51_. ^1^H-^1^H COSY spectrum of flavofungin V (**5**)


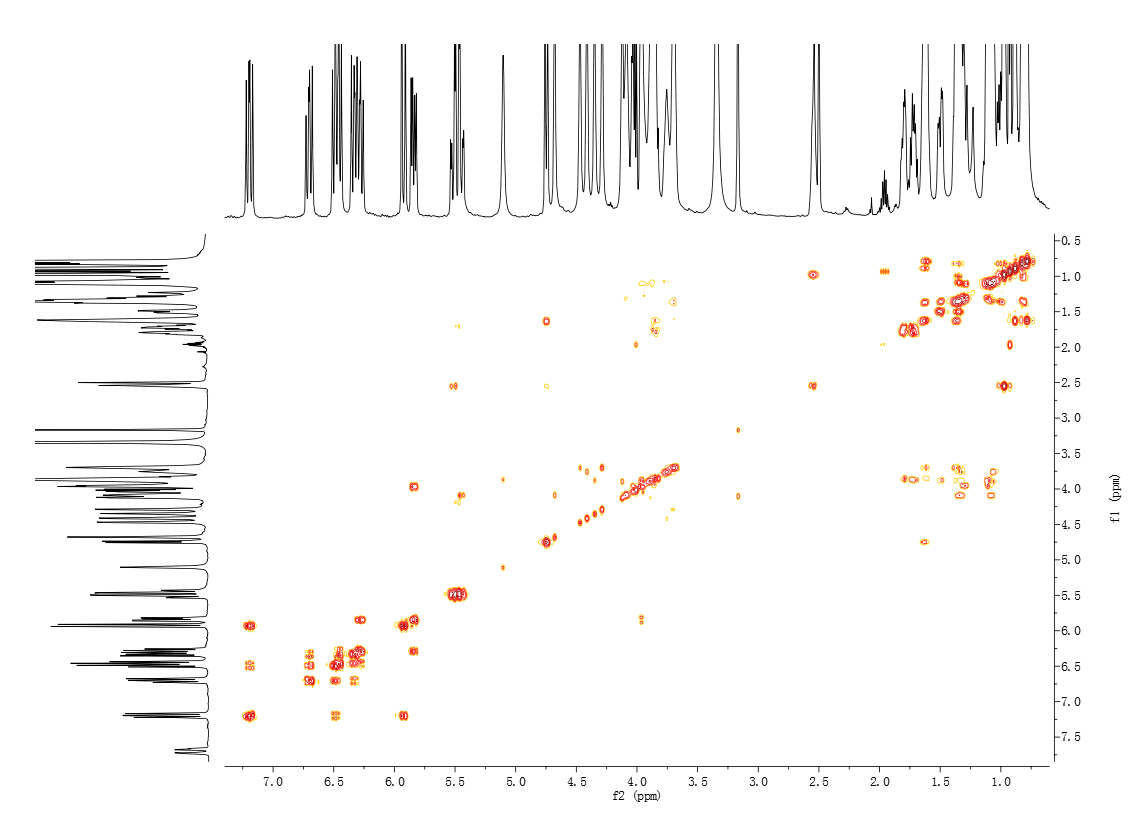


Figure S_52_. HSQC spectrum of flavofungin V (**5**)


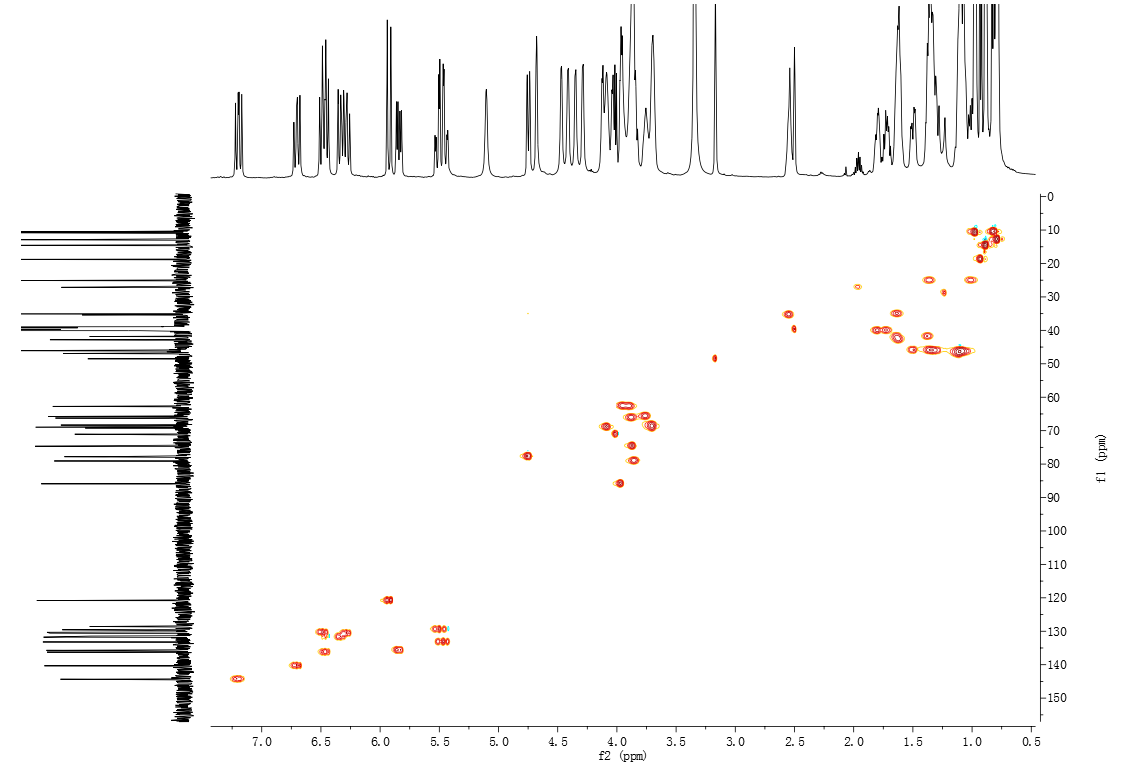


Figure S_53_. HMBC spectrum of flavofungin V (**5**)


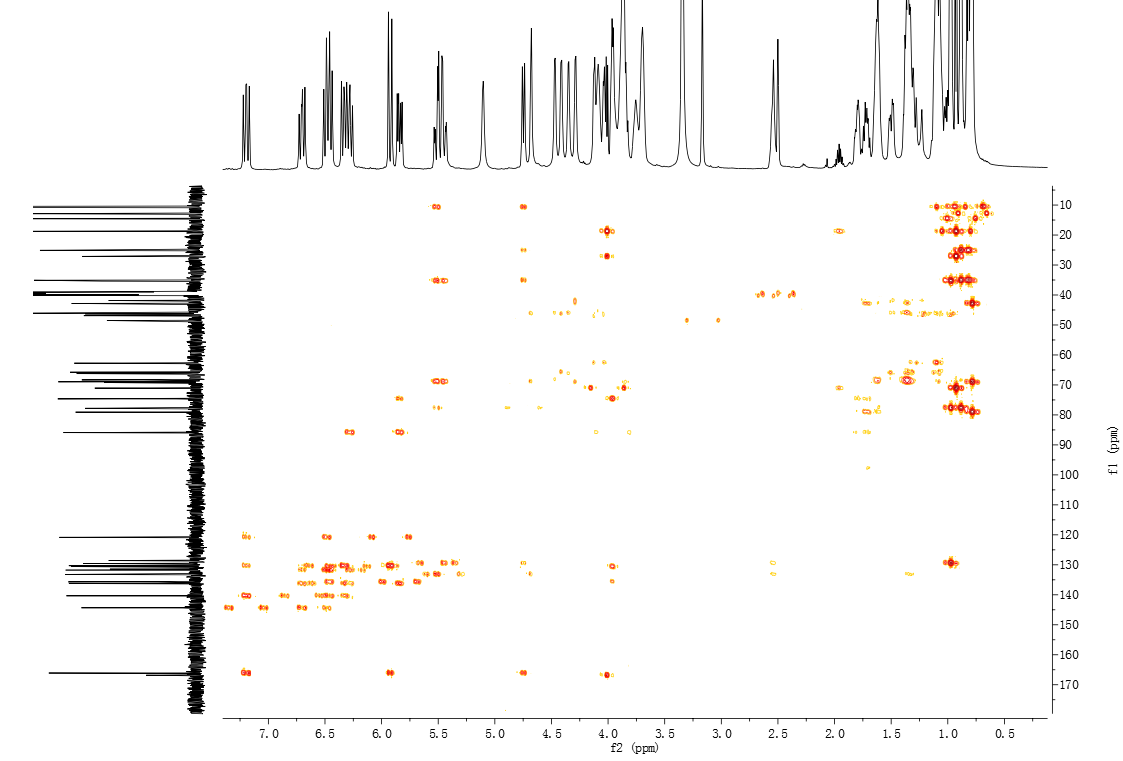


Figure S_54_. HRESIMS of flavofungin V (**5**)

Figure S_55_. ^1^H-NMR spectrum of compound **5a**


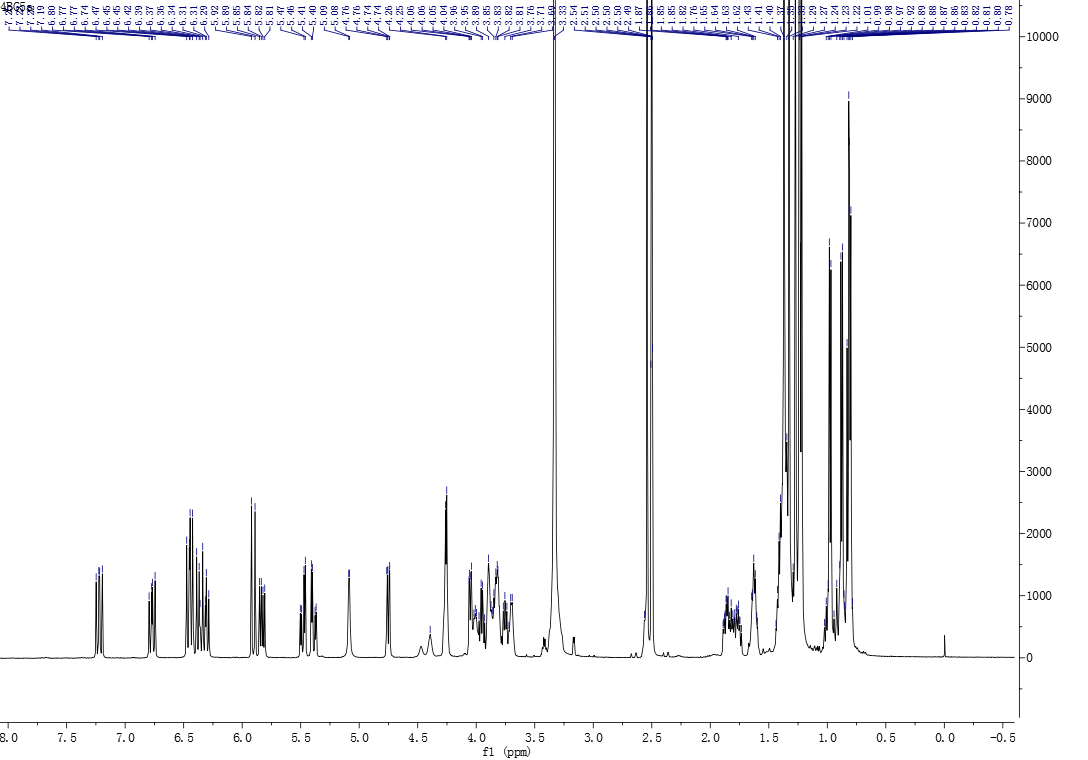


Figure S_56_. ^13^C-NMR spectrum of compound **5a**


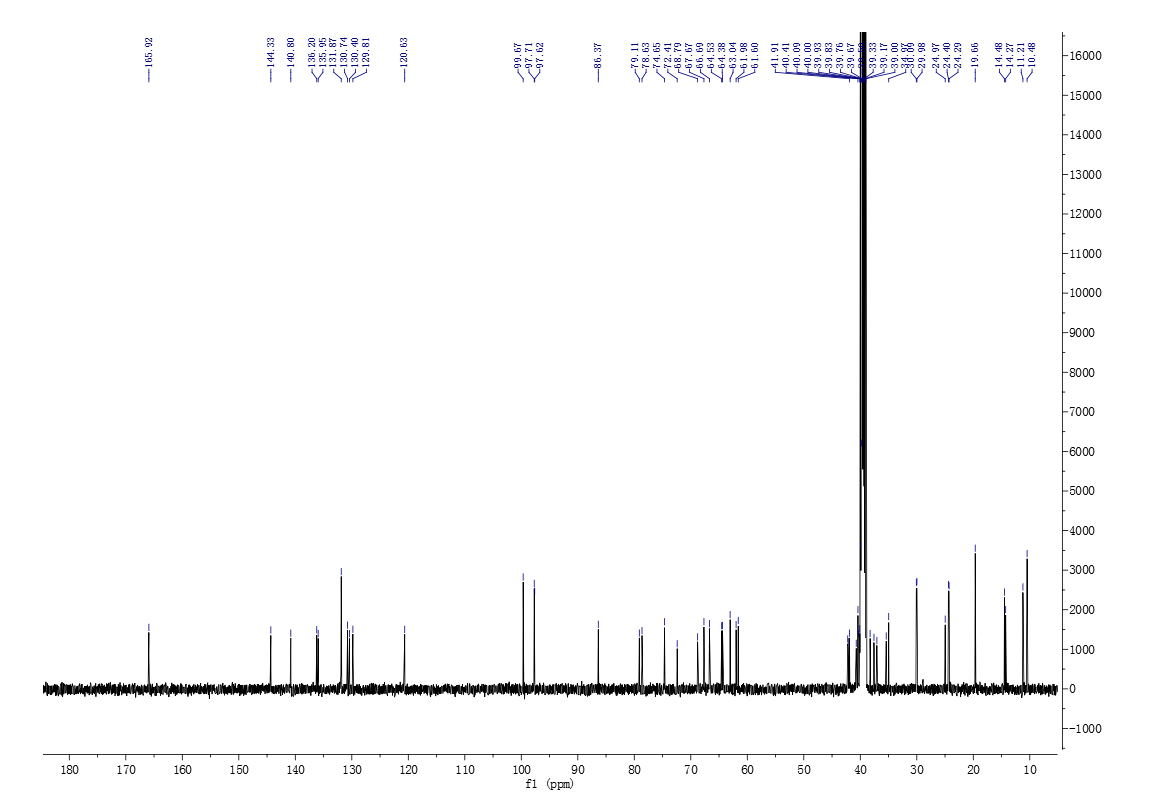


Figure S_57_. HSQC spectrum of compound **5a**


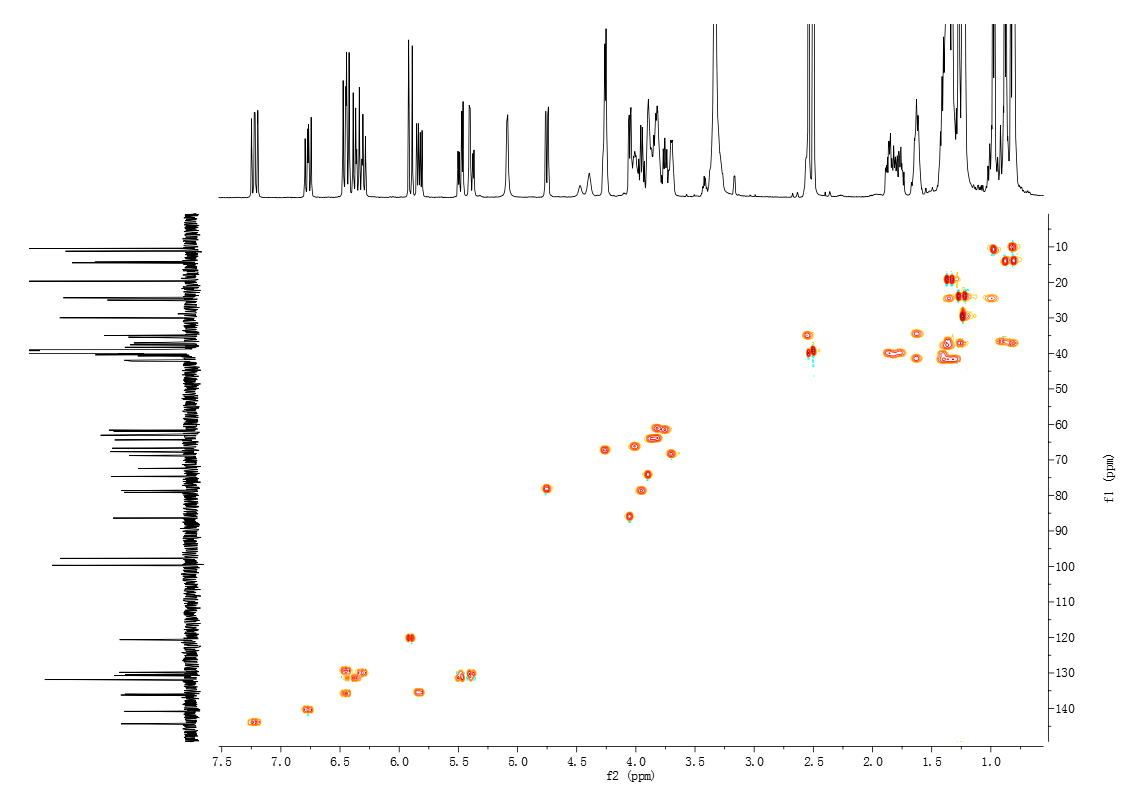


Figure S_58_. ^1^H-^1^H COSY spectrum of compound **5a**


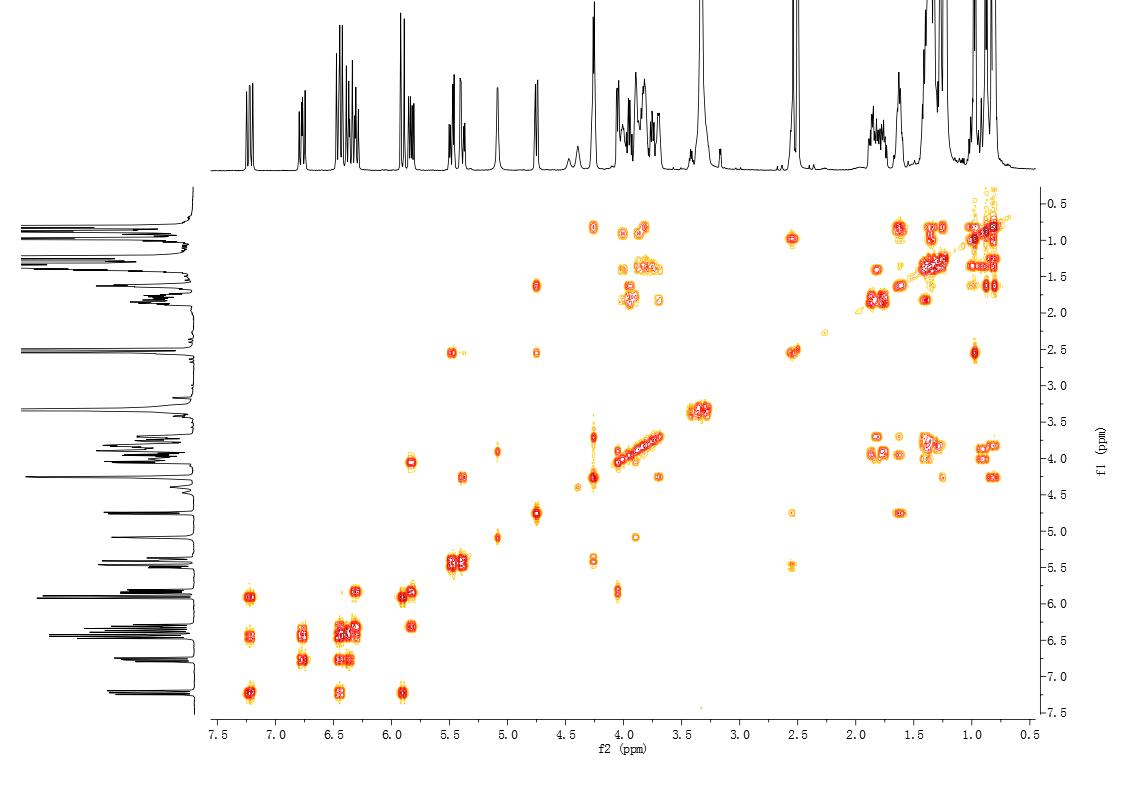


Figure S_59_. NOESY spectrum of compound **5a**


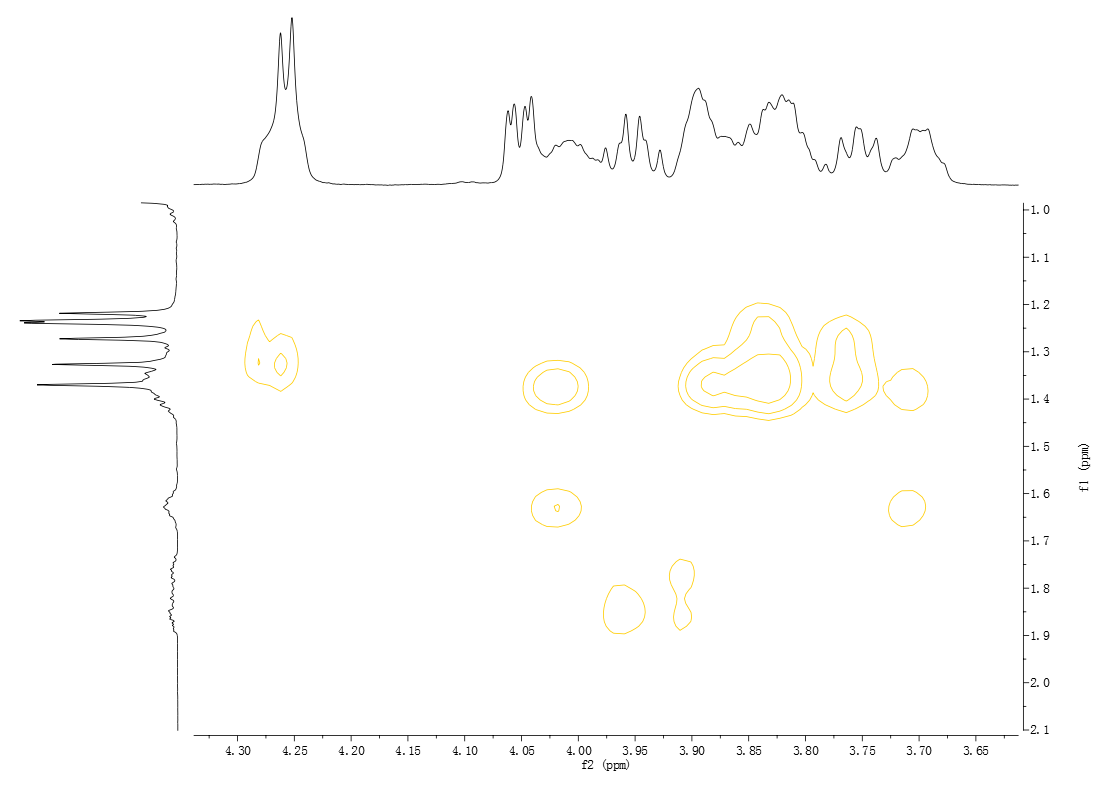


Figure S_60_. HRESIMS of compound **5a**


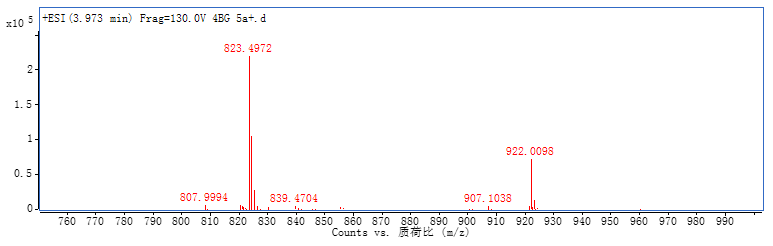


Figure S_61_. ^1^H-NMR spectrum of flavofungin VI (**6**)


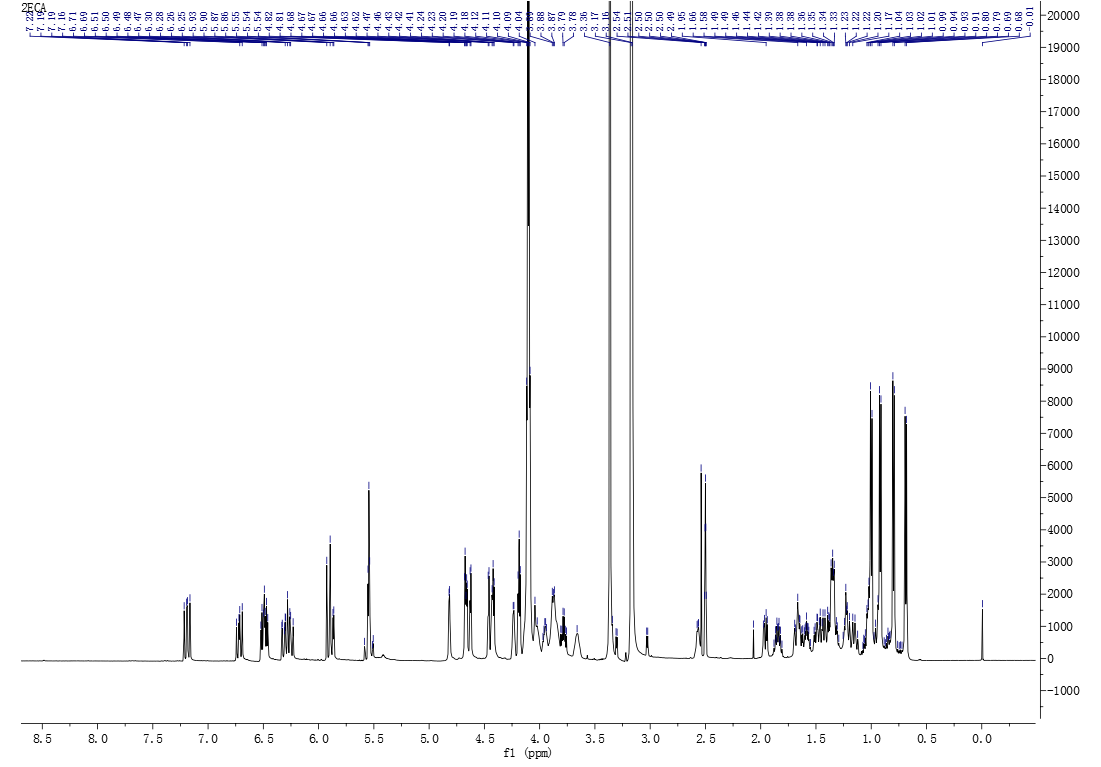


Figure S_62_. ^13^C-NMR spectrum of flavofungin VI (**6**)


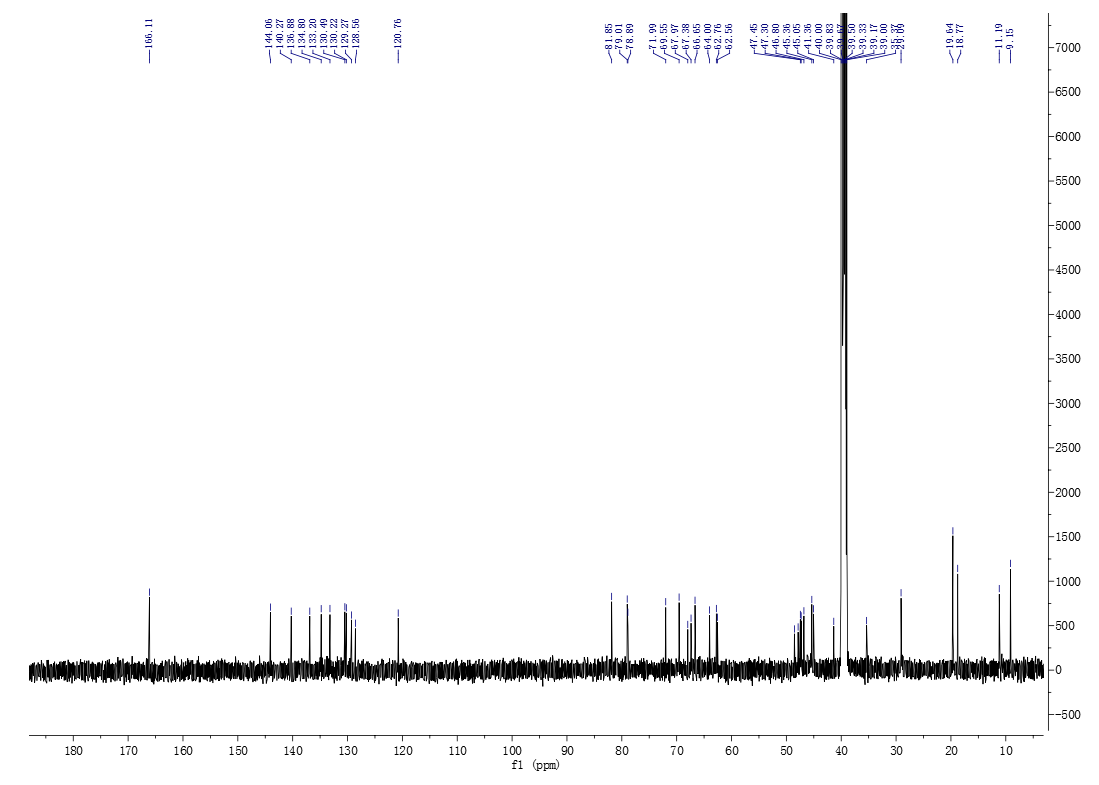


Figure S_63_. ^1^H-^1^H COSY spectrum of flavofungin VI (**6**)


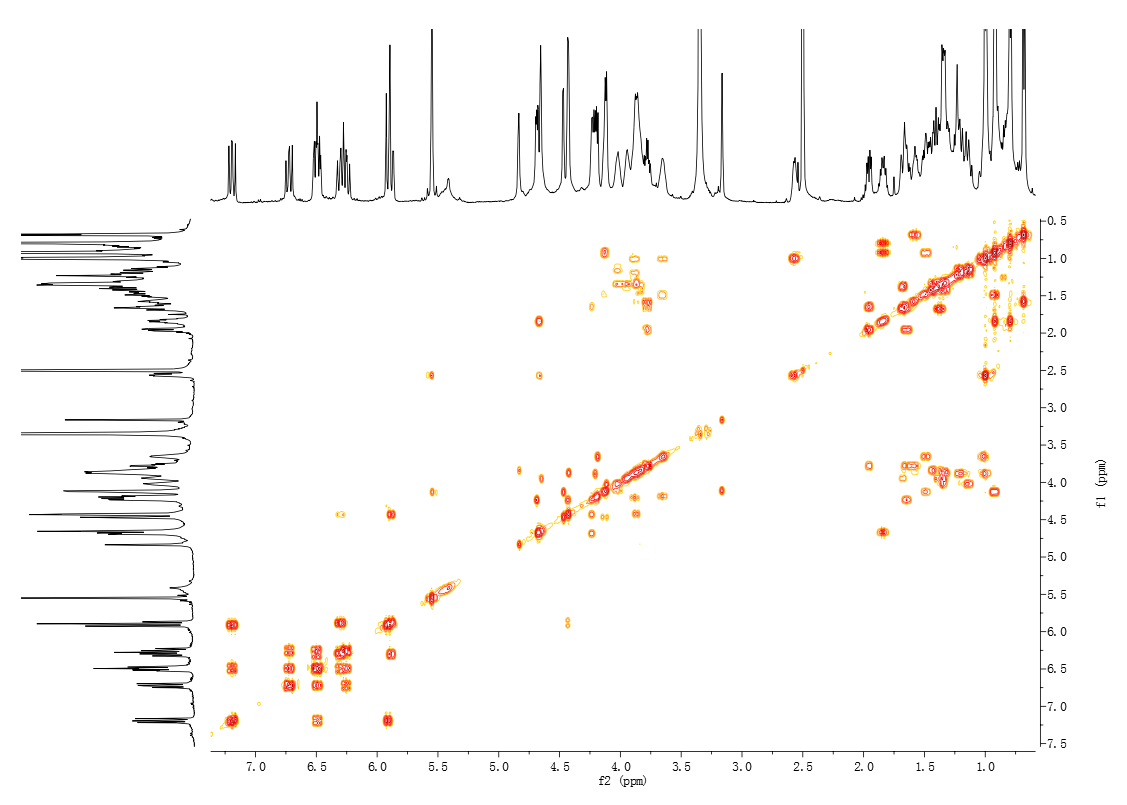


Figure S_64_. HSQC spectrum of flavofungin VI (**6**)


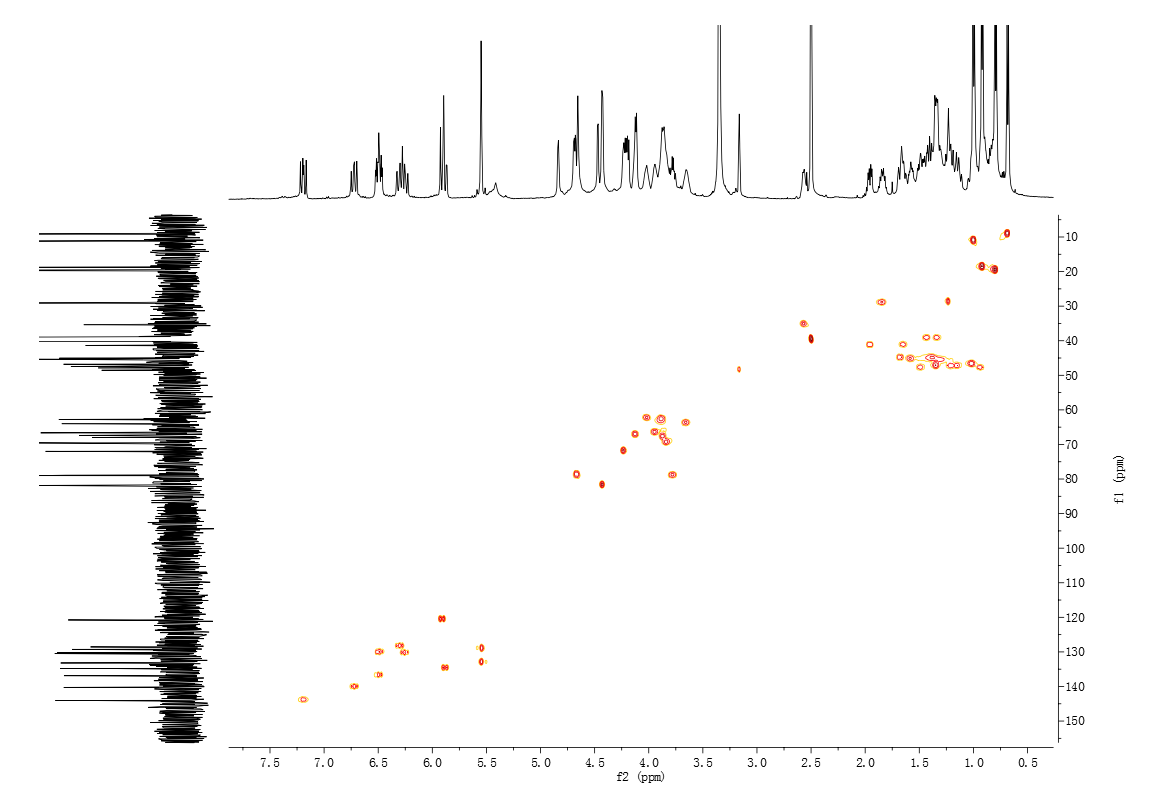


Figure S_65_. HMBC spectrum of flavofungin VI (**6**)


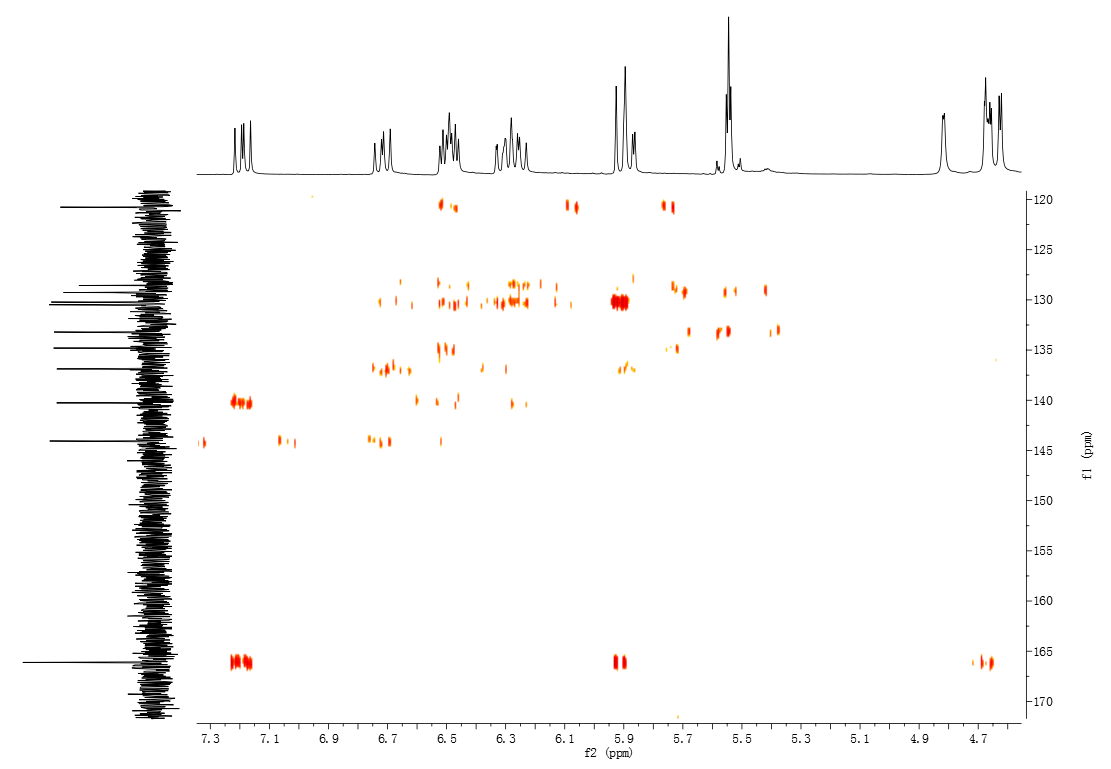


Figure S_66_. HRESIMS of flavofungin VI (**6**)

Figure S_67_. ^1^H-NMR spectrum of compound **6a**


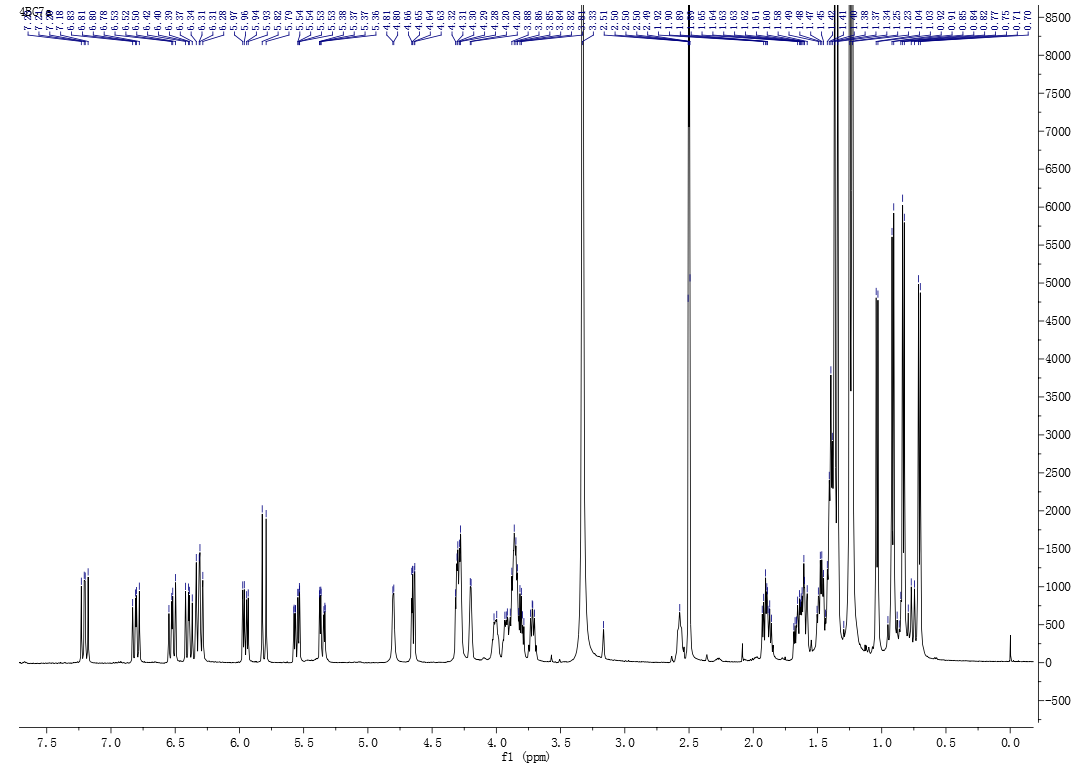


Figure S_68_. ^13^C-NMR spectrum of compound **6a**


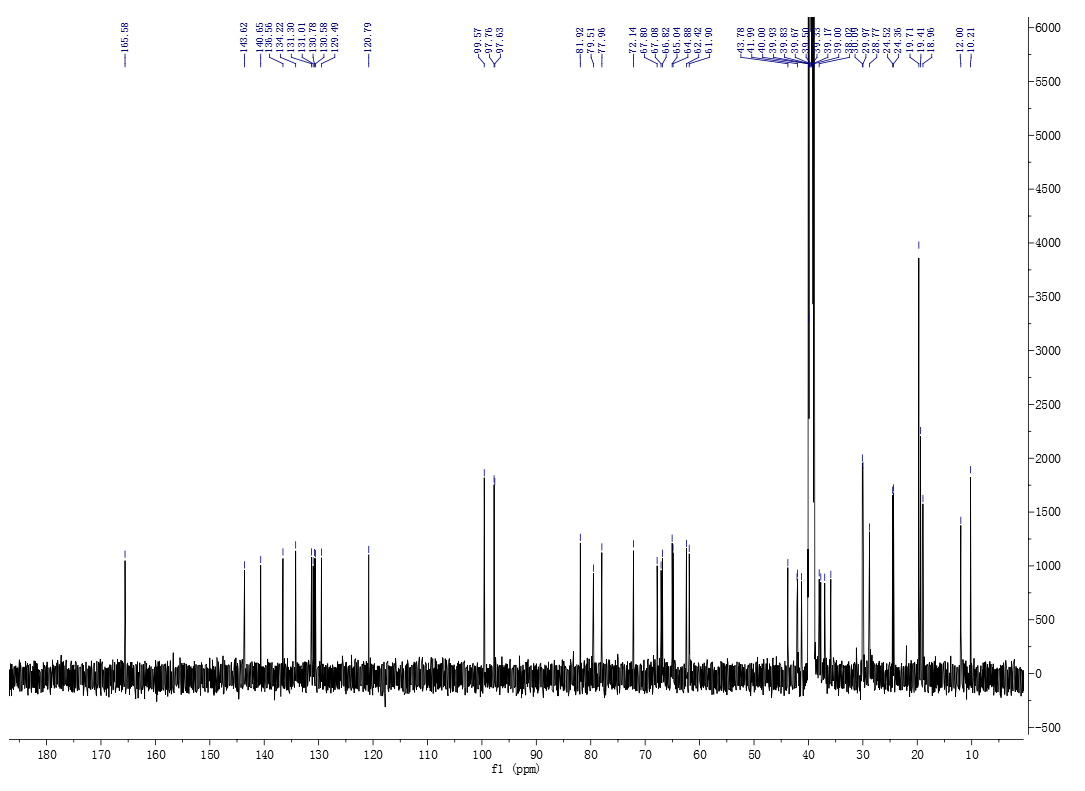


Figure S_69_. HSQC spectrum of compound **6a**


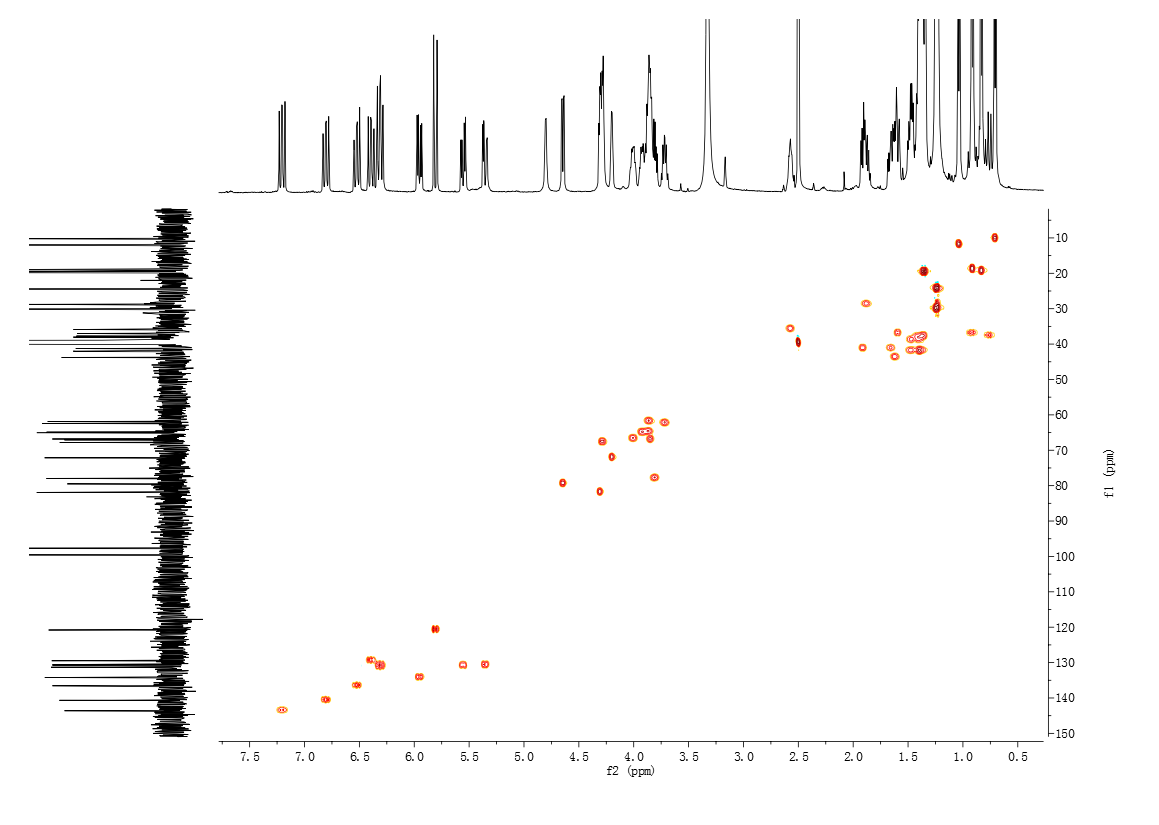


Figure S_70_. ^1^H-^1^H COSY spectrum of compound **6a**

**
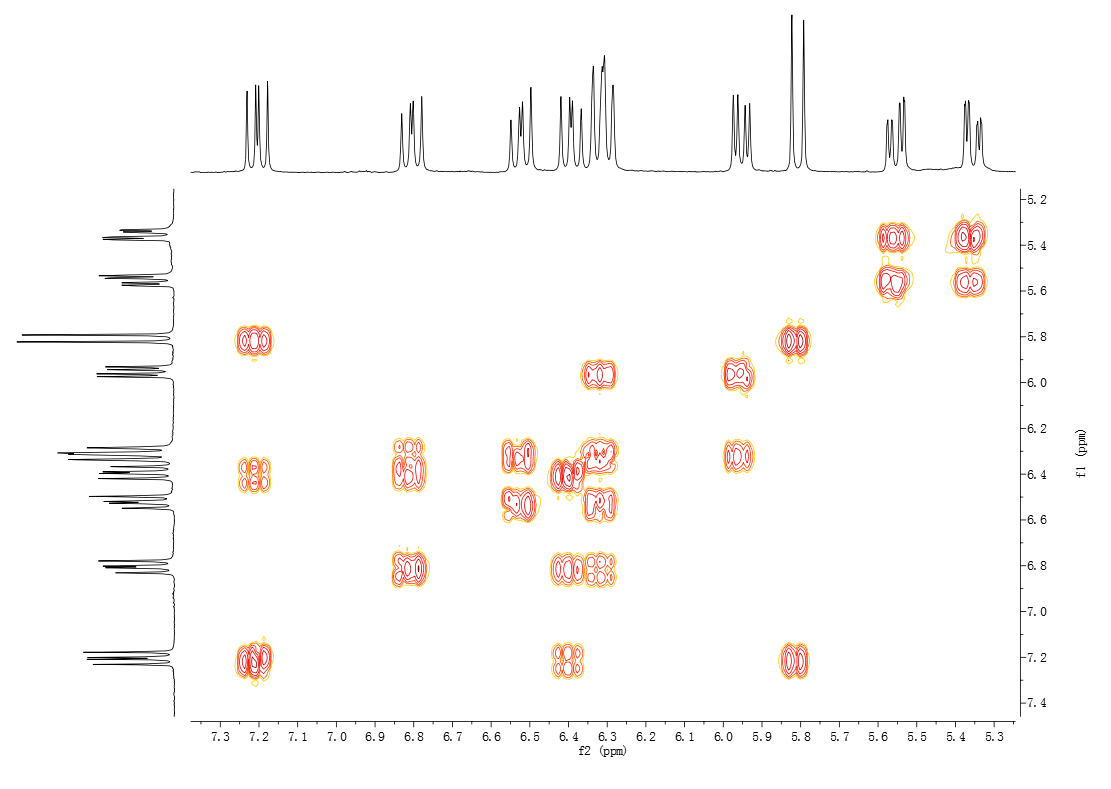
**

Figure S_71_. ^1^H-^1^H COSY spectrum of compound **6a**

**
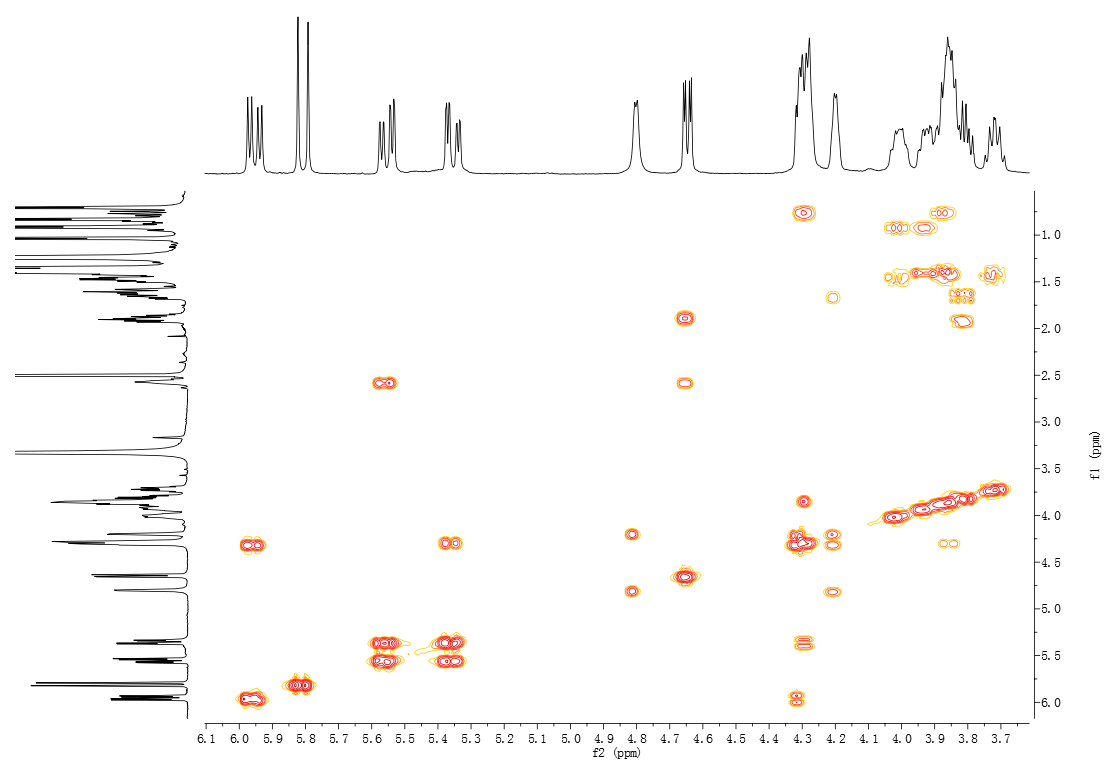
**

Figure S_72_. ^1^H-^1^H COSY spectrum of compound **6a**

**
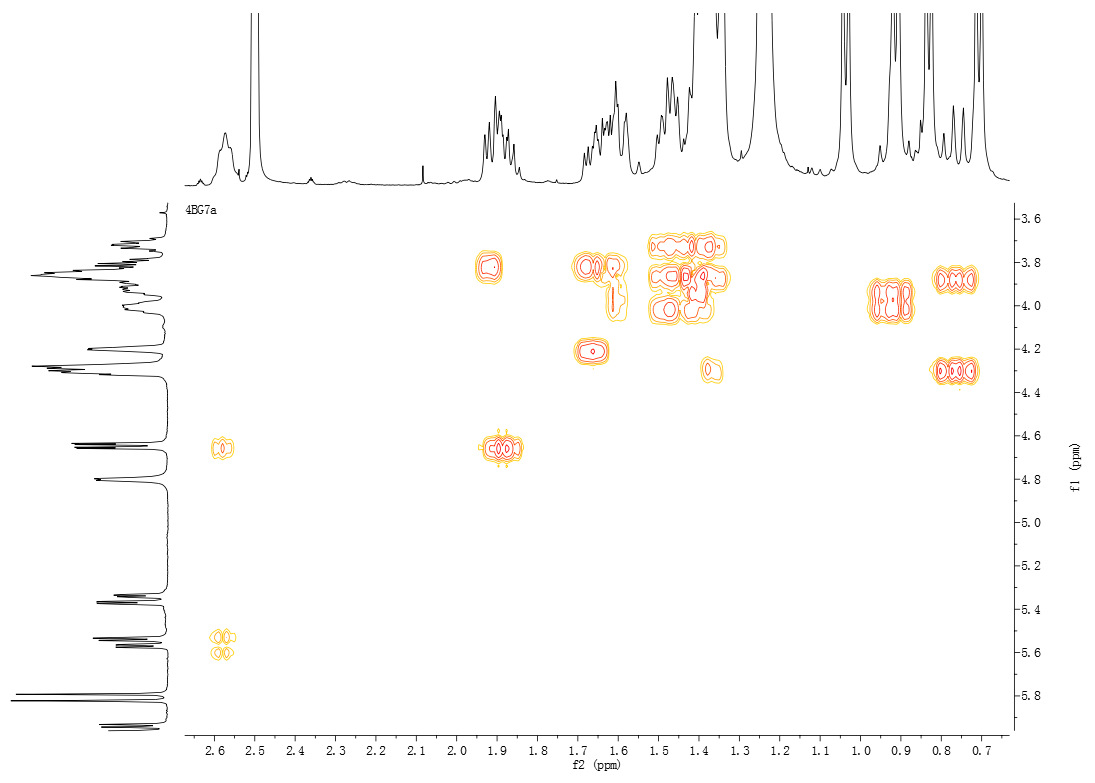
**

Figure S_73_. NOESY spectrum of compound **6a**

**
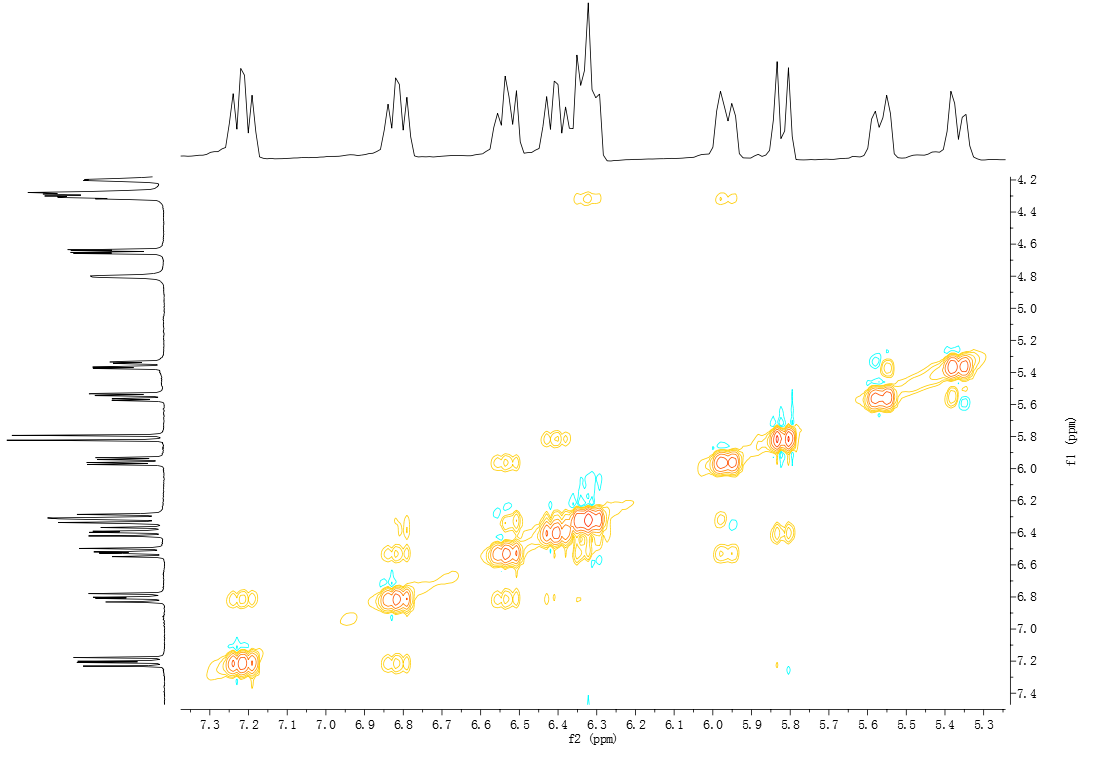
**

Figure S_74_. NOESY spectrum of compound **6a**

**
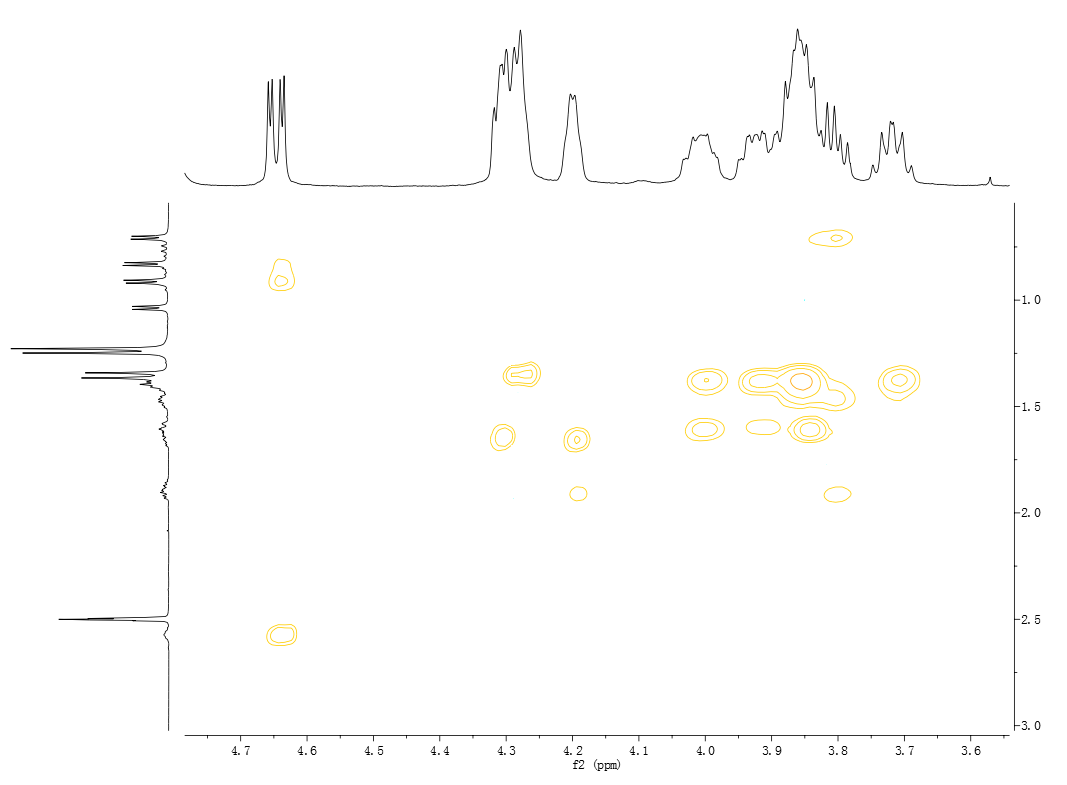
**

Figure S_75_. NOESY spectrum of compound **6a**

**
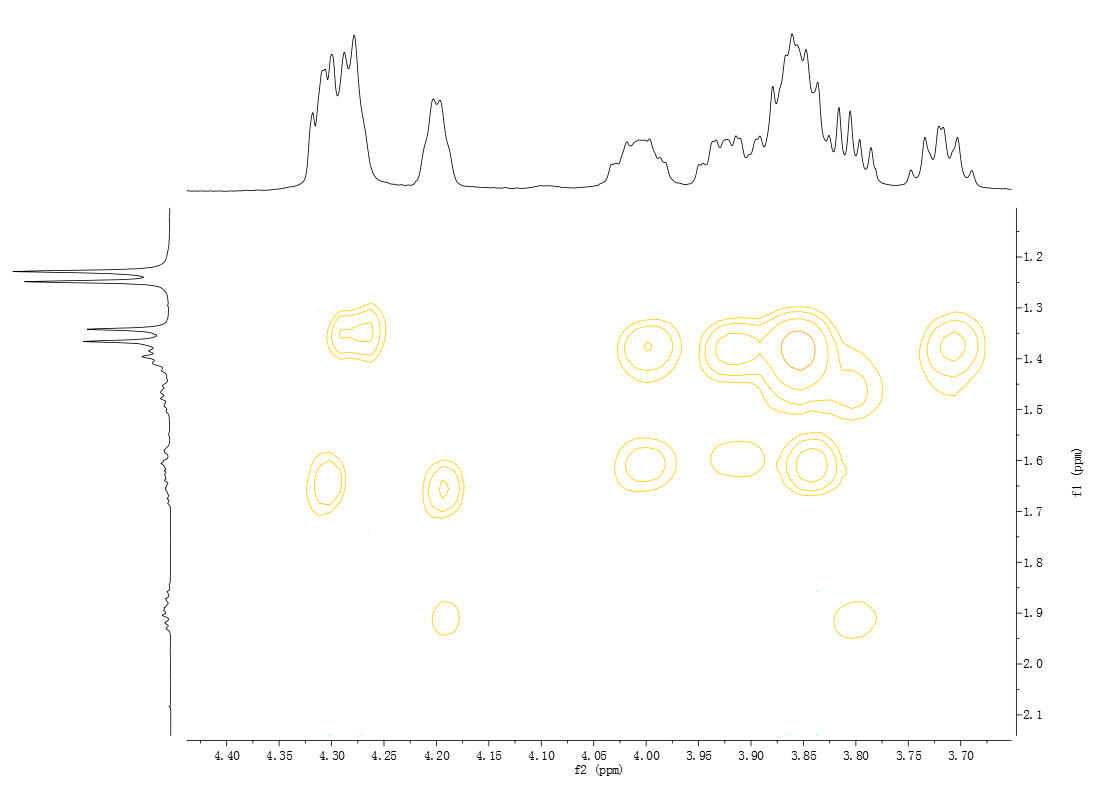
**

Figure S_76_. NOESY spectrum of compound **6a**

**
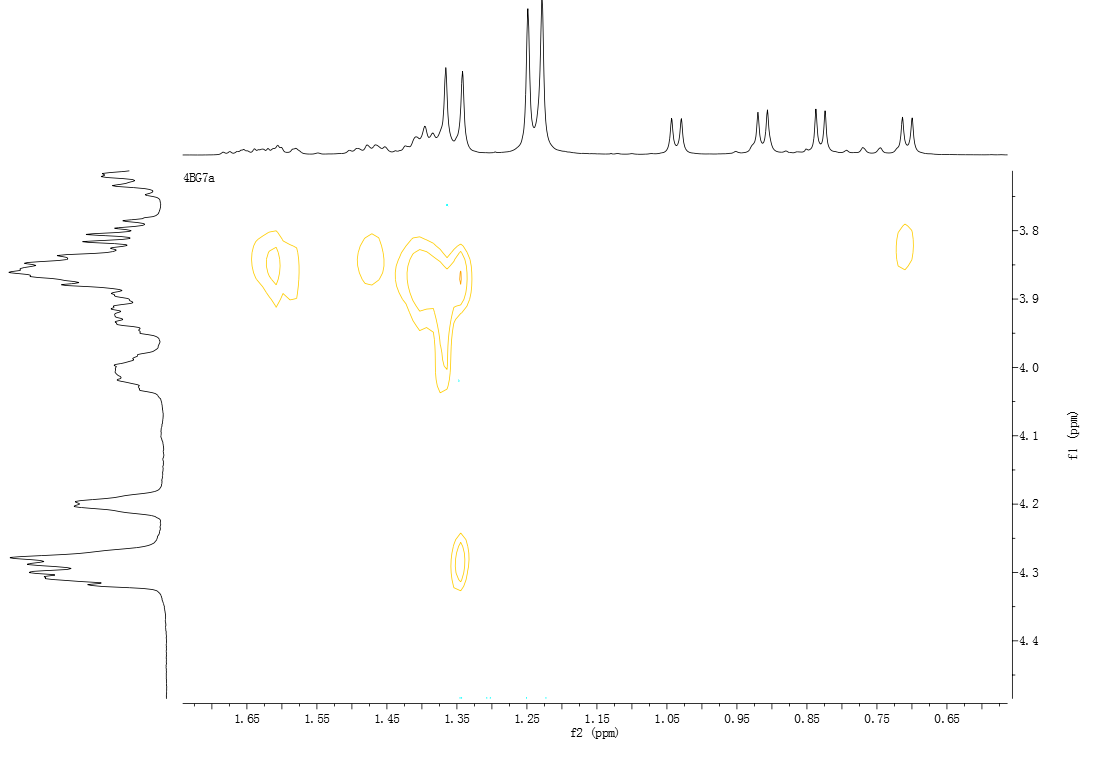
**

Figure S_77_. HRESIMS of compound **6a**


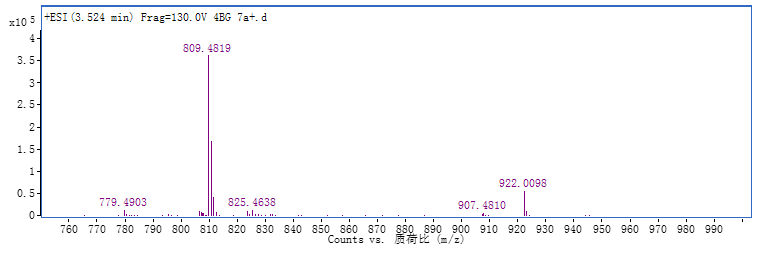


Figure S_78_. ^1^H-NMR spectrum of flavofungin VII (**7**)


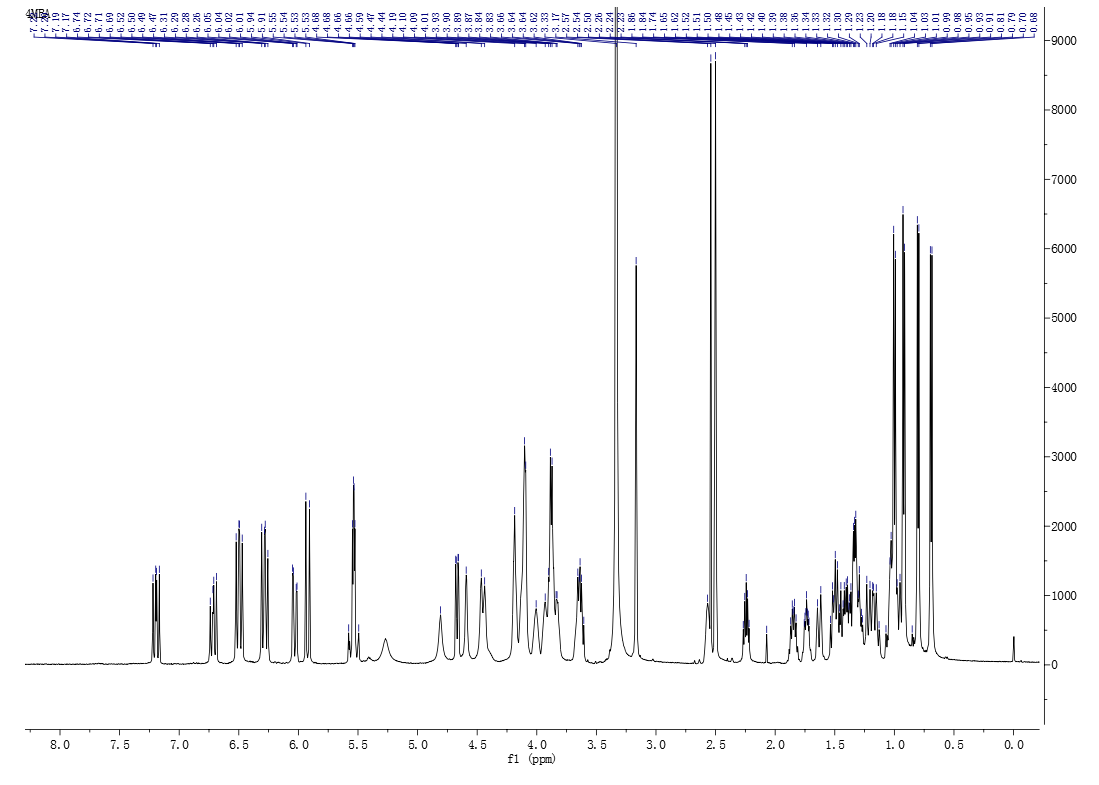


Figure S_79_. ^13^C-NMR spectrum of flavofungin VII (**7**)


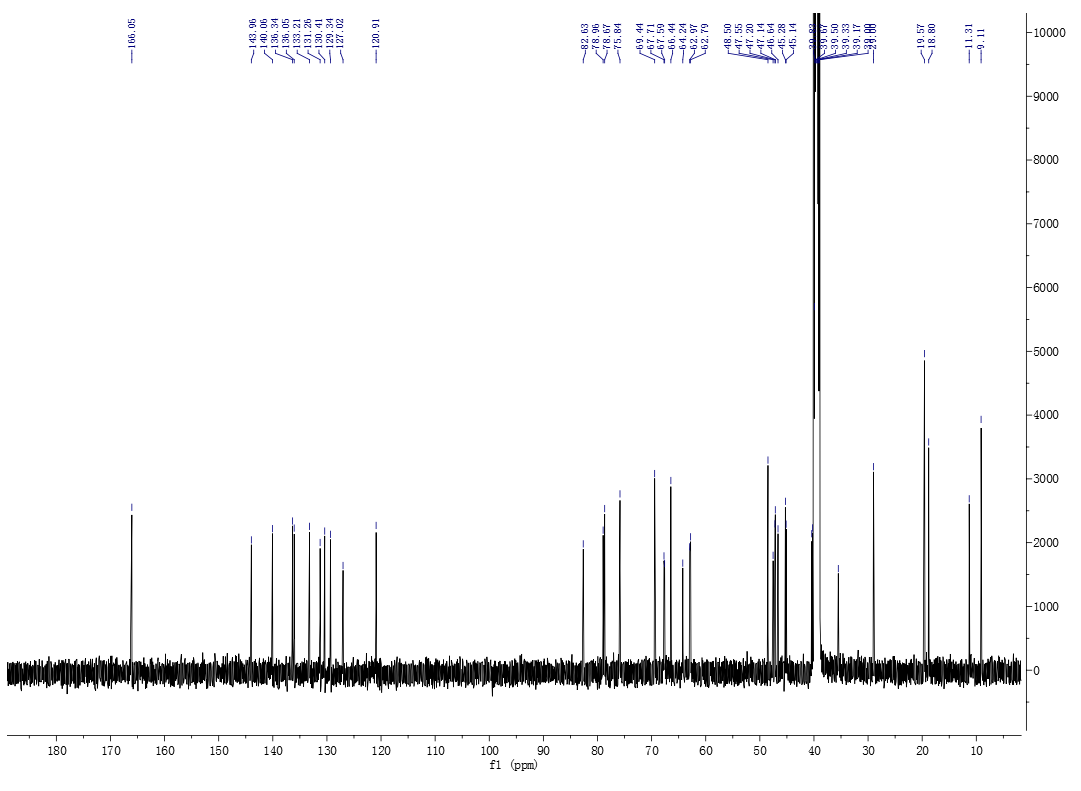


Figure S_80_. ^1^H-^1^H COSY spectrum of flavofungin VII (**7**)


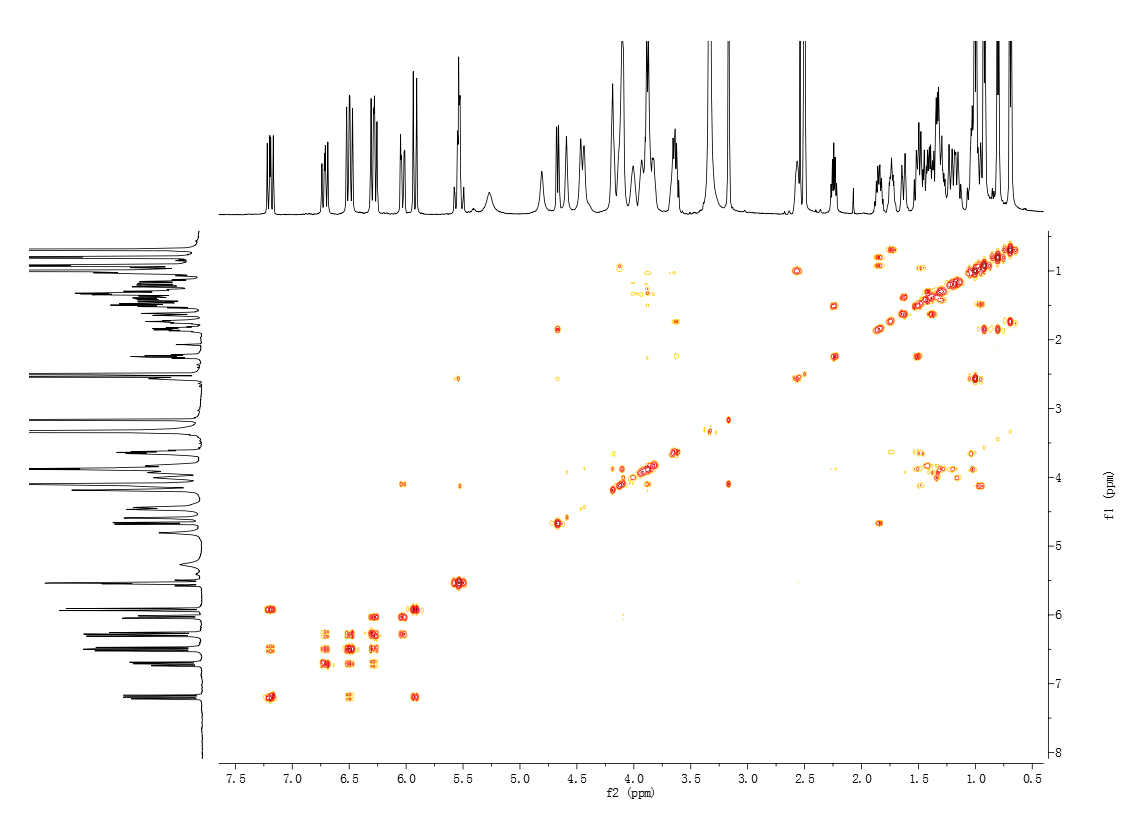


Figure S_81_. HSQC spectrum of flavofungin VII (**7**)


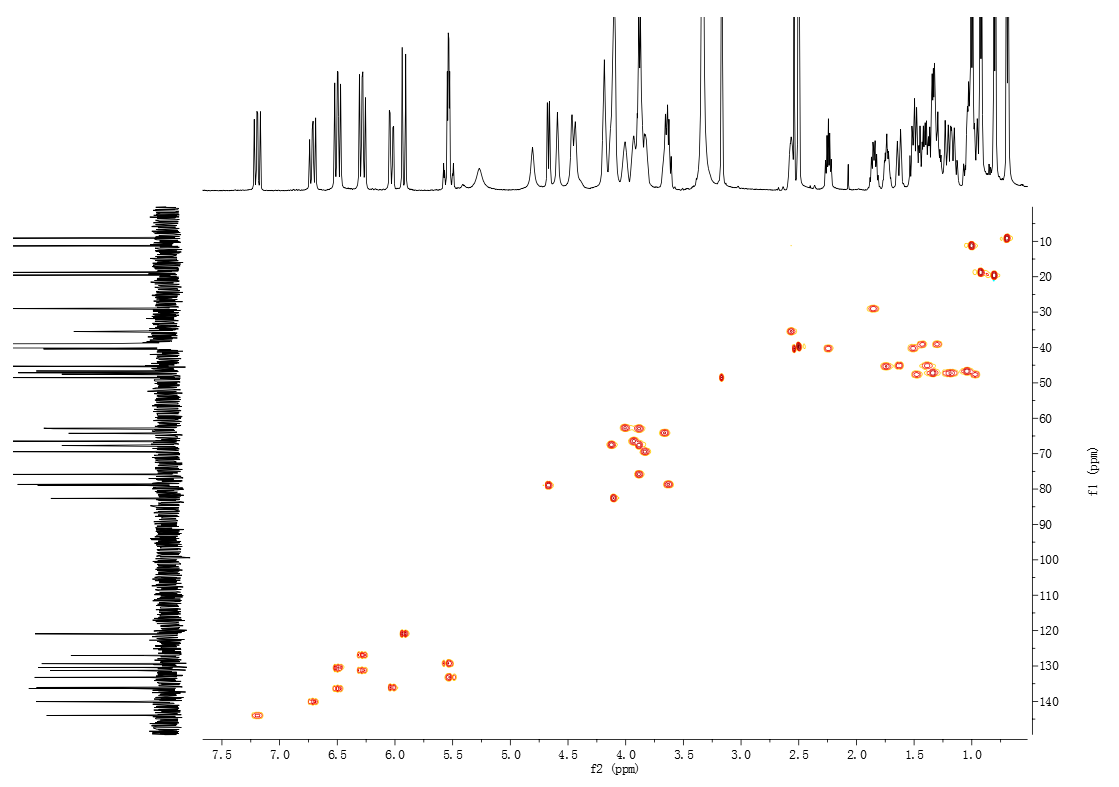


Figure S_82_. HMBC spectrum of flavofungin VII (**7**)


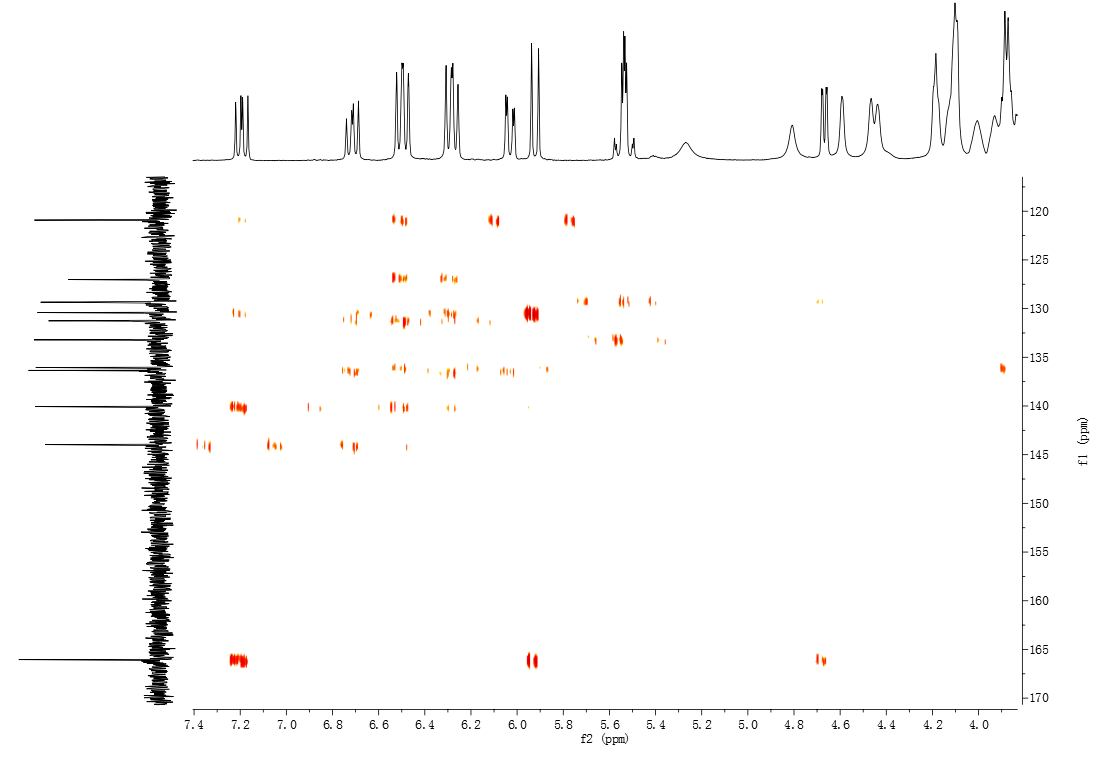


Figure S_83_. HMBC spectrum of flavofungin VII (**7**)


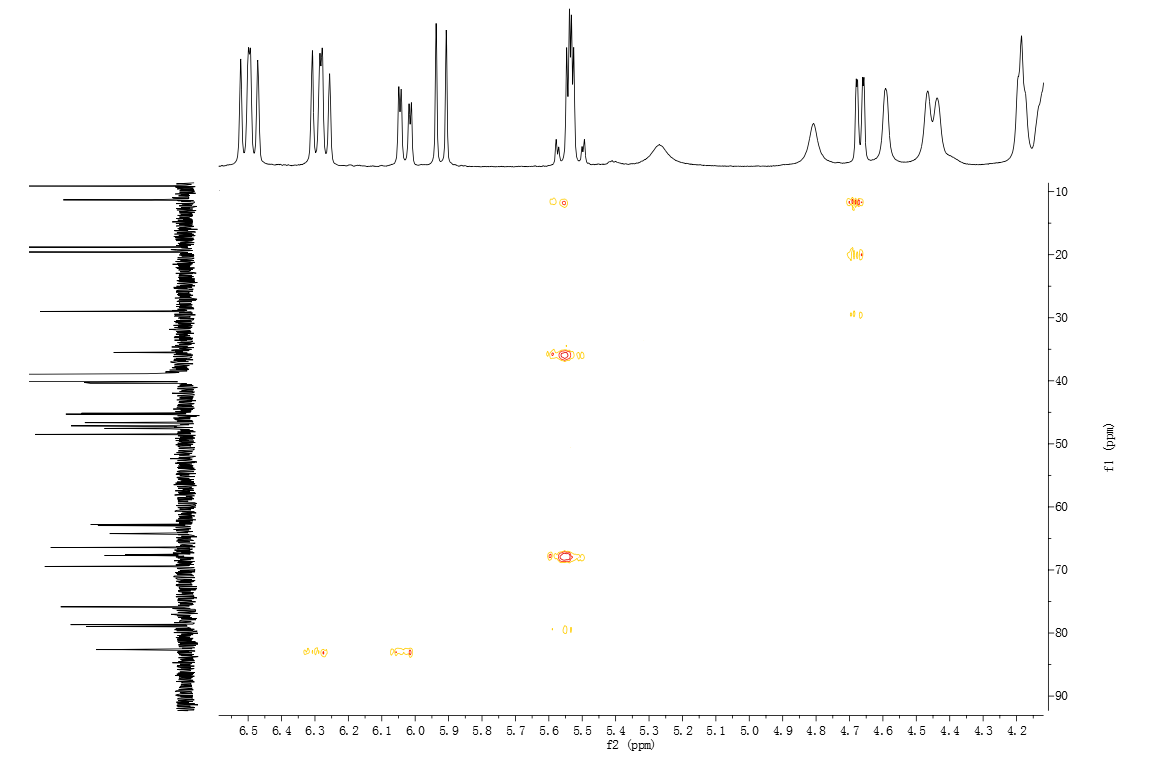


Figure S_84_. HMBC spectrum of flavofungin VII (**7**)


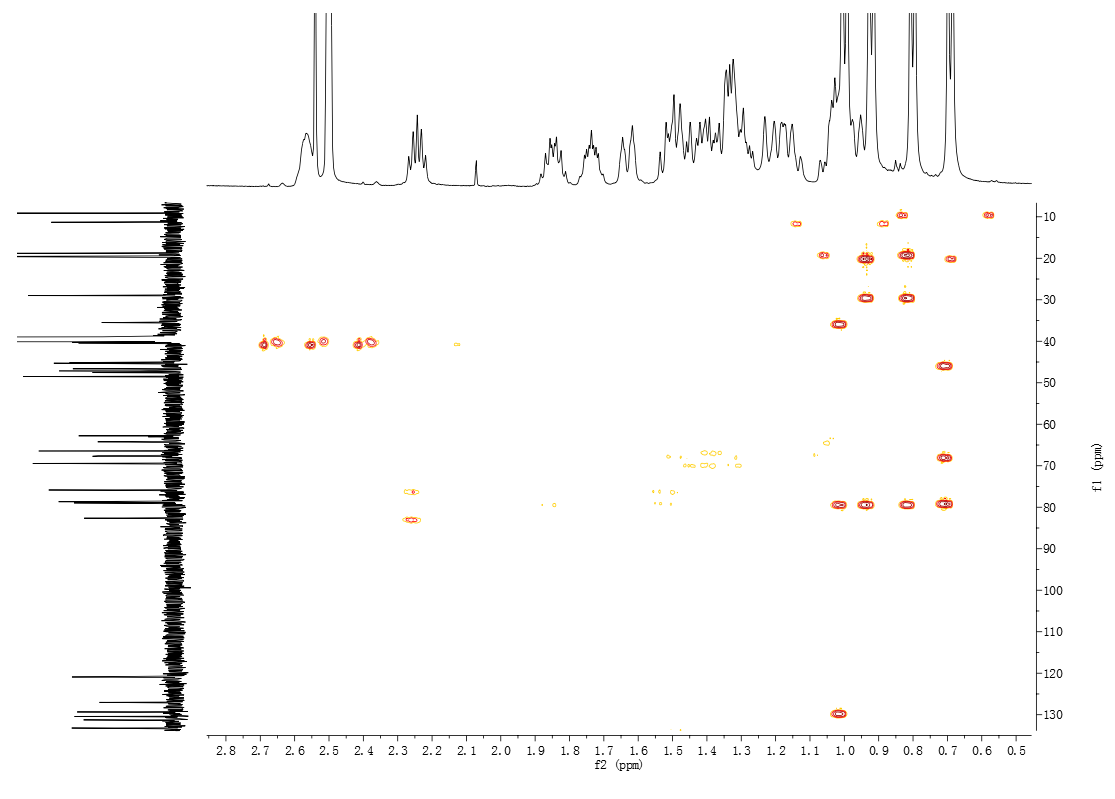


Figure S_85_. NOESY spectrum of flavofungin VII (**7**)


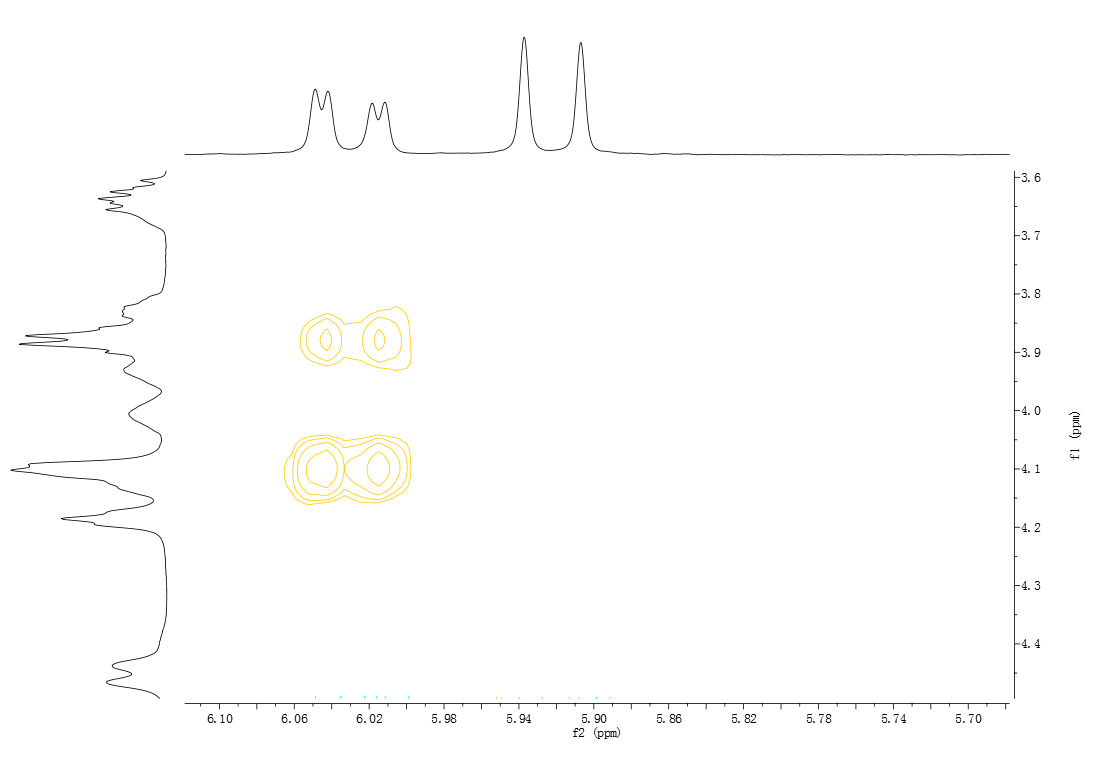


Figure S_86_. HRESIMS of flavofungin VII (**7**)

Figure S_87_. ^1^H-NMR spectrum of flavofungin VIII (**8**)


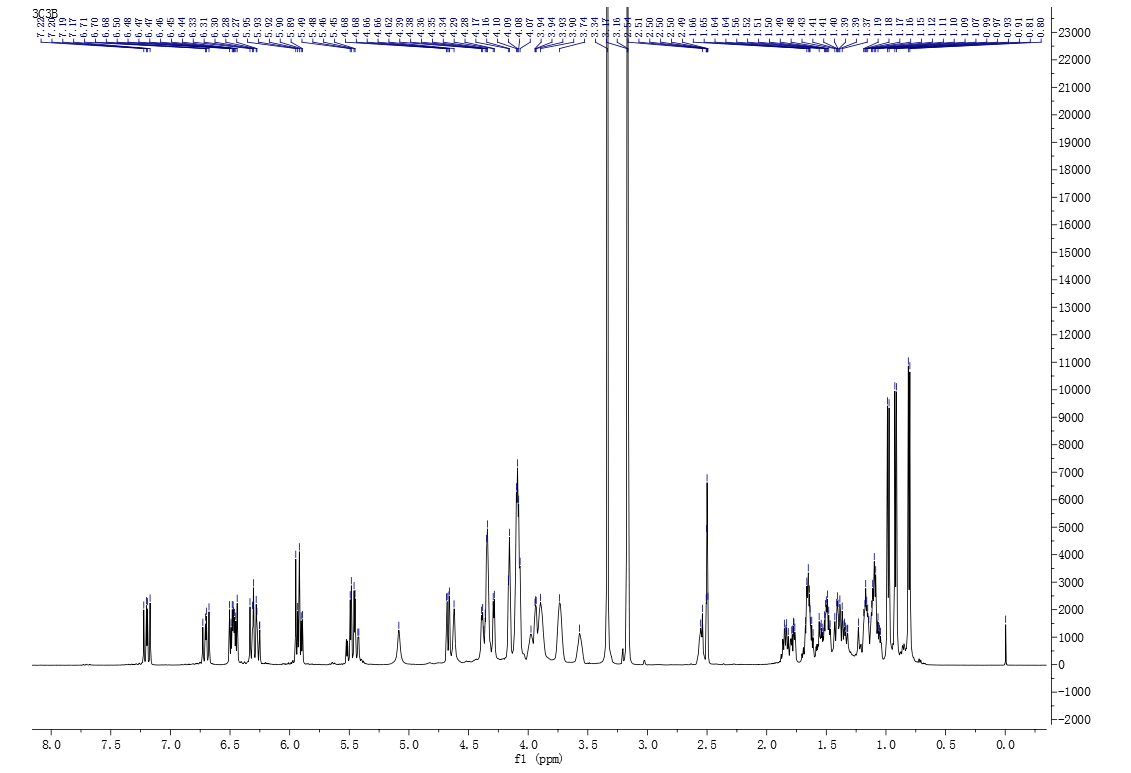


Figure S_88_. ^13^C-NMR spectrum of flavofungin VIII (**8**)


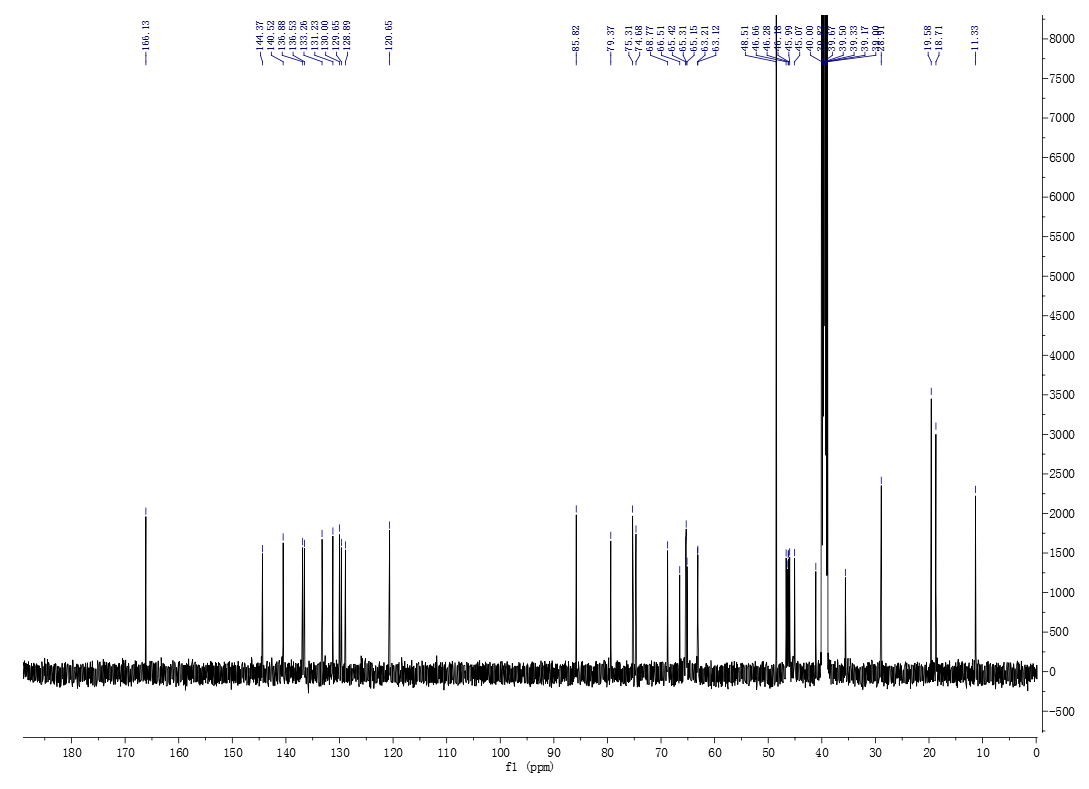


Figure S_89_. ^1^H-^1^H COSY spectrum of flavofungin VIII (**8**)


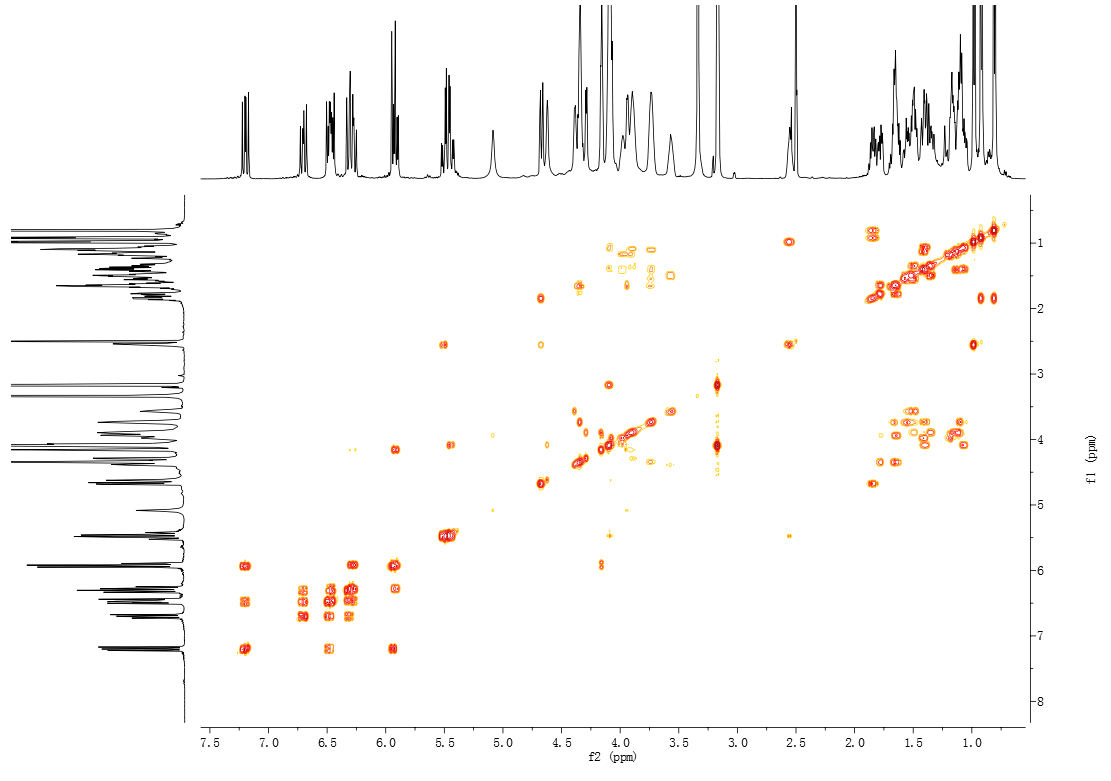


Figure S_90_. HSQC spectrum of flavofungin VIII (**8**)


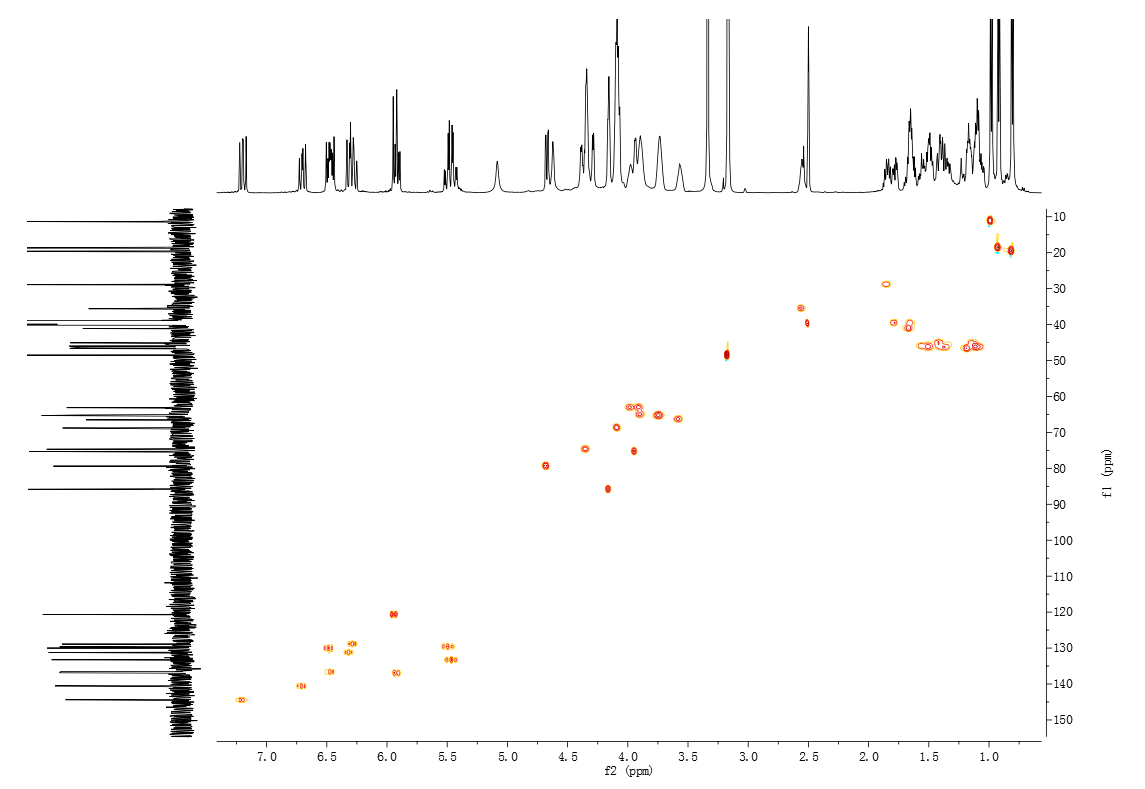


Figure S_91_. HMBC spectrum of flavofungin VIII (**8**)


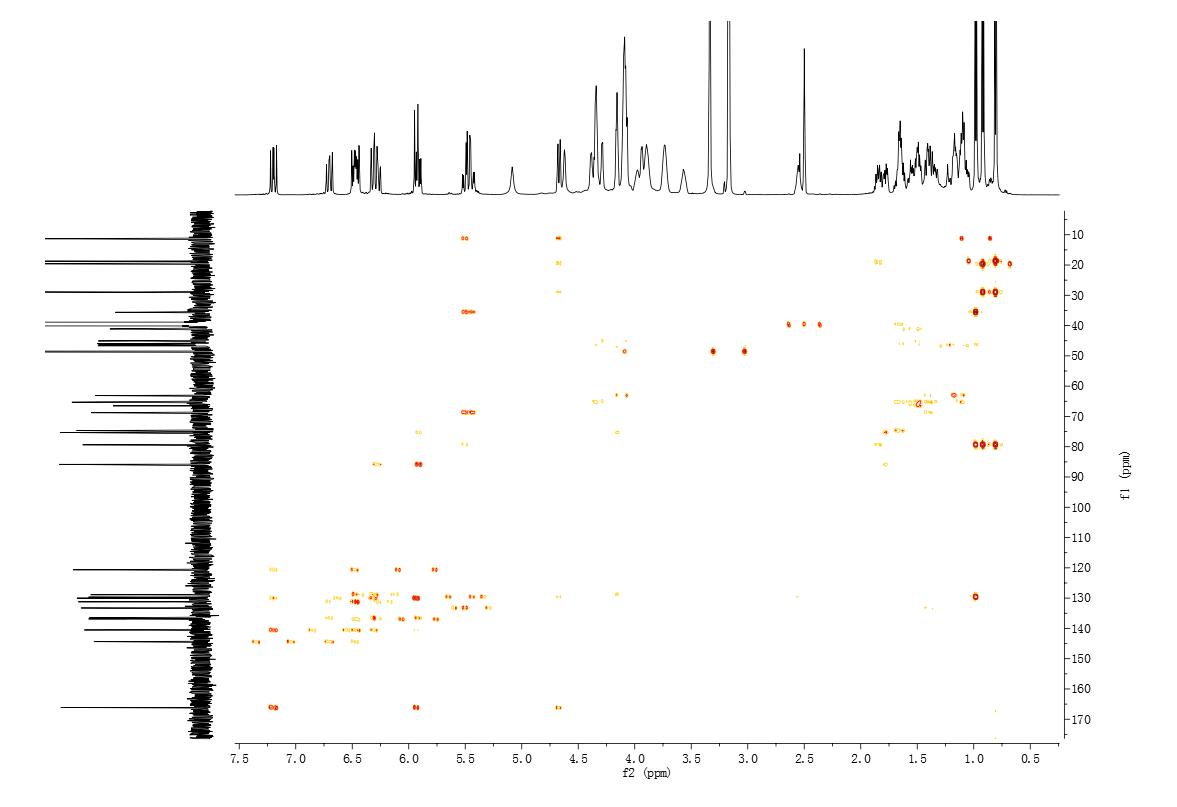


Figure S_92_. HRESIMS of flavofungin VIII (**8**)

Figure S_93_. ^1^H-NMR spectrum of compound **8a**

**
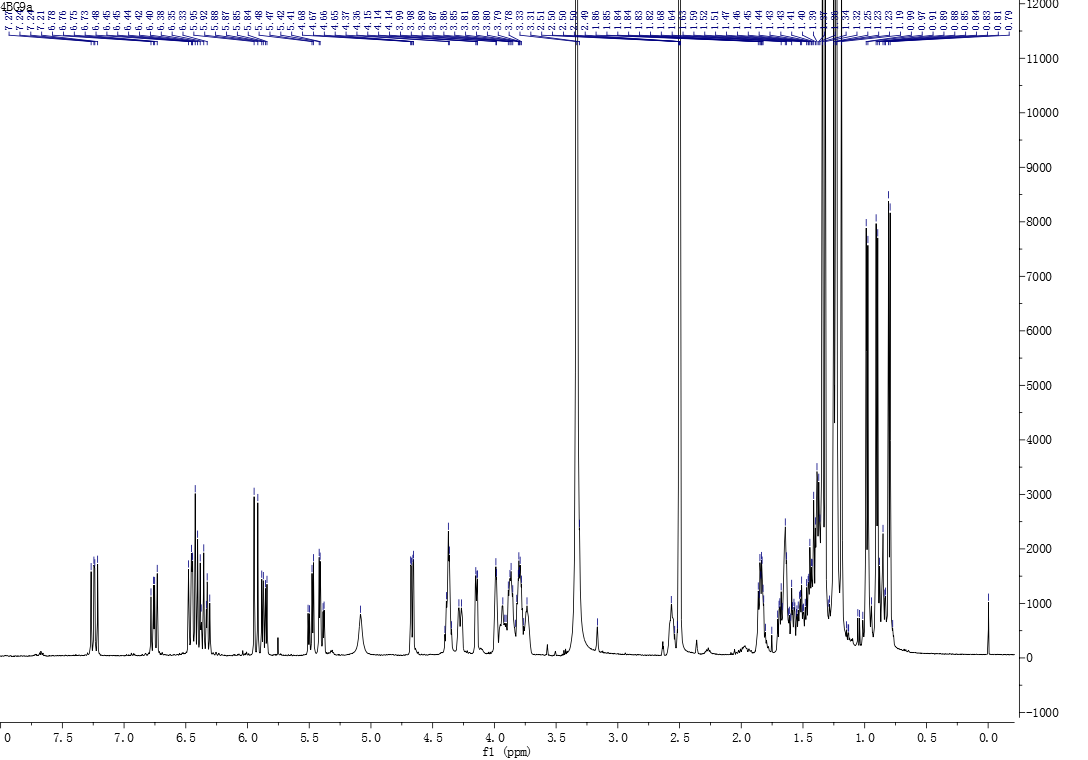
**

Figure S_94_. HSQC spectrum of compound **8a**

**
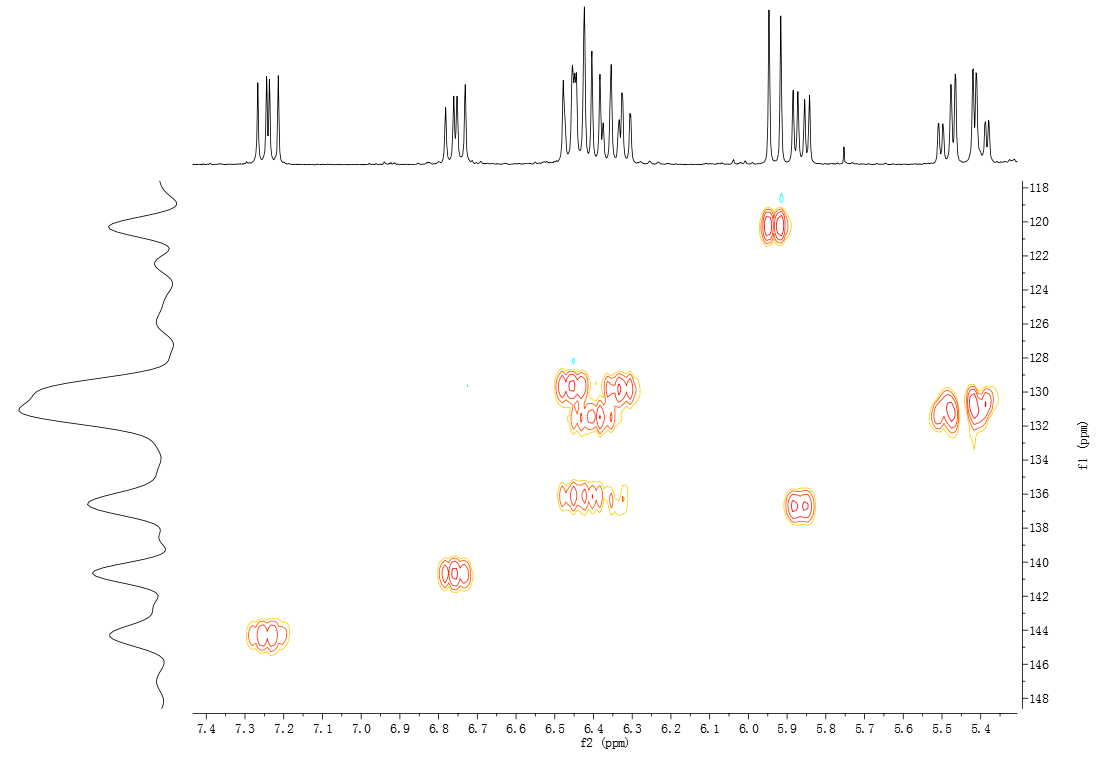
**

Figure S_95_. HSQC spectrum of compound **8a**

**
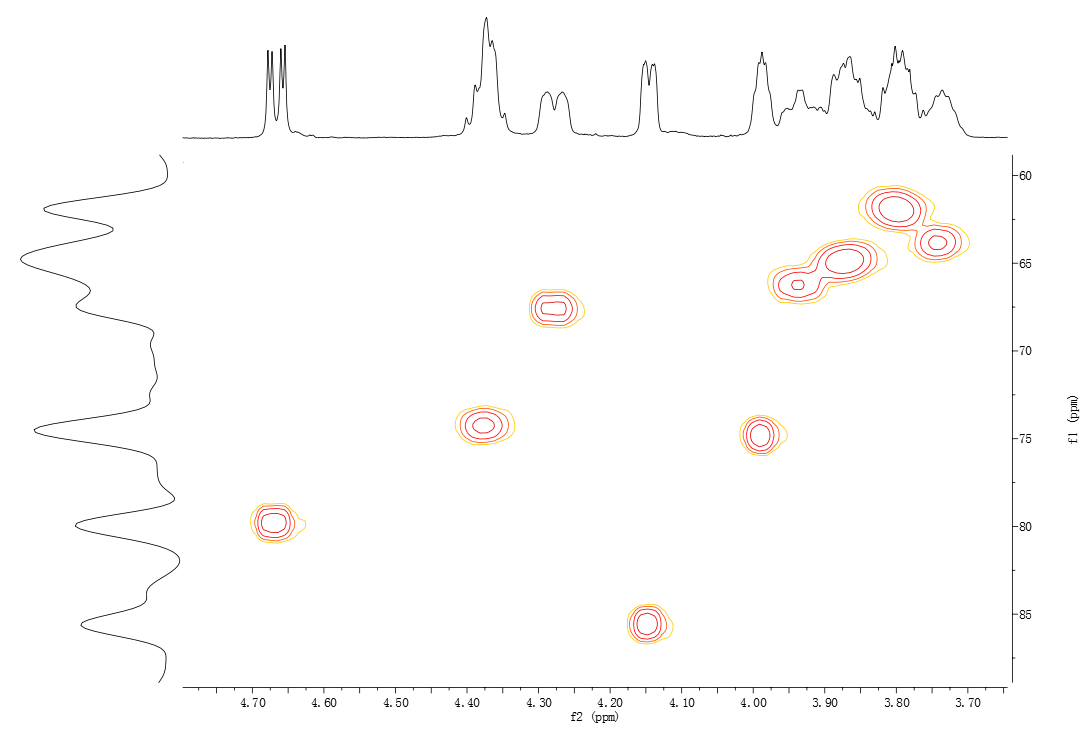
**

Figure S_96_. HSQC spectrum of compound **8a**

**
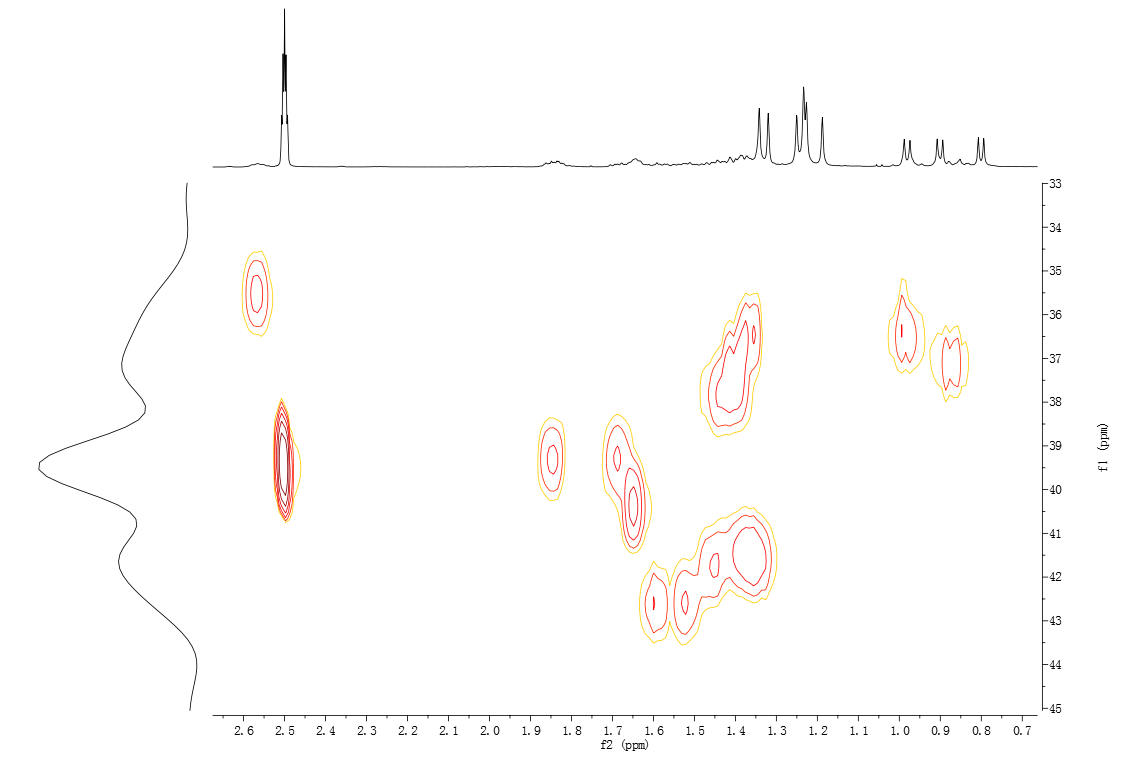
**

Figure S_97_. HSQC spectrum of compound **8a**

**
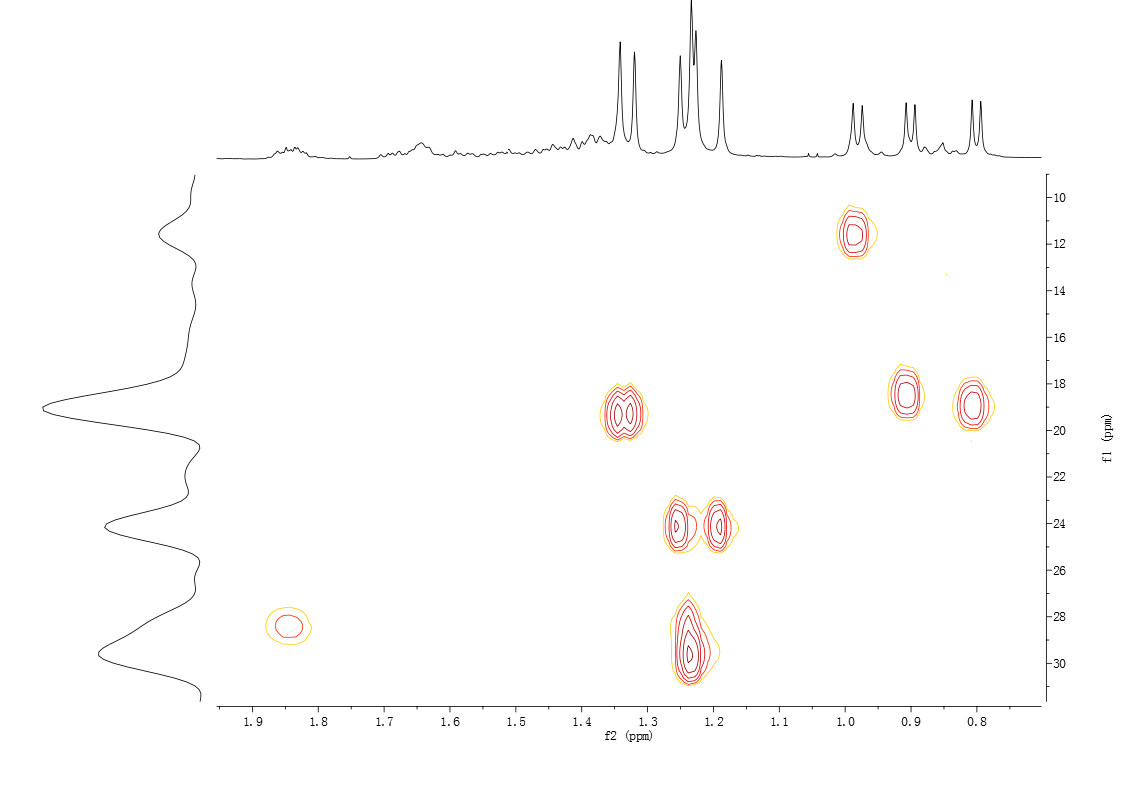
**

Figure S_98_. ^1^H-^1^H COSY spectrum of compound **8a**


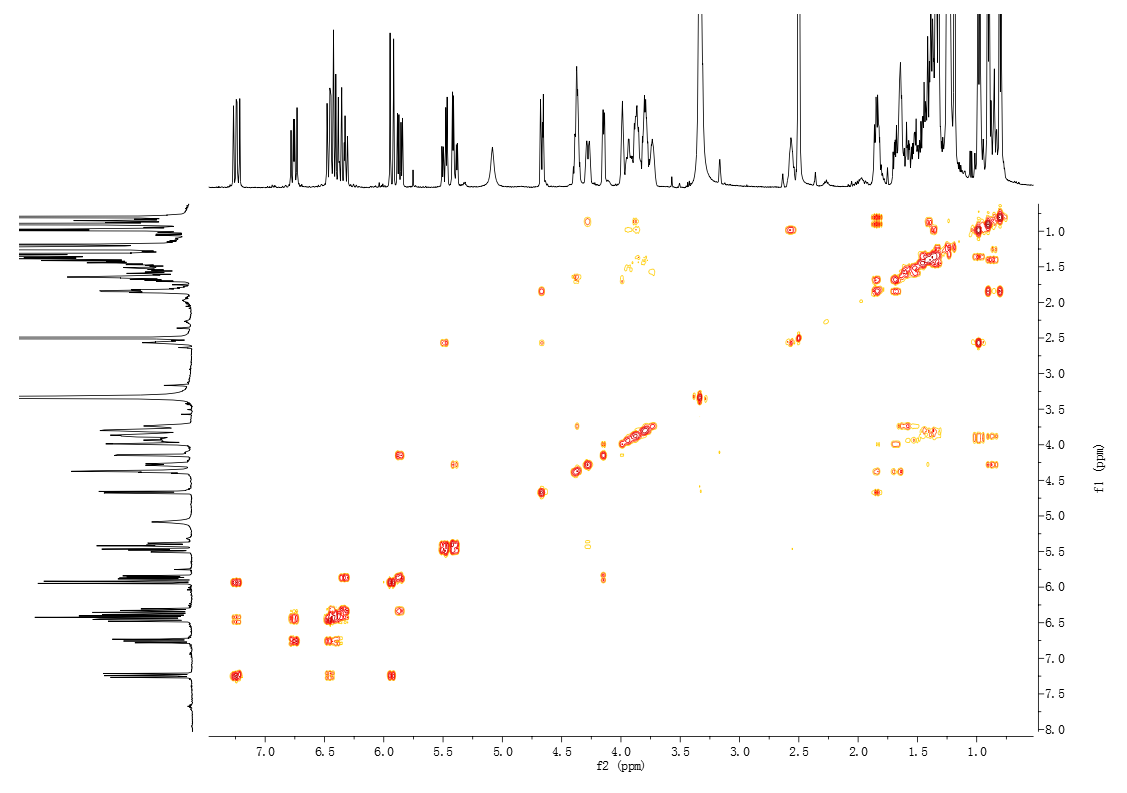


Figure S_99_. HRESIMS of compound **8a**

**
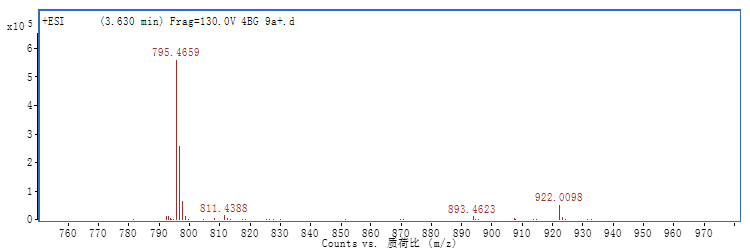
**

Figure S_100_. ^1^H-NMR spectrum of flavofungin IX (**9**)


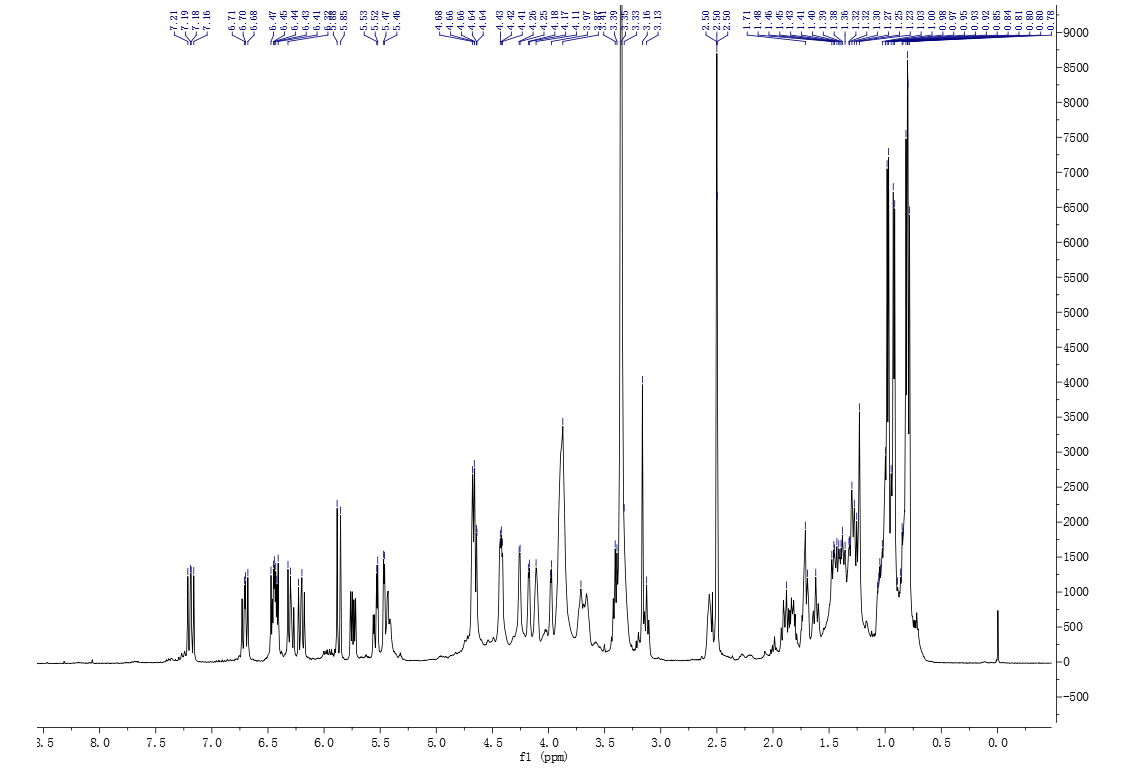


Figure S_101_. ^13^C-NMR spectrum of flavofungin IX (**9**)


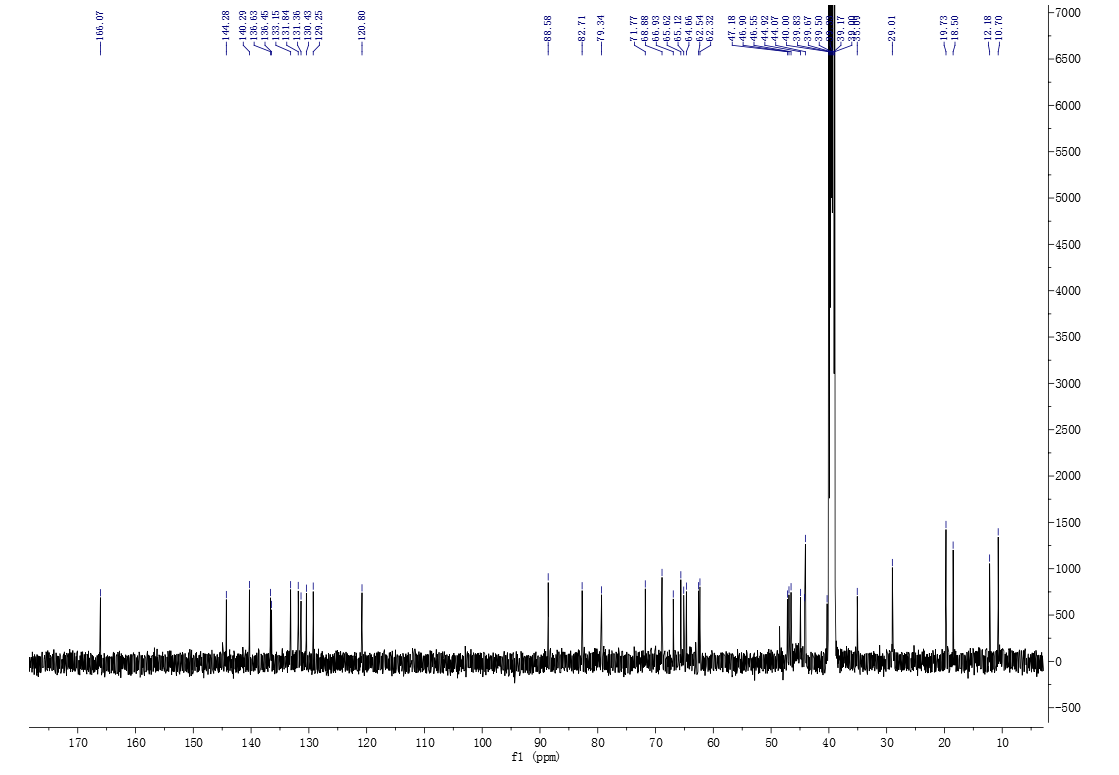


Figure S_102_. ^1^H-^1^H COSY spectrum of flavofungin IX (**9**)


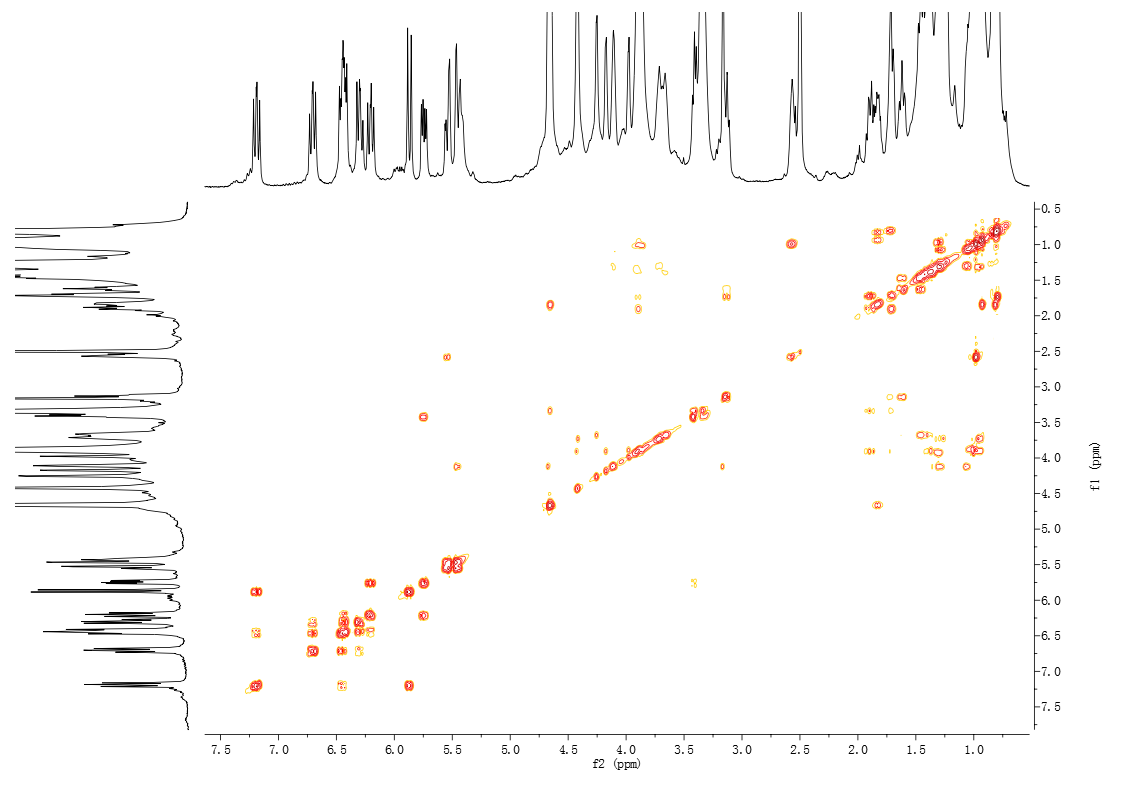


Figure S_103_. HSQC spectrum of flavofungin IX (**9**)


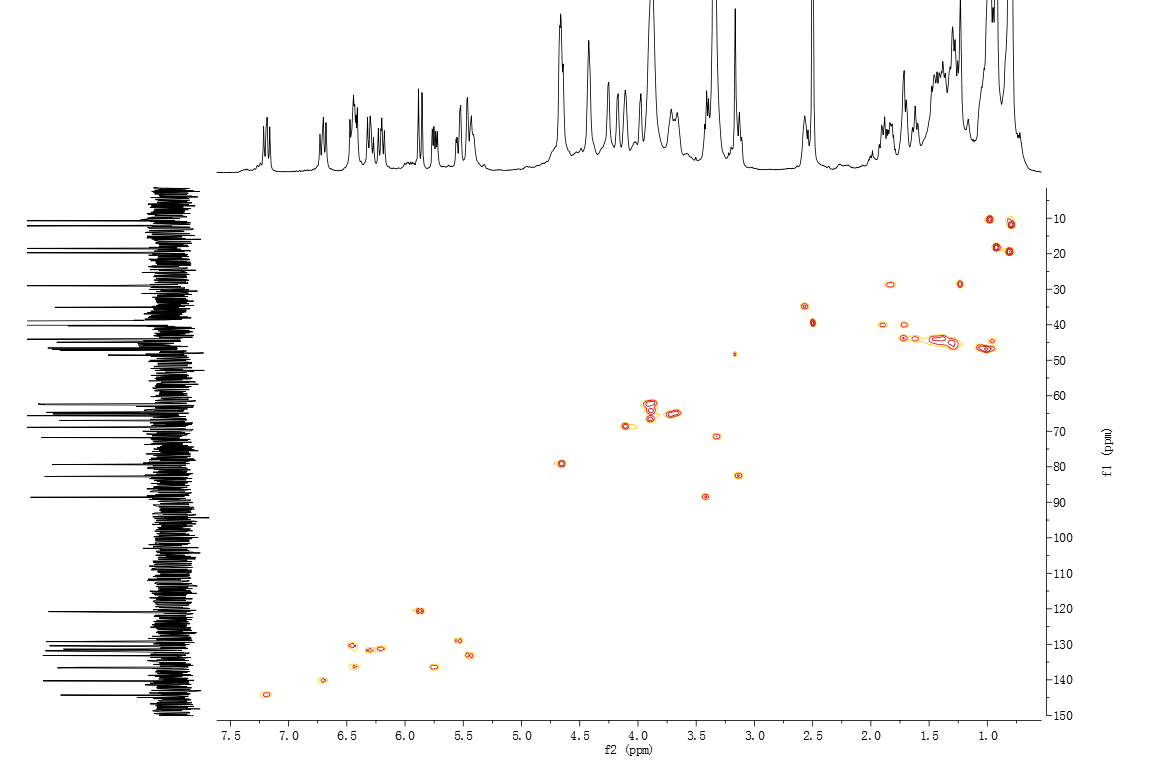


Figure S_104_. HMBC spectrum of flavofungin IX (**9**)


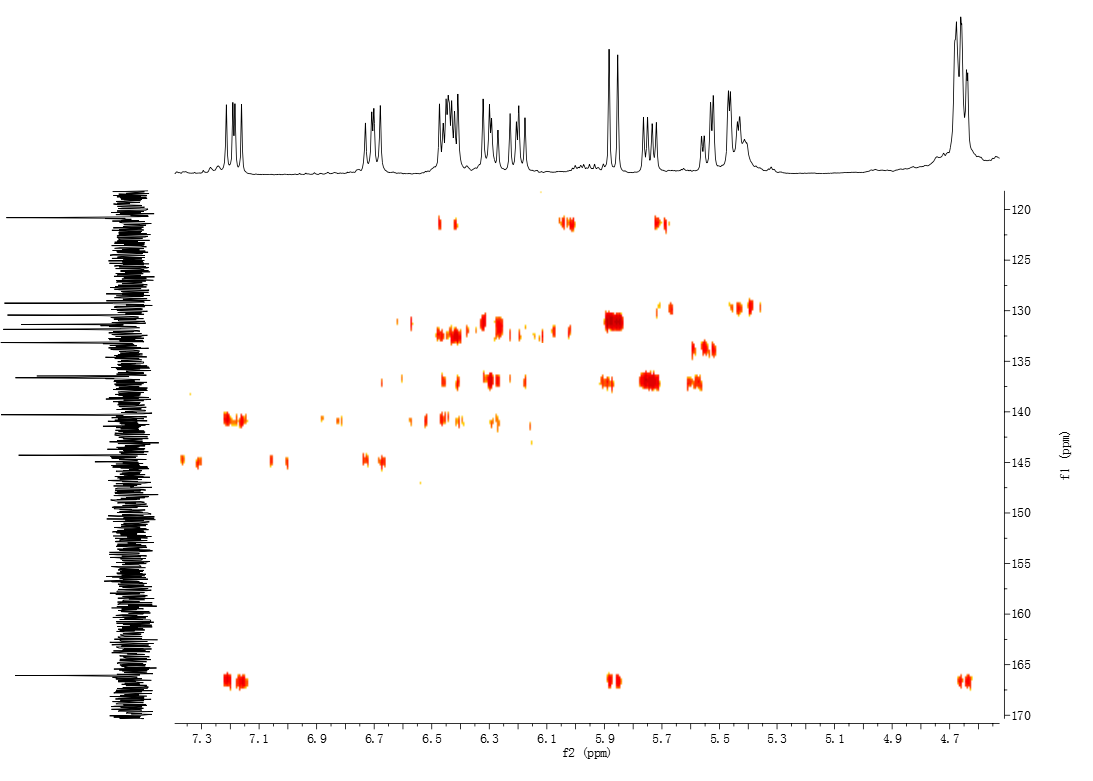


Figure S_105_. HMBC spectrum of flavofungin IX (**9**)


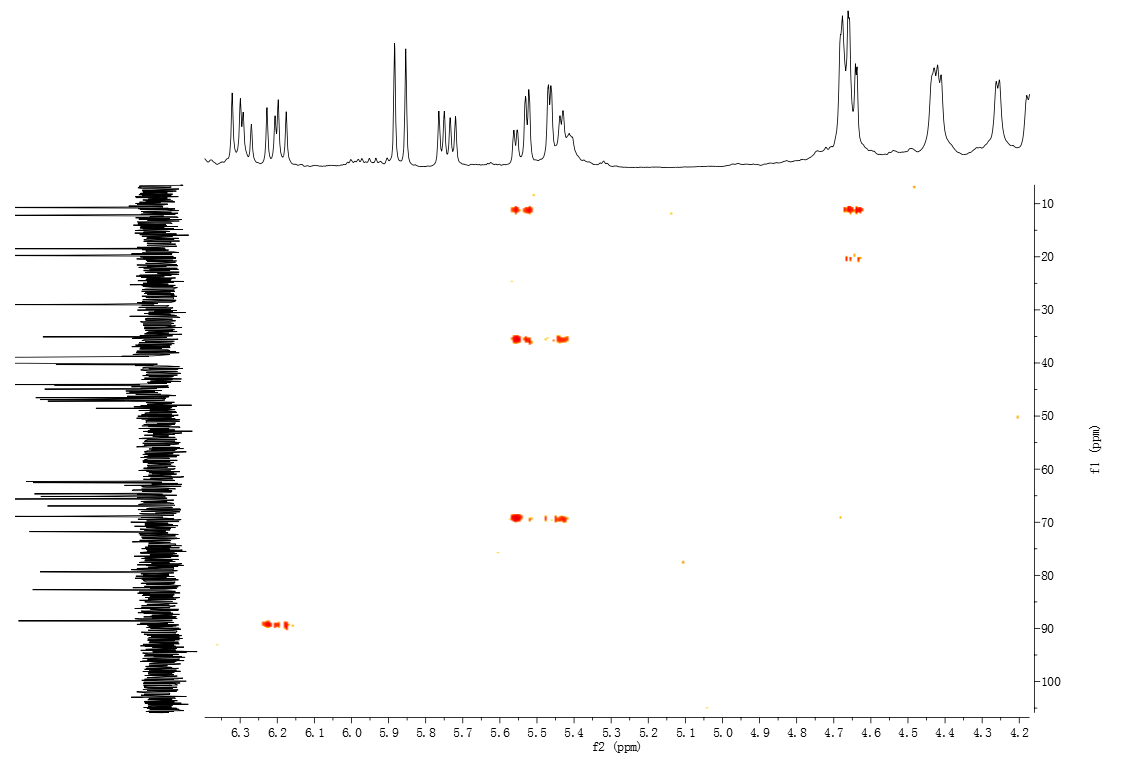


Figure S_106_. HMBC spectrum of flavofungin IX (**9**)


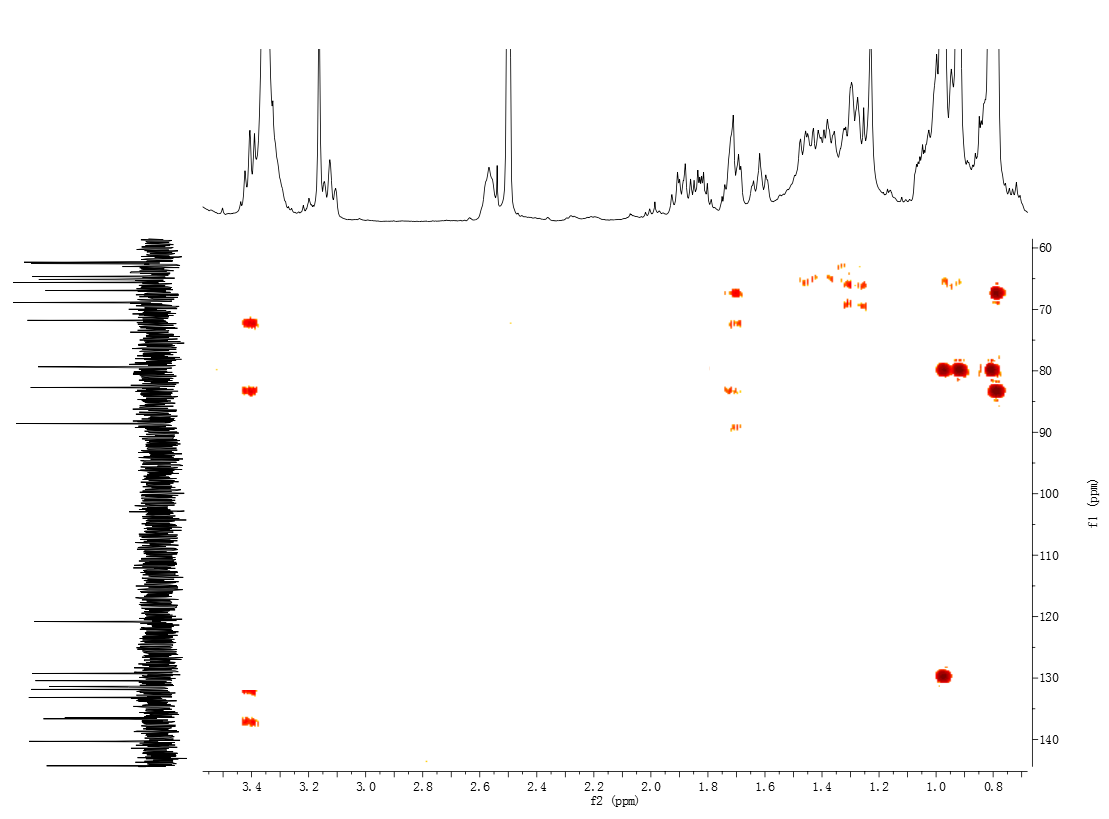


Figure S_107_. HRESIMS of flavofungin IX (**9**)

Figure S_108_. ^1^H-NMR spectrum of compound **9a**


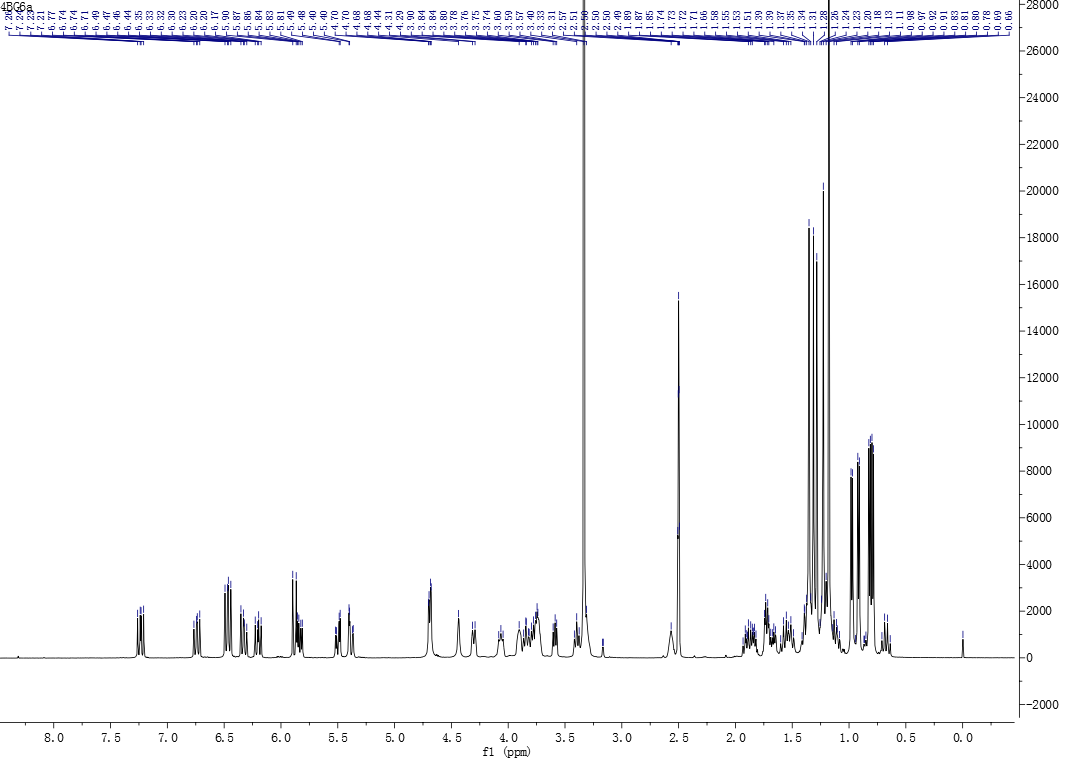


Figure S_109_. ^13^C-NMR spectrum of compound **9a**


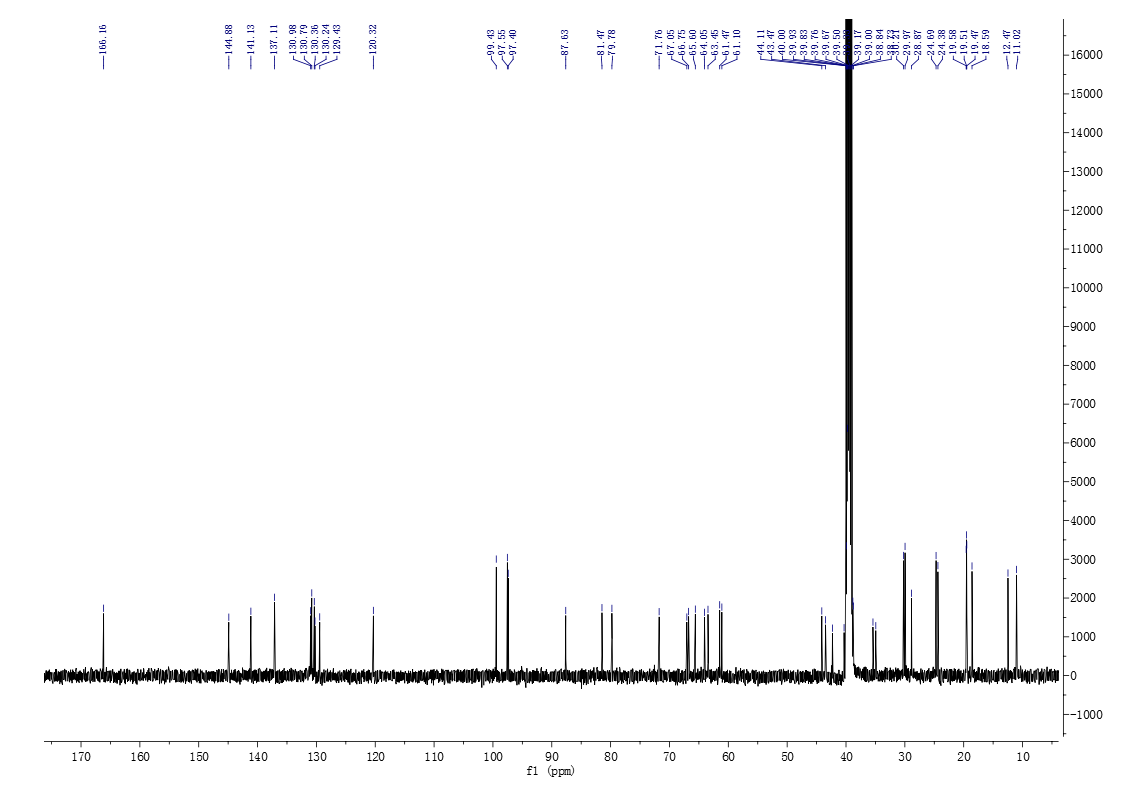


Figure S_110_. HSQC spectrum of compound **9a**

**
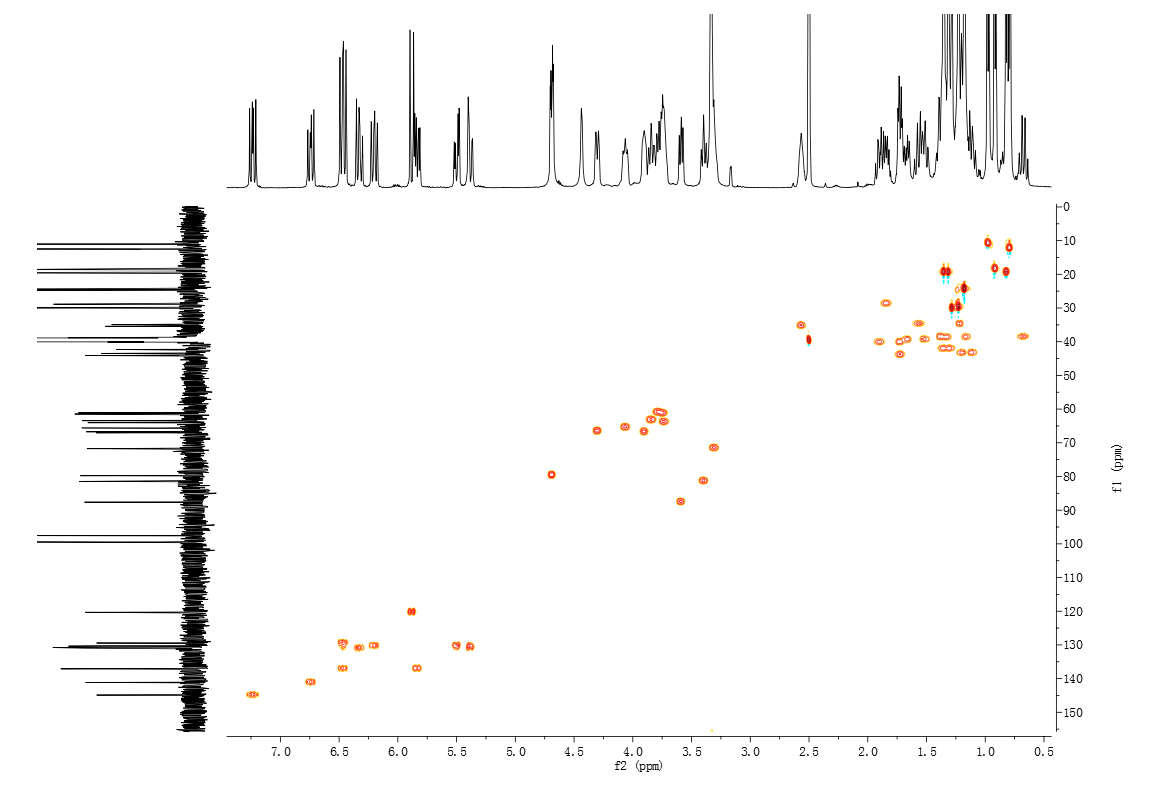
**

Figure S_111_. ^1^H-^1^H COSY spectrum of compound **9a**

**
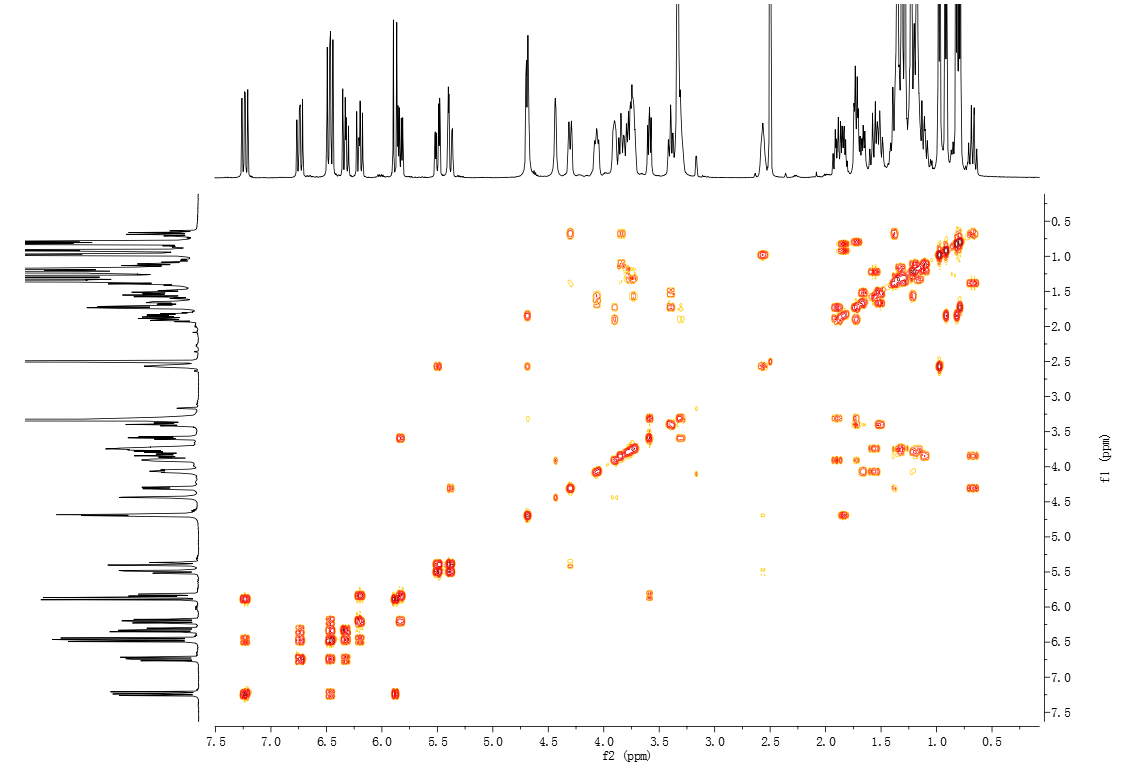
**

Figure S_112_. NOESY spectrum of compound **9a**

**
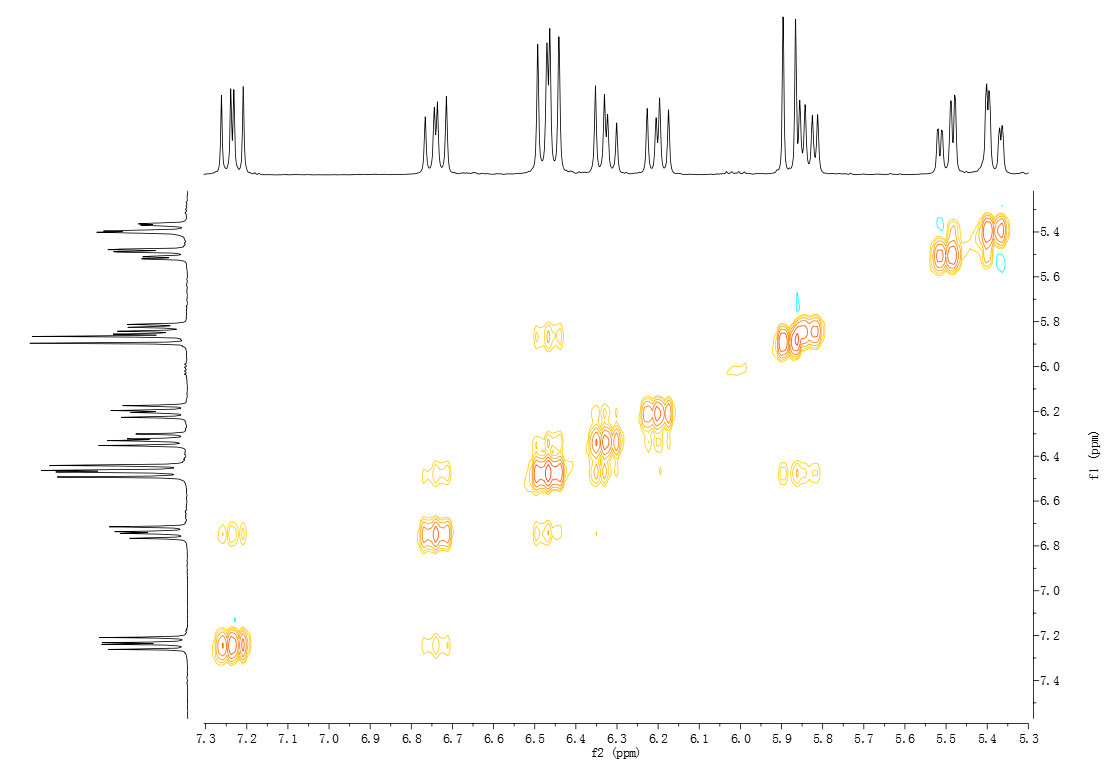
**

Figure S_113_. NOESY spectrum of compound **9a**

**
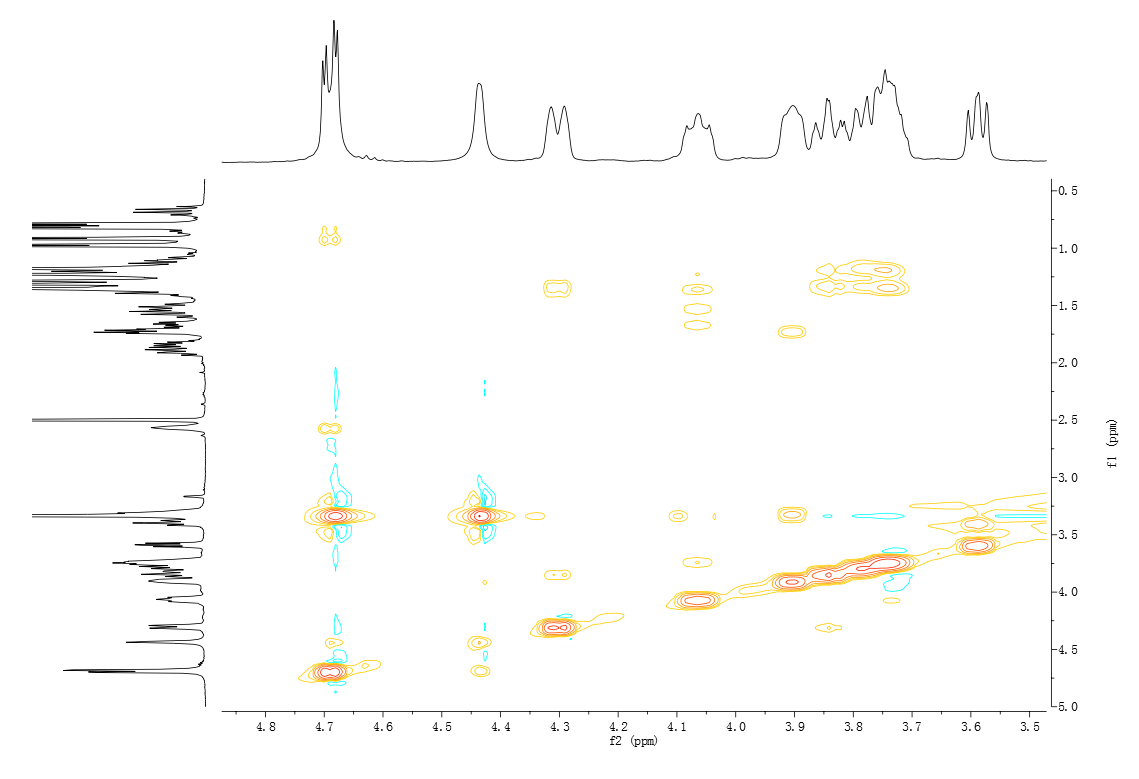
**

Figure S_114_. NOESY spectrum of compound **9a**

Figure S_115_. NOESY spectrum of compound **9a**

Figure S_116_. HRESIMS of compound **9a**

Figure S_117_. ^1^H NMR spectrum of compound **9b**

Figure S_118_. ^13^C NMR spectrum of compound **9b**

Figure S_119_. ^13^C NMR spectrum of compound **9b**

Figure S_120_. ^13^C NMR spectrum of compound **9b**

Figure S_121_. HSQC spectrum of compound **9b**

Figure S_122_. ^1^H-^1^H COSY spectrum of compound **9b**

Figure S_123_. NOESY spectrum of compound **9b**

Figure S_124_. NOESY spectrum of compound **9b**

Figure S_125_. NOESY spectrum of compound **9b**

Figure S_126_. NOESY spectrum of compound **9b**

Figure S_127_. HRESIMS of compound **9b**

Figure S_128_. ^1^H NMR spectrum of compound **9R**

Figure S_129_. HSQC spectrum of compound **9R**

Figure S_130_. ^1^H-^1^H COSY spectrum of compound **9R**

Figure S_131_. HRESIMS of compound **9R**

Figure S_132_. ^1^H-NMR spectrum of compound **9S**

Figure S_133_. HSQC spectrum of compound **9S**

Figure S_134_. ^1^H-^1^H COSY spectrum of compound **9S**

Figure S_135_. ^1^H-^1^H COSY spectrum of compound **9S**

Figure S_136_. HRESIMS of compound **9S**
